# Impact of Biotransformation on Internal Concentrations and Specificity Classification of Organic Chemicals in the Zebrafish Embryo (*Danio rerio*)

**DOI:** 10.1021/acs.est.4c04156

**Published:** 2024-09-24

**Authors:** Nico Grasse, Riccardo Massei, Bettina Seiwert, Stefan Scholz, Beate I. Escher, Thorsten Reemtsma, Qiuguo Fu

**Affiliations:** † Department of Environmental Analytical Chemistry, Helmholtz-Centre for Environmental Research - UFZ, Permoserstrasse 15, 04318 Leipzig, Germany; ‡ Department of Ecotoxicology, Helmholtz-Centre for Environmental Research - UFZ, Permoserstrasse 15, 04318 Leipzig, Germany; § Department of Cell Toxicology, Helmholtz-Centre for Environmental Research - UFZ, Permoserstrasse 15, 04318 Leipzig, Germany; ∥ Environmental Toxicology, Department of Geosciences, Eberhard Karls University Tübingen, Schnarrenbergstr. 94-96, DE-72076 Tübingen, Germany; ⊥ Institute for Analytical Chemistry, University of Leipzig, Linnestrasse 3, 04103 Leipzig, Germany

**Keywords:** toxicity, metabolism, mass balance model, new approach methodologies, bioaccumulation

## Abstract

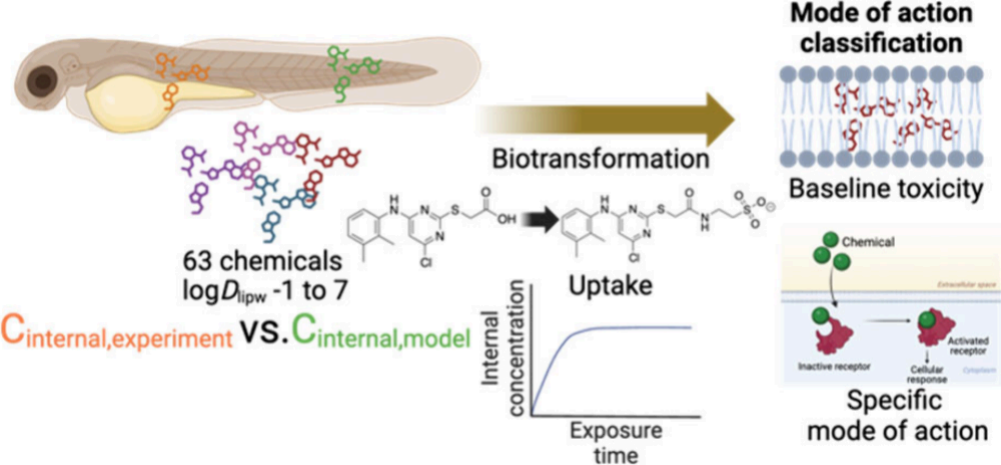

Internal concentrations (ICs) are crucial for linking exposure to effects in the development of New Approach Methodologies. ICs of chemicals in aquatic organisms are primarily driven by hydrophobicity and modulated by biotransformation and efflux. Comparing the predicted baseline to observed toxicity enables the estimation of effect specificity, but biological processes can lead to overestimating ICs and bias the specificity assessment. To evaluate the prediction of a mass balance model (MBM) and the impact of biotransformation on ICs, experimental ICs of 63 chemicals in zebrafish embryos were compared to predictions with physicochemical properties as input parameters. Experimental ICs of 79% (50 of 63) of the chemicals deviated less than 10-fold from predictions, and the remaining 13 deviated up to a factor of 90. Using experimental ICs changed the classification for 19 chemicals, with ICs 5 to 90 times lower than predicted, showing the bias of specificity classification. Uptake kinetics of pirinixic acid, genistein, dexamethasone, ethoprophos, atorvastatin, and niflumic acid were studied over a 96 h exposure period, and transformation products (TPs) were elucidated using suspect- and nontarget screening with UPLC-HRMS. 35 TPs (5 to 8 TPs per compound) were tentatively identified and semiquantified based on peak areas, suggesting that biotransformation may partly account for the overpredictions of ICs.

## Abstract 2

Comparison of internal concentrations with predicted baseline toxicity identified chemicals with deviations due to metabolism or specific effects. Thus, toxicokinetics are important for the interpretation of effects and assessment of their specificity.

## 1 Introduction

The need of 3R (Replacement, Reduction, and Refinement) compliant testing platforms has encouraged research involving nonmammalian model organisms, e.g., the zebrafish embryo (*Danio rerio*) (ZFE) for risk assessment of chemicals.^1,2^ For these applications, it is fundamental to understand the mode of action (MOA) of a chemical. The baseline toxicity of a chemical represents the minimal, unspecific toxicity driven by hydrophobicity and is used to assess the specificity of an observed toxic effect of a chemical by deviation from baseline toxicity.^3−7^ Baseline toxicity of organic chemicals can be predicted from descriptors of the hydrophobicity of a compound such as the membrane lipid/water distribution ratio (log*D*_lipw_). Compounds with a specific or reactive MOA would exhibit a significantly higher toxicity compared to their predicted baseline toxicity. This is expressed as the toxic ratio (TR), i.e., the ratio of the predicted baseline toxicity to the experimentally determined half-maximal lethal concentration (LC_50_). TRs around 1 (0.1 < TR < 10) are considered as an indicator that a compound’s toxicity is mainly driven by baseline toxicity. Chemicals with a TR > 10 indicate a specific or reactive MOA which could represent a particular hazard of a chemical.^8^ ATR below 0.1 could indicate artifacts of bioavailability or strong internal degradation. Internal concentrations (ICs) are the biologically effective doses and vital to know in order to interpret toxic effects. Consequently, experimental IC data would improve chemical hazard assessment by considering the absorption, distribution, metabolism, and elimination (ADME) of chemicals in the organism. ICs are often predicted by modeling approaches (e.g., mass balance^3,5,6,9^ and steady state ion-trap models^3^) assuming passive, nonspecific uptake across biological membranes. In the ZFE, gills the gastrointestinal tract and the cardiovascular system are not yet relevant for uptake and distribution of chemicals as the ZFE feeds from the yolk within the first 5 days post fertilization (hpf)^10^ and chemicals are taken up mainly via the skin.^11^ However, the IC of a chemical may be modulated by transformation and transport processes, which is not easy to account in mass balance models. Thus, for some chemicals, the biologically available internal dose can be overestimated when the toxicokinetics of the parental compound is not considered.^12^ This would lead to underestimation of specific toxicity, which could lead to a wrong classification of chemicals. Ideally, measured ICs in combination with experimental physicochemical properties would avoid artifacts and substantially improve the quality of the prediction for IC and, thus, the specificity assessment of a toxic effect.

To emphasize the need for experimental toxicokinetic data for specificity assessments, the ICs of 63 chemicals in ZFEs were experimentally determined and compared with the concentrations predicted by a mass balance model (MBM). Additionally, for compounds showing a significant deviation from the MBM, uptake kinetics and biotransformation products were determined to evaluate the biotransformation in the ZFE and its impact on chemical MOA classification. The mechanistic and quantitative knowledge of the biotransformation of compounds in the ZFE would improve our understanding of how biological processes influence the internal dose and the toxic effects that may occur.

## 2 Materials and Methods

### 2.1 Chemicals, Reagents, Standards

All chemicals were of analytical grade and are provided in the Supporting Information (SI, Excel File, sheet S1). For HPLC-MS experiments, methanol (MeOH) (>99%) and formic acid (99%) were obtained from Biosolve (Valkenswaard, Netherlands). A Merck Milli-Q Integral 5 system (Merck, Darmstadt, Germany) was used to generate ultrapure water. The respective suppliers and qualities of the test chemicals can be found in the SI (Excel file, sheet S1). Details on the preparation of exposure media are provided in sections S1.3 and S1.4 in SI. Chemical standard solutions were prepared as 1 mg/mL in MeOH and used for both chemical exposure and quantification. Stock solutions were stored at −20 °C.

### 2.2 Chemical Selection

Test chemicals were selected according to diversity of their modes of action, physicochemical properties, environmental relevance and availability as part of the EU Horizon 2020 project PrecisionTox.^13^ In this study, chemicals cover a log*D*_lipw_ range from −1.0 to 6.8. In total, 38 nonionic, 14 cationic, and 11 anionic chemicals were selected to test the applicability of the MBM for different chemical species. Molecular weights ranged from 68 to 622 Da. The physicochemical properties of all test compounds are provided in the SI (i.e., Excel File, sheet S1).

### 2.3 Dissociation Constants and Liposome- and Structural Protein−Water Distribution

The prediction of ICs by the MBM as well as the toxic ratio analysis requires as input parameter the liposome- and structural protein−water distribution ratios (*D*_lipw_ and *D*_SPw_). The dissociation is important for the charge of the compound and the uptake of the compound into the ZFE. They were previously collected from experimental literature data or measured/estimated by Huchthausen et al.^14^ (SI, Excel File, sheet S1).

### 2.4 Exposure of Zebrafish Embryos

#### 2.4.1 Fish Embryo Toxicity Test (FET)

Details on fish husbandry can be found in SI (section S1.2). LC_50_ values were obtained from the literature or were generated in this study by exposing fish embryos in a 96 well plate to a series of concentrations as described by Teixido et al. 2022^15^ (SI, table S2). Briefly, ZFEs at 4 hpf were exposed to the individual chemical for 96 h on 96-well plates in aqueous media in accordance with DIN EN ISO 7346−3 (1997) (ISO water) containing 10 mM of (4-(2-hydroxyethyl)-1-piperazineethanesulfonic acid) buffer at pH 7.4. The exact composition of ISO water and the calculation of neutral fractions of the test chemicals are provided in SI (section S1.3 and S2). Four end points were recorded as indicators of lethality every 24 h: (i) coagulation of fertilized eggs, (ii) lack of somite formation, (iii) lack of detachment of the tail-bud from the yolk sac, and (iv) lack of heartbeat. At the end of the exposure, acute lethality was determined based on any of the four apical observations recorded, and the LC_50_ was calculated based on a log−logistic concentration−response model using the R-package drc (i.e., SI, Excel File, sheets S1 and S2). References for LC_50_ data obtained from the literature^16^ are provided in the SI (i.e., Excel file, sheet S1).

#### 2.4.2 Internal Concentration and Uptake Kinetics Assessment

Previous data indicated a linear correlation of external and internal concentrations over a wide concentration range.^17−19^ Additionally, we measured internal concentrations of atorvastatin at five different exposure concentrations to demonstrate this linear releationship. This example is shown in **Figure S7** in SI. Therefore, ICs were assessed for only one test concentration. To avoid any bias by toxicity on ICs, the exposure concentrations were selected 10 to 100 times lower than the experimental LC_50_. The nominal and measured external concentrations of each chemical can be found in SI (i.e., Excel File, sheet S3). For the screening of ICs, the exposure was conducted for 96 h at 26 ± 1 °C. For uptake kinetics experiments, samples of exposed ZFEs were collected after 24 h, 48 h, 72 and 96 h. As a negative control, fertilized eggs were exposed to ISO water containing 0.1% MeOH without any test chemicals to check any possibilities of chemical contamination during the whole process. Dead ZFEs were removed daily to avoid microbial growth. Exposure medium was collected at every sampling time point to confirm concentrations of test chemicals. Medium samples were stored at −20 °C until HPLC-MS/MS analysis. Further details on toxicokinetic experiments are given in SI (section S1.5).

#### 2.4.3 Chemical Stability Assessment in Assay Media

The exposure media were incubated with chemicals but without ZFEs to assess the chemical stability. To evaluate the adsorption of the chemical by, e.g., plastic, incubation was performed on 96-well plates under the same conditions as those in the exposure experiment with ZFEs. In parallel, incubation was performed in glass vials to characterize the chemical stability, assuming no significant adsorption to the glass vials. Chemical stability was assessed at initial concentrations of 1 mg/L. After 96 h, the chemicals were pooled and diluted with ultrapure water to the calibration range of each chemical. Exposure media samples were stored at −20 °C until chemical analysis. Results from chemical stability assessment can be found in the SI (**Figure S1**).

### 2.5 Sample Preparation for Chemical Analysis

Unexposed and ZFEs exposed to chemicals were collected in FastPrep tubes filled with glass beads (8 ZFEs per replicate). If ZFEs had not already hatched, embryos were manually dechorionated before sampling. The exposure medium was removed, and the embryos were rinsed three times with ISO water. Subsequently, all liquid was removed, and the ZFEs were snap-frozen with liquid nitrogen and stored at −80 °C until further processing. Analytes were extracted from the embryos using 200 μL MeOH according to Halbach et al.^10^ The ZFEs were homogenized using a FastPrep homogenizer (MP Biomedicals, USA) for 20 s at a rate of 6.5 m/s. Then the homogenates were sonicated for 30 min at room temperature (around 20 °C). According to literature,^10,12,20,21^ a significant degradation of TPs during this procedure is not expected. In the next step, the samples were centrifuged (13000 rpm, 4 °C, 15 min) and 150 μL of the supernatants were transferred to HPLC glass vials containing 300 μL inserts. The extracts were stored at −20 °C until LC-MS analysis.

### 2.6 Determination of External and Internal Chemical Concentrations

#### 2.6.1 Measurement of External and Internal Concentrations Using LC-MS/MS

The concentrations of the chemicals in the ZFE extracts and the exposure media were determined by liquid chromatography coupled with tandem mass spectrometry (LC-MS/MS). Method validation data, including limits of detection, limits of quantification, linear dynamic range, matrix effects, method recoveries, and instrumental parameters are provided in the SI, section S6.1.3.

#### 2.6.2 Calculation of Bioconcentration Factors (BCFs) Based on Experimental Data

Experimental BCFs were obtained after 96 h of exposure, assuming a steady state of ICs as demonstrated in previous studies for many chemicals.^12^ This value can be calculated by division of the obtained experimentally determined IC by the concentration measured in the external aqueous solution (C_w_) (eq 1).
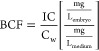
1

### 2.7 HRMS Analysis and Identification of Transformation Products (TPs)

The detection and identification of TPs was conducted using UPLC-QTOF-MS according to Grasse et al.^22^ using suspect- and nontarget screening approach. MarkerLynx was used for the feature analysis of potential TPs of chemicals formed in the ZFE. Detailed instrumental and software parameters are provided in the Supporting Information (section S6.2, Tables S7 and S8). Suspect screening was performed using predicted metabolization pathways provided by Biotransformer 3.0.^23^

### 2.8 Prediction of ICs Using a Mass Balance Model (MBM)

To identify chemicals with increased metabolic degradation, predicted ICs were compared to the experimentally determined ones. Modeled ICs were obtained using a mass balance model (MBM) from Bittner et al.^3^ Briefly, the MBM assumes that after 96 h, the IC of the chemical has reached a steady state and the chemical is not metabolized. Furthermore, it is assumed that in equilibrium, the external aqueous concentration is equal to the internal aqueous concentration. Simplifying, it was assumed that neutral and ionic chemicals show similar membrane permeabilities. As experiments were performed at a constant pH of 7.4, similar to the internal pH, no ion-trapping was considered, which effectively means that the internal aqueous concentration (IC_w_) equals to the external aqueous concentration (C_w_) (eq 2).
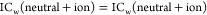
2Based on the IC_w_, the total internal concentration (IC) can be predicted. For hydrophilic chemicals, the IC is assumed to be equal to the IC_w_. However, the distribution into other compartments must be considered for more hydrophobic compounds. Chemicals can interact strongly with proteins and lipids.^24^ All other compartments were assumed to be not adsorbing. Consequently, the partition into these compartments was modeled by consideration of the respective chemical specific dissociation ratios and measured body mass parameters^10^ of the embryos including volume fractions (Vf) of lipids (Vf_lip_), proteins (Vf_protein_) and aqueous phase (Vf_w_). The detailed derivation of the BCF_MBM_ (eq 3) is provided in the SI (section S2).
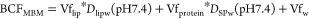
3

## 3 Results & Discussion

### 3.1 Comparison of Experimental and Predicted ICs

BCFs were experimentally determined for 63 chemicals with a broad range of hydrophobicity (log*D*_lipw_ −1.0−6.8), molecular weight (68−622 Da) and p*K*_a_-values (2.5–15.6) (i.e., SI, Excel File, sheet S1). Measured BCFs were compared to physicochemical and structural properties: lo*gD*_lipw_, number of heteroatoms, molecular weights and double bond equivalents (SI, **Figure S4**). As expected, ICs in the ZFE correlated with the hydrophobicity, i.e., log*D*_lipw_, of the chemicals (SI, **Figure S4 A**). There was no significant trend between observed BCFs to number of heteroatoms, number of double bonds, or molecular weight (SI, **Figure S4 B, C, D**).

**Figure 1:**
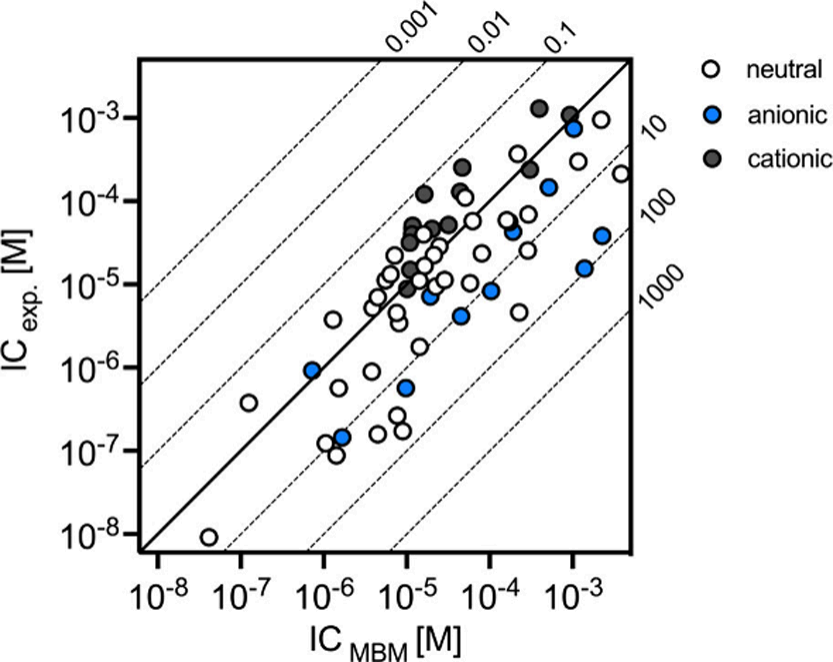
Comparison of MBM predicted (*IC*_,MBM_) to experimentally determined internal concentrations (I*C*_exp._) of 63 test chemicals related to the volume of the ZFE of 253 nL at a life stage of 96 hpf.^10^ Solid line does not represent a linear regression, but a 1:1 agreement of MBM predicted and experimental BCF. Dotted lines represent a factor of the orders of magnitude of deviation between MBM and experimental data. Exposure concentrations were at least by factor 10 below the observed LC_50_ values. Analytically determined exposure concentrations are provided in the SI (Excel file, sheet S1).

The experimentally determined ICs were compared with the modeled ICs (Figure 1). Predicted ICs deviated by no more than 1 order of magnitude from the measured IC for 79% of the chemicals (Figure 1). Very low deviations (factor 0.8 to 1.4) were found for 2-ethyl-4-methyl-1H-imidazole, valproic acid, tamoxifen, 1-vinylimidazole, mebendazole, metyrapone, and sorafenib (i.e., SI, Excel File, sheet S1). The good agreement of predicted to experimental internal concentrations confirms that the uptake of most chemicals is mainly driven by their partitioning and can therefore be predicted by their physicochemical properties describing hydrophobicity.^12^ Thus, a simple partition model can be used as a first estimate of the internal concentration of many chemicals in the ZFE. However, for 13 of the 63 study chemicals (22%) the predicted internal concentration was more than a factor of 10 higher than the measured ones, highlighting the need to experimentally verify predicted internal concentrations (Table 1). The ionic state of a chemical is crucial for its partition between the water and the embryo, since neutral species are absorbed to a higher extent compared to anions and cations.^3,18^ This is also reflected by LC_50_ of baseline toxicants as the effect concentrations are lower at pH values that favor neutral species^18^ or MBM prediction. This issue is considered by the use of log *D*_lipw_ (pH 7.4). The *D*_lipw_ represents the *K*_lipw_ of both the neutral and the charged species by considering the proportion of each species. However, this *D*_lipw_ is in some cases, particularly for anionic species, not accurate given that the predicted *K*_lipw_ deviate from measured.^14^

The predicted BCF of *N*,*N*′-methylene-bis-acrylamide (NMBA) was 28-times higher than the experimental one (Table 1), which can be related to its high reactivity to biomolecules such as glutathione.^25^ For 15 of the 63 chemicals, ICs in the ZFE were reported in previous studies, and for 9 chemicals, ICs were reported only in adult zebrafish or other fish species (e.g., rainbow trout). The BCFs of these 24 chemicals reported in literature (SI, Excel file, sheet S1, references are provided in sheet S1) agreed well the present experimental BCFs, generally within 1 order of magnitude (Figure S7). For 48 of the 63 chemicals (76%), ICs in ZFE were not reported in the literature. The previously reported BCF of colchicine had a value of 0.07,^12^ 1 order of magnitude lower than in the present study (BCF = 1.5 ± 0.4). Brox et al.^12^ demonstrated a time-dependent decrease of internal concentration of colchicine in the ZFE, which could explain the deviation between predicted and experimental BCF in the present study. For example, the experimental BCF of 1.89 ± 0.26 (pH 7.4) of diclofenac in this study is similar to the BCF of 2.46 ± 0.52 (pH 7.0)^3^ reported in literature. Chlorpyriphos had 13 times lower BCFs in the ZFE compared to the literature (SI, Figure S7). This could be related to different experimental conditions which might lead to hydrolysis. These specific examples from previous studies highlight the need of comprehensive studies on uptake and biotransformation of the parent compounds to interpret the deviations between observed and predicted internal concentration as provided in the present study.

**Table 1:** Predicted and experimentally determined BCFs of chemicals that deviate from the mass-balance model by more than a factor of 10. Information on charge, recovery, and detection of transformation products (TPs) is included for each chemical. BCF_Ex._ are related to a 96 h exposure at one sublethal concentration. NMBA − *N*,*N*′-methylene-bis-acrylamide, N.A. − not available; α_neutral_ - neutral fraction at pH 7.4; C_w_ − measured exposure concentration

Chemical	log*D*_lipw_ (pH 7.4)	α_neutral_ (pH 7.4)	Charge a (pH 7.4)	C_w_ [μM]	BCF_MBM_	BCF_Ex._	BCF_MBM_/BC*F*_exp._	BCF_Lit._	Affinity to efflux transporter	TPs found in ZFEs
NMBA	3.20	100%	neutral	4.9	0.91	0.032 ± 0.031	28.4	N.A.	N.A.	this study
Atorvastatin	4.60	0.2%	negative	3.7	608	10.3 ± 8.6	59.2	N.A.	N.A.	this study
Niflumic acid	3.60	0.3%	negative	3.6	62.9	1.3 ± 0.2	49.2	N.A.	N.A.	this study
Colchicine	2.41	100%	neutral	14.4	20.0	1.5 ± 0.4	13.7	0.07^12^	yes^12,26^	Brox et al. 2016.^12^
Carbamazepine	3.10	100%	neutral	0.1	22.4	1.4 ± 0.8	16.1	2.3^10^	yes^27^	Halbach et al. 2020.^10^
Methotrexate	−1.00	0%	double negative	1.8	0.9	0.08 ± 0.02	11.5	N.A.	N.A.	N.A.
Diclofenac	2.65	0.05%	negative	3.3	31.8	1.9 ± 0.3	12.5	2.5^3^	yes^28^	Nawaji et al. 2020.^29^
Ethoprophos	3.71	100%	neutral	42.7	89.8	5.0 ± 1.2	18.0	N.A.	N.A.	this study
Pirinixic acid	3.20	0.4%	negative	51.8	26.8	0.30 ± 0.32	89.7	N.A.	yes	this study
Chlorpyriphos oxon	4.30	100%	neutral	0.03	299	5.8 ± 4.1	52	N.A.	N.A.	Nayak et al. 2023.^30^
Fipronil	4.16	100%	neutral	0.03	240	8.3 ± 0.6	29.0	N.A.	N.A.	Chao et al., 2019.^31^
Triatricol	4.65	0.1%	negative	0.014	690	40 ± 21	17.2	N.A.	N.A.	N.A.
Iopanoic acid	4.14	100%	negative	0.21	214	20 ± 4	10.9	N.A.	N.A.	N.A.

### 3.2 Importance of Biotransformation for the IC

**Figure 2:**
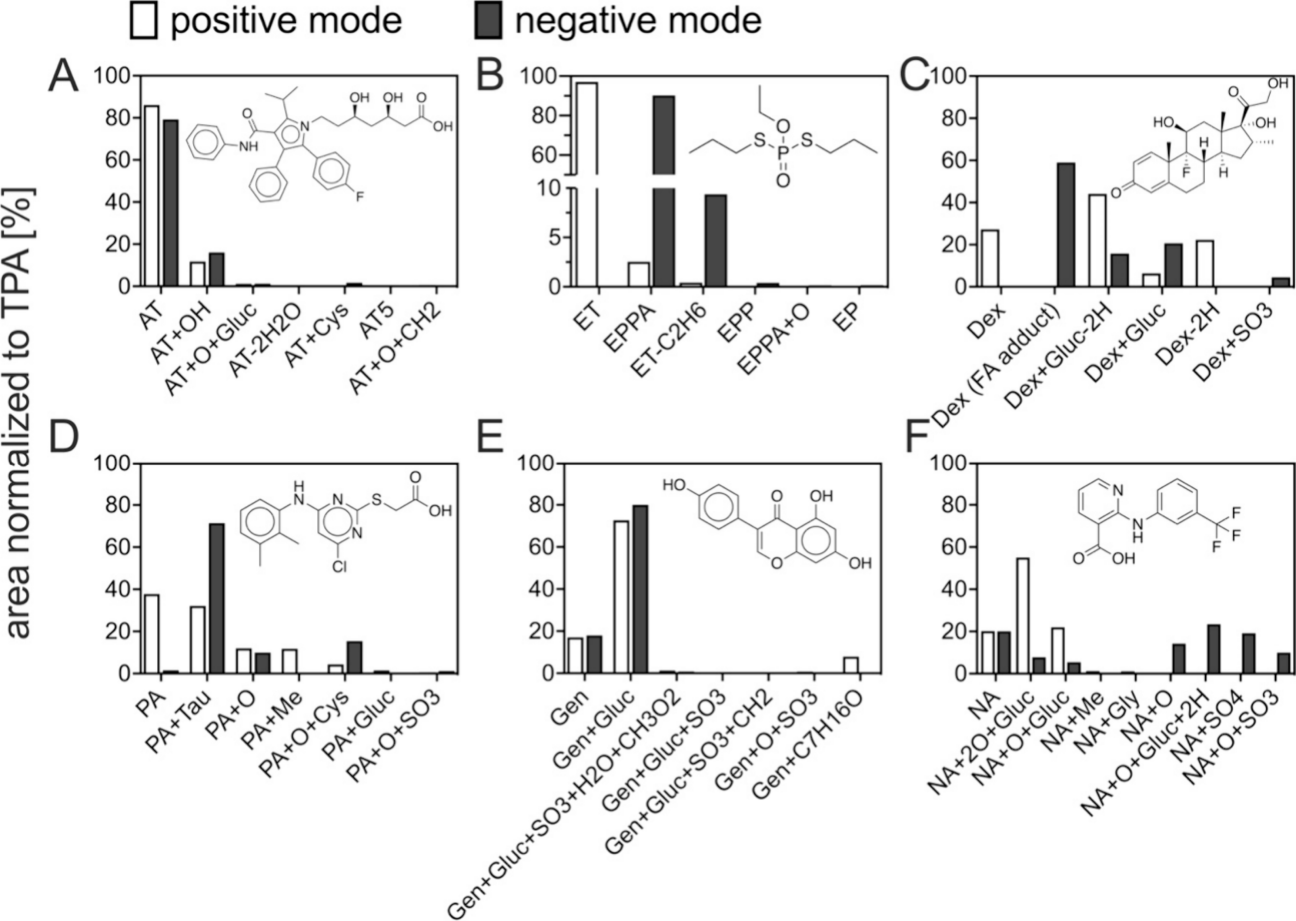
Distribution of TPs of (**A)** atorvastatin (AT), (**B)** ethoprophos (ET), (**C**) dexamethasone (Dex), (**D**) pirinixic acid (PA), (**E**) genistein (Gen) and (**F**) niflumic acid (NA) in extracts of exposed ZFEs after 96 h. Peak areas of the test chemicals and their TPs were normalized to the total peak area (TPA). MS data were recorded in positive and negative ionization mode. Exposure was started at 4 ± 1 hpf. Total peak area is related to the sum of all of the TPs detected and their corresponding parent al compounds. EPPA − O-ethyl S-propyl phosphorothioate, EP = ethylphosphate, EPP - O-ethyl O-methyl S-propyl phosphorothioate, FA = formic acid, Gluc = glucuronide, Gly = glycine, Tau = taurine, Cys = cysteine, Me−methyl.

Biotransformation could bias the mode of action classification of the chemical, as the biologically available dose of the parental compound would be overestimated by a mass balance model. In other words, the hazard and risk associated with potential specific modes of action could be underestimated. Furthermore, metabolism becomes important when it results in the formation of active metabolites. In order to analyze the role of metabolites in more detail, six of the study chemicals were selected for a detailed investigation of their biotransformation in the ZFE (Figure 2). Atorvastatin, pirinixic acid, niflumic acid, and ethoprophos had internal concentration 10 to 90-fold lower than predicted IC at 96 hpf, which makes it likely that metabolism decreased the parent concentration. Genistein and dexamethasone were selected for TP analysis, because biological processes, e.g., biotransformation, seem to influence their classification when ICs are considered from TR_ext._ < 10 to TR_int._ > 10.

Nontarget- and suspect screening were performed to detect and tentatively identify TPs of the six study compounds in ZFE (i.e., SI, section S6.2). In total, eight tentative TPs of niflumic acid, five of ethoprophos, six of genistein, six of pirinixic acid, four of dexamethasone, and six of atorvastatin were detected in extracts of exposed ZFEs (i.e., SI, Excel file, sheets S4−S9). These 35 potential TPs are mainly formed from phase I hydroxylation, oxidation and reduction followed by phase II glucuronidation, glycine and Cys conjugation, sulfation, acetylation and methylation. Most of the predicted and previously reported TPs (see SI, Excel File, Sheets S10 and S11) were found in the exposed ZFE extracts. Only for genistein, TP data for aquatic species (rainbow trout, white sturgeon, atlantic salmon) were reported in the literature.^32^ For atorvastatin, pirinixic acid, niflumic acid, dexamethasone, and ethoprophos, TPs are reported for the first time in an aquatic species in this study.

The TPs could only be semiquantified using their total peak areas (TPA) due to the limited availability of reference standards. Analytical standards would be required for accurate quantification (Figure 2). Semiquantitative data of TPs are at best proxies of the ICs of TPs. Although the ICs of the parental compounds were quantified, only semiquantitative data could be used for assessment of the relationship between TPs and parents in order to compare their relative importance in the ZFE. Peak response areas from the HRMS can be strongly influenced by matrix effects and chemical structure (ionization efficiency and fragmentation).^33^ For example, the taurine metabolite of pirinixic acid exhibited 32% of the TPA (including peak areas of parental compounds and their TPs) in positive ionization mode while it was 71% in negative mode. This reflects the enhanced ionization of the sulfonate containing taurine metabolite in the negative mode. Another example is dexamethasone, showing 27% TPA in positive mode and 59% TPA in negative mode. However, dexamethasone was only detected as formate adduct in negative mode, so the response factor of [M+CHO_2_]^−^ might be substantially higher compared to the [M-H]^−^ ion. Ethoprophos could not be detected in negative mode but in positive mode. TPs of ethoprophos showed a significantly higher ionization efficiency in negative compared to positive mode (Figure 2B). For niflumic acid, the relative TPA contribution of the parental compound is similar, but the importance of its TPs varies in positive and negative modes (Figure 2F). In positive ionization mode, glucuronides seem to be more relevant, while in negative mode NA+O, NA+Gluc+O+2H and sulfates were ionized more efficiently. For atorvastatin and genistein, peaks of parental compounds and TPs show similar areas in positive and negative modes (Figure 2**A,E**). This demonstrates that both ionization modes must be considered for semiquantitative assessments.

In the case of atorvastatin, biotransformation does not seem to be quantitatively significant. The most important ones from five detected TPs, hydroxylated and glucuronidated atorvastatin, account for 22 and 1.6% of the TPA (Figure 2A). Therefore, it may be concluded that biotransformation of atorvastatin is not a likely explanation for the deviation of the modeled IC from the experimental IC (Figure 2A). The most intense TP of ethoprophos, O-ethyl S-propyl phosphonothioate (EPPA) reaches only 2% TPA in positive mode, but its signal intensity increases by 1 order of magnitude in negative ionization mode (Figure 2B). Thus, the TPA contribution of EPPA in negative mode is similar to the one of ethoprophos observed in positive mode. ET-C_2_H_6_ shows a 10% TPA contribution in negative mode.

For dexamethasone, pirinixic acid, genistein, and niflumic acid, biotransformation appears to be quantitatively more relevant (Figure 2**C−F**). More than 60% of pirinixic acid, 73% of dexamethasone, 83% of genistein, and 85% of niflumic acid were biotransformed after 96 h based on the TPA data (Figure 2**C−F**). This may indicate a relatively high biotransformation to uptake ratio, resulting in an IC of the parental compounds that are by a factor of 5 to 90 lower than predicted. The semiquantitative data from this study indicate that quantitative data on biotransformation would improve the prediction performance of the MBM. However, since only semiquantitative data were used for these assessments, only total quantification of parental compounds and their TPs would lead to a definite conclusion.

### 3.3 Potential role of Transport for ICs

It was already demonstrated that ICs of chemicals can be modulated by, e.g., the ATP-binding cassette (ABC) efflux transporter^34^ and solute carrier transporter family including organic anion transporting polypeptides (OATP).^28^ This may explain lower than predicted concentrations for chemicals for which biotransformation was not found to be significant. Indeed, for three of the chemicals listed in Table 1 (carbamazepine,^27^ diclofenac^28^ and colchicine^26^), literature data suggest enhanced efflux activity (Table 1). For example, it was reported that diclofenac (at pH = 7.4) is a substrate of the Oatp1d1 transporter in the ZFE.^28,35^ Halbach et al. demonstrated a significant decrease of the accumulation rate of diclofenac in HEK cells when they were transfected with zebrafish Oatp1d1, indicating an efflux transporter activity in this case.^28^ For the remaining chemicals, no data have been published about their efflux activities. Therefore, experimental evidence of cellular efflux transport for these compounds would be of interest for future studies. However, for atorvastatin a competitive inhibition of the zebrafish Oatp2b1 uptake transporter was reported.^36^ Due to the structural similaritiy to Oat1d1, a bidirectional activity of Oat2b1 in zebrafish is possible similar to what was shown for Oatp1d1.^28^ Future research would be important to characterize the general bidirectional behavior of the Oatp family and their modulation of ICs of chemicals. To draw a definite conclusion on the impact of efflux on the ICs of such chemicals, data are required on the impact of inhibition of the transporter protein to the IC of the respective chemical in the ZFE.

### 3.4 Importance of Uptake Kinetics for the IC

**Figure 3:**
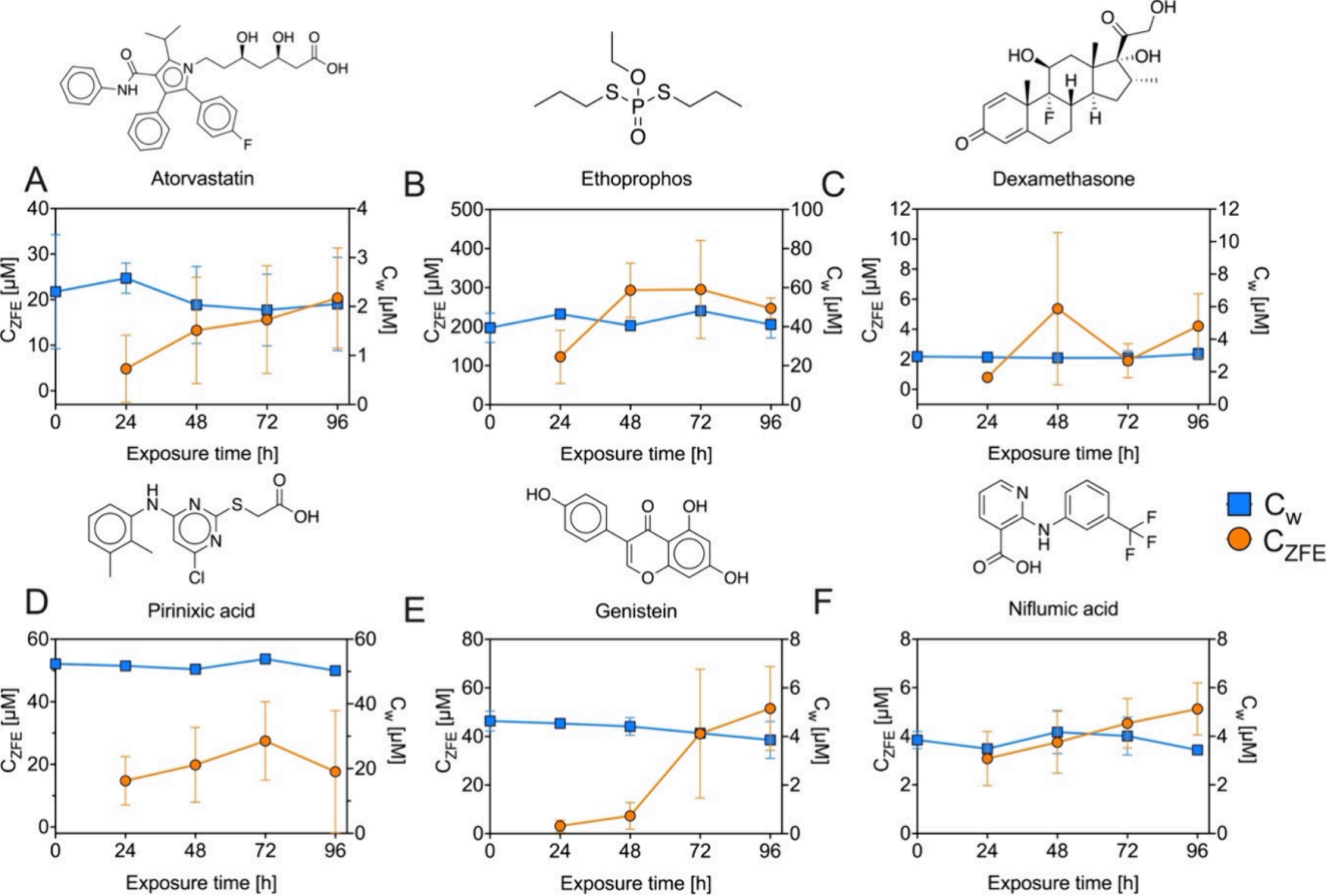
Time course of internal (C_ZFE_) and external concentrations (C_w_) of (**A**) atorvastatin (*n* = 5), (**B**) ethoprophos (*n* = 5), (**C)** dexamethasone (*n* = 5), (D) pirinixic acid (*n* = 5), (**E**) genistein (*n* = 6) and (F) niflumic acid (*n* = 5) showing 5 to 90-fold lower C_ZFE_ than predicted by MBM. The exposure experiments were started at 4 ± 1 hpf.

Biotransformation^12,17^ or active efflux by transporters^28,34^ will result in lower steady-state ICs and therefore smaller BCFs, but if uptake kinetics are slow, steady state might not be reached within 96 h, which also results in smaller experimental BCFs. To further investigate the cause of the discrepancies between measured and predicted BCF, the uptake kinetics of six chemicals were investigated (Figure 3). Aqueous concentrations were stable for all test chemicals over a 96 h exposure (Figure 3), so there was no significant loss due to sorption or chemical degradation. The selected study compounds showed different time courses of ICs in the ZFE (Figure 3).

Uptake of atorvastatin appears not to have reached steady state within 96h (Figure 3**A**). Ethoprophos was absorbed fast and reached a stable IC between 48 and 72 h, showing high data variability (Figure 3**B**). The uptake kinetics of dexamethasone display no clear trend with a high standard deviation at 48 h and a statistically not significant (*p* = 0.13) decrease from 48h to 72h (Figure 3C). The time course of IC of pirinixic acid shows a trend similar to that of ethoprophos, albeit with strong data variability (Figure 3D). Uptake of genistein was initially slow but increased markedly between 48 and 72 h without reaching equilibrium within 96 h (Figure 3E). Also the time course of the IC of niflumic acid in the ZFE does not indicate that a steady-state concentration was reached (Figure 3F). As atorvastatin, genistein and niflumic acid are negatively charged at the ambient pH of 7.4 and their uptake kinetics does not indicate a steady state of ICs, the slow uptake could contribute to the overprediction of their BCFs. A similar uptake trend as for genistein was observed for the negatively charged perfluorooctanesulfonic acid in the ZFE.^37^ Biotransformation or enhanced efflux activity may led to unstable uptake trends as demonstrated for dexamethasone, slow uptake without reaching steady-state concentrations in 96 h as shown for atorvastatin, genistein, and niflumic acid, or to potential lower steady-state concentrations like for ethoprophos and pirinixic acid.

### 3.5 Experimental ICs Improve Specificity Classification of Chemicals

**Figure 4:**
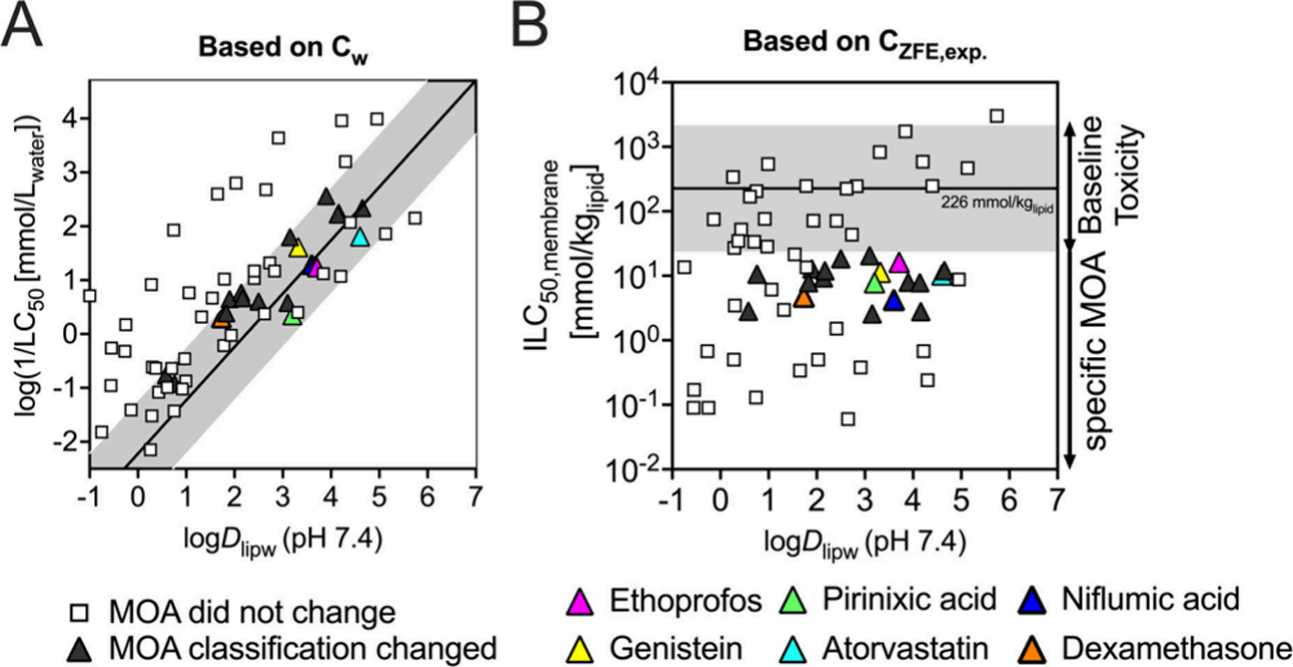
**A** Correlation between the experimental aqueous log(1/LC_50_) and log*D*_lipw_ at pH 7.4. The solid line indicates the generic QSAR for baseline toxicity for ionizable chemicals according to Klüver et al. (2019).^38^ The gray area around the QSAR represents a toxic ratio (TR) range between 0.1 and 10. **B** Internal membrane concentrations (ILC_50,membrane_) based on the measured BCF of 63 test chemicals. The critical membrane concentration ILC_50,baseline_ of 226 ± 178 mmol/kg_lipid_ was adapted from Klüver et al. (2016).^5^ Chemicals around this ILC_50,baseline_ (gray area) with deviation by a factor of 10 are considered as baseline toxicants. Chemicals outside this range are classified to exhibit a specific MOA.

The nominal LC_50_ values of the 63 study chemicals varied between 0.1 μM for sorafenib to 140 mM for *N*,*N*′-bis(2-hydroxyethyl)-2-nitro-p-phenylenediamine highlighting the broad range of toxicities of the test compounds by 6 orders of magnitude. In Figure 4A, the observed half-maximal lethality log 1/LC_50_ is plotted against the hydrophobicity (log*D*_lipw_) of the study chemicals. Additionally, the observed LC_50_ data were related to a baseline toxicity QSAR according to Klüver et al.^38^ to visualize the toxic ratios (TRs) of the chemicals (Figure 4A). The QSAR from Klüver et al. is not defined for chemicals with log*D*_lipw_ < 0 as there are no experimental data.^38^ However, it was demonstrated by Huchthausen et al.^14^ that a similar QSAR for reporter gene cell lines is also valid for chemicals with log*D*_lipw_ between 0 and −1 and therefore we had included compounds with a log*D*_lipw_ as low as −1 in the calculation of TRs.

To demonstrate the importance of experimental ICs for the classification of chemicals, classification based on nominal aqueous versus experimentally determined ICs was compared. For the specificity analysis based on experimental ICs, internal membrane concentrations (ILC_50,membrane_) were used to assess whether observed toxicity is a result of baseline toxicity or specific mechanisms. The ILC_50,membrane_ were estimated using measured body mass parameter of the ZFE.^10^ Obtained ILC_50,membrane_ data were plotted against the hydrophobicity (log*D*_lipw_) and related to the critical membrane concentration of baseline toxicity ILC_50,baseline toxicity_ of 226 ± 178 mmol/kg_lipid_^5^ to visualize internal TRs (Figure 4B).

All putative baseline toxicants and specific acting chemicals were classified correctly using nominal aqueous and experimental ICs (Figure S6 and Table S9). Indeed, 19 chemicals with a TR_external_ < 10 exhibited TR_internal_ > 10 (Figure 4A) when the measured ICs were considered and hence were no longer classified as baseline toxicants (Figure 4B, Table 2). For most of the chemicals, whose experimentally determined IC deviated from the MBM by less than 1 order of magnitude, the TR_external_ and TR_internal_ led to the same MOA classification. This was not the case for dexamethasone, genistein, pirinixic acid and niflumic acid that were effectively biotransformed (Figure 2) and showed TR_internal_ > 10 while their external TR_external_ (eq 9 in the SI) are in range of baseline toxicity (Figure 4).

In the case of genistein, paracetamol, phenytoin, and clofibric acid, biotransformation seems to be more relevant. Effective biotransformation of genistein was observed in the present study (Figure 2A), while it was reported previously for phenytoin, paracetamol as well as for clofibric acid.^12,20^ A very low uptake of cyclophosphamide was reported by Brox et al. as well also explaining the discrepancies between MOA classification based on nominal and internal effect concentrations.^12^ For imazalil, naproxen, atrazine, and valproic acid the TR based classification changes as well, although their measured ICs are close to the predicted ones (Table 2). Their experimentally determined ILC_50,membrane_ values are close to the predicted ILC_50,membrane_, which are close to TR_internal_ 10 as well (Table 2). Thus, these differences are more likely due to experimental data and biological variability. Naproxen, imazalil, atrazine and valproic acid have known specific MOAs, but effect data in aquatic organisms suggest nonspecific toxicities in nontarget organisms like the ZFE indicating a lack of their molecular targets^18,38,39^ or different toxicokinetics.^3,38,40^ These results emphasize that biotransformation and uptake kinetics need to be considered in the specificity MOA assessment of chemicals. Considering ICs, 23 chemicals have been classified as baseline toxicants and 40 chemicals were classified to exhibit a specific mode of action among them mainly pesticides and pharmaceuticals.

**Table 2:** Summary of chemicals for which the MOA classification changed when ICs were considered

Chemical	TR_ext_	TR_int._	BCF_MBM_/BC*F*_exp_	ILC_50,membrane_ experimental [mmol/kg_lipid_]	ILC_50,membrane_ predicted [mmol/kg_lipid_]
Pirinixic acid	0.3	29	89.7	8	702
atorvastatin	0.3	22	59.2	10	612
Niflumic acid	0.9	54	49.2	4	208
Fipronil	2.3	81	29.0	3	81
Ethoprophos	0.6	14	18.0	16	294
Tiratricol	0.9	19	17.1	12	207
Carbamazepine	0.5	11	16.1	21	332
Iopanoic acid	2.2	29	10.8	8	85
Triadimenol	8.0	88	8.6	3	22
Paracetamol	7.4	80	8.0	3	23
dexamethasone	6.4	48	5.6	5	27
Cyclophosphamide	3.5	21	4.5	11	48
Genistein	3.5	20	4.5	11	51
Phenytoin	2.2	12	4.2	18	78
clofibric acid	6.2	29	3.5	8	28
Imazalil	8.3	28	2.7	8	22
Naproxen	5.5	19	2.7	12	32
atrazine (ATZ)	7.4	24	2.5	9	24
valproic acid	10	18	1.4	13	18

## 4 Implications

The results from the present study provide knowledge on the biotransformation capacity of the zebrafish at early life stages, since 35 tentative TPs of compounds are reported for the first time in the ZFE and are relevant for future toxicity studies. With the knowledge on their IC in the ZFE, 40 chemicals were classified to exhibit a specific mode of action, and 23 chemicals were classified as baseline toxicants. This study confirms earlier findings that the MBM appears to be a good first estimation for the IC of xenobiotics in the ZFE unless they are metabolized or have slow uptake kinetics. Chemicals that can be metabolized would require a toxicokinetic model where the not only the uptake and elimination kinetics of the parent can be implemented but also the formation of transformation products and their elimination.^41^ This is not possible with the presently available experimental data set. For chemicals aligning to the MBM model used in the present study, typically, the extent of uptake is driven by the chemical’s hydrophobicity expressed by log*D*_lipw_, which was confirmed for 79% of the 63 study chemicals. For the remainder, the comparison of MBM prediction and experimental determination of ICs led to a better understanding of the role of biotransformation and efflux transporters. These results highlight the relevance of experimental toxicokinetic data because the internal effective dose can be overestimated when biotransformation or efflux is not considered, which can bias the classification of chemicals and underestimate the chemical toxicity. To improve the performance of the MBM, quantitative TP data using analytical standards are required. Nevertheless, internal concentration screening and semiquantitative assessment of TPs could be included in routine assessment of zebrafish embryo toxicity tests to improve identification of nonbaseline toxicity mode of actions. In the case of deviations from the MBM, detailed studies could aim at a more quantitative assessment including potentially also the assessment of the role of efflux transporter. The latter could for instance, be studied using gene knockouts of the respective transporter genes.^42^ The results of this study also highlight the suitability of the ZFE model for toxicokinetic assessments as a cost-effective and ethically acceptable alternative to animal testing.

## Supporting Information Available

Preparation of standard and exposure solutions; devices and software; prediction of internal concentrations and toxic ratio analysis; Quality assurance and quality control; Instrumental parameters; nontarget screening of TPs; MS/MS spectra of TPs (PDF)
Physicochemical properties of test chemicals and mass balance model (S1). Experimental raw data of external and internal concentrations (S2). MS Data for transformation products (S3−S8) (XLSX)

## Abbreviations:

BCF: bioconcentration factor
Cys: Cysteine
FET: Fish embryo toxicity test
Gluc: glucuronide
GSH: glutathione
GSH: glutathione
hpf: hours post fertilization
IC_50_: half-maximal inhibitory concentration
ILC_50_: median internal lethal concentration of a substance that is expected to cause death in 50% of the exposed animals
LC_50_: median lethal concentration of a substance that is expected to cause death in 50% of the exposed animals
MBM: mass balance model
PCA: principal component analysis
QSAR: quantitative structure−activity relationship
RT: room temperature
Tau: Taurine
TK: toxicokinetics
TP: transformation product
TPA: total peak area
TR: toxic ratio
UPLC-QTOF-MS: ultraperformance-liquid-chromatography quadrupole-time-of-flight mass spectrometry
ZFE: zebrafish embryo
