# Supplementary material for: Impact of Biotransformation on Internal Concentrations and Specificity Classification of Organic Chemicals in the Zebrafish Embryo (Danio rerio)
Source: Environ Sci Technol. 2024 Sep 24;58(40):17898–907. doi: 10.1021/acs.est.4c04156 (PMC11465767; doi:10.1021/acs.est.4c04156)
Supplement: Supplementary file 1 — es4c04156_si_001.pdf [file es4c04156_si_001.pdf]

Supporting information 1 for

Impact of Biotransformation on Internal Concentrations and Specificity Classification of Organic Chemicals in the Zebrafish Embryo (Danio rerio)

Nico Grasse[a], Riccardo Massei[b], Bettina Seiwert[a], Stefan Scholz[b], Beate I. Escher[c,d], Thorsten Reemtsma[a,e], Qiuguo Fu[a*]

[a] Department of Environmental Analytical Chemistry, Helmholtz-Centre for Environmental Research – UFZ, Permoserstrasse 15, 04318 Leipzig, Germany.
[b] Department of Ecotoxicology, Helmholtz-Centre for Environmental Research – UFZ, Permoserstrasse 15, 04318 Leipzig, Germany.
[c] Department of Cell Toxicology, Helmholtz-Centre for Environmental Research – UFZ, Permoserstrasse 15, 04318 Leipzig, Germany.
[d] Environmental Toxicology, Department of Geosciences, Eberhard Karls University Tübingen, Schnarrenbergstr. 94-96, DE-72076 Tübingen, Germany
[e] Institute for Analytical Chemistry, University of Leipzig, Linnestrasse 3, 04103 Leipzig, Germany.

*Corresponding to:
Helmholtz-Centre for Environmental Research – UFZ
Department of Environmental Analytical Chemistry
Permoserstrasse 15, 04318 Leipzig, Germany
Dr. Qiuguo Fu
E-mail address: qiuguo.fu@ufz.de

Number of pages: 84
Number of figures: 7
Number of tables: 9
Number of equations: 16

Contents

1. Materials and Methods 4

1.1 Solvents, buffers and additives 4

1.2 Fish husbandry 4

1.3 Preparation of ISO water with HEPES buffer 5

1.4 Preparation of chemical exposure media and calibration standards 5

1.5 Toxicokinetic experiments 5

1.6 Devices and Software 6

2. Prediction of internal concentrations based on mass balance model (MBM) 7

3. Internal membrane concentrations 9

4. Toxic ratio analysis 10

5. Quality assurance and quality control 10

5.1 Stability assessment 10

5.2 Contribution of adsorption to the ZFE to the detected extract concentration 11

6. Instrumental parameters 12

6.1 HPLC-MS/MS analysis 12

6.1.1 Mass spectrometric parameters 13

6.1.2 High-performance liquid chromatography 16

6.1.3 Limits of detection, limits of quantification and linear dynamic range 16

6.1.4 Matrix effects and recovery 19

6.2 UPLC-QTOF-MS 20

6.2.1 Detection of transformation products (TPs) using UPLC-QTOF-MS 20

6.2.2 Identification of TPs via suspect- and non-target-screening 21

7. Correlations of internal concentrations with physicochemical properties 22

8. Non-target screening of transformation products 23

9. Specificity analysis based on toxic ratios and correlation between external and internal concentration of atorvastatin 24

10. Extracted-ion-chromatograms, MS and MS/MS spectra of biotransformation products 26

10.1 TPs of Pirinixic acid 26

10.2 TPs of atorvastatin 36

10.3 TPs of Genistein ... 46
10.4 TPs of Ethoprophos ... 56
10.5 TPs of Dexamethasone ... 64
10.6 TPs of Niflumic acid ... 71
11. References ... 83

Figures

Figure S1: Assessment of recoveries of test compounds in and **A** 96 well plates (plastic) and **B** HPLC glass vials ... 11
Figure S2: Influence of adsorption to the ZFE on the detected internal concentration ... 12
Figure S3: **A** Method recovery (n = 3; 25 ng/mL standard mix) and **B** matrix effects (n = 3, 20 ng/mL standard mix) of 63 test compounds in extracts of eight ZFEs ... 20
Figure S4. Dependency of observed bioconcentrations factors ($BCF_{experimental}$) to **A** hydrophobicity expressed as liposome-water distribution ratio ($\log D_{lipw}$), **B** the number of heteroatoms of all 63 chemicals, **C** the molecular weight and **D** equivalents of double bonds ... 22
Figure S5: Feature analysis of analyzed extracts of ZFEs exposed to the respective chemical for 96 h and non-exposed ZFEs ... 23
Figure S6: Comparison nominal $TR_{external}$ values from the FET using experimental aqueous LC_{50} data to $TR_{internal}$ based on experimental internal ILC_{50} data ... 25
Figure S7: Correlation between external and internal concentrations of atorvastatin ... 25

Tables

Table S1: Solvents, buffers and additives. ... 4
Table S2: Composition of ISO water. ... 5
Table S3: Summary of devices used in this study with model and supplier information. ... 6
Table S4: Software with information about supplier and version. ... 6
Table S5: Compound-specific parameter for MRM transitions of study compounds including retention times. ... 13
Table S6: LOD, LOQ and LDR data for each study chemicals. ... 17
Table S7: Instrumental parameters of UPLC-qTOF-MS method for transformation product analysis. ... 21
Table S8: Parameter of MarkerLynx method used for detection of transformation products. 22
Table S9: Study chemicals with known mode of action (MOA) with their predicted and experimental bioconcentration factors (BCF_{MBM} and $BCF_{Exp.}$), half-maximal lethal concentrations (LC_{50}), baseline toxicities ($LC_{50,baseline}$), critical membrane concentrations ($ILC_{50,membrane}$) and toxic ratios (TRs) ... 24

1. Materials and Methods

1.1 Solvents, buffers and additives

Table S1: Solvents, buffers and additives.

Substance	CAS number	Supplier	Quality
Ammonium formate	540-69-2	Sigma-Aldrich (Munich, Germany)	>99 %
HEPES	7365-45-9	Carl Roth GmbH + Co. KG (Karlsruhe, Germany)	>99.5 %
Methanol	67-56-1	Biosolve (Valkenswaard, Netherlands).	> 99 %
Formic acid	64-18-6	Biosolve (Valkenswaard, Netherlands).	> 98 %

1.2 Fish husbandry

Fish husbandry was conducted according to Grasse et al. (2023).[1] Adult wild-type zebrafish (strain UFZ-OBI, Leipzig) have been bred for over 13 generations. Fish were held in 14-L tanks with 35 fish each with a female-to-male ratio of 1:1. At 1 hour post fertilization (hpf) fertilized embryos were selected by checking for four-cell stage embryos according to Kimmel (1995).[2] The exposure experiments were started with zebrafish embryos (ZFEs) at 4 hpf.

The light–dark rhythm was 14:10 h and the water temperature was kept constant at 26 ± 1 °C. Water quality parameters (pH, water hardness, conductivity, nitrate, nitrite, ammonia, oxygen saturation) were determined biweekly. Light was used to induce spawning, and eggs were collected. The eggs were collected in glass trays covered with 3 mm mesh and artificial plants within 30 min after spawning and cleaned sequentially with ISO standard dilution water according to ISO 7346-3. All the zebrafish were cultured and kept under German and European animal welfare standards and were approved by the Saxon State Government, Leipzig State Directorate, Germany (Aktenzeichen 75-9185.64).

1.3 Preparation of ISO water with HEPES buffer

A solution of 10 mM (4-(2-hydroxyethyl)-1-piperazineethanesulfonic acid) (HEPES) with a pH of 7.4 in ISO water was used for the exposure experiments. The ISO water was prepared from four different salts in accordance with DIN EN ISO 7346-3 (1997).

Table S2: Composition of ISO water.

Stock solution	Ingredients	Concentration [g/L]
1	$CaCl_2 \cdot 2\ H_2O$	11.760
2	$MgSO_4 \cdot 7\ H_2O$	4.932
3	$NaHCO_3$	2.52
4	KCl	0.22

1.4 Preparation of chemical exposure media and calibration standards

The chemicals for ZFE exposure experiments were solubilized in a medium defined by the International Organization for Standardization (ISO) containing 10 mM 4-(2-hydroxyethyl)-1-piperazineethanesulfonic acid (HEPES) at pH 7.4. The composition of ISO water is provided in **Table S2**. The 1 mg/mL stock solutions of the chemicals were prepared in methanol (MeOH) and stored at – 20°C. MeOH stock solutions were used for both ZFE exposure and as analytical standards. For calibration, a standard mixture of test chemicals was diluted 1:100 in MeOH to obtain a working solution of 10 µg/mL. Calibration standards were prepared from this solution with MeOH in the range of 0.1 ng/mL and 500 ng/mL. MeOH extracts from 8 non-exposed ZFE at 96 hpf were used to prepare method-matched calibration standards. These standards were diluted 1:1 (v/v) with ultrapure water to obtain the final calibration concentrations from 0.05 – 250 ng/mL.

1.5 Toxicokinetic experiments

A standard exposure concentration of 1.0 mg/L was used for 36 of 63 test compounds below their LC_{50}. For 17 particularly toxic chemicals, concentrations between 0.1 – 0.01 mg/L were selected for exposure. For 10 chemicals, a 10 – 1000 mg/L exposure was conducted due to the lower analytical sensitivity of the respective compound. The nominal and measured external concentrations of each chemical can be found in the Excel File (sheet S2). The MeOH stock solutions of the chemicals were diluted in ISO water to the corresponding nominal concentration with a total MeOH content of 0.1 % (v/v). For polar chemicals with $\log D_{lipw} < 3.0$ at pH 7.4 the exposure was conducted in 96-well plates. The ZFE exposure with hydrophobic chemicals ($\log D_{lipw} > 3.0$, pH = 7.4) was performed in crystallization dishes (7.5 cm diameter) to avoid adsorption of the chemicals to the plastic plates, with a proportion of one embryo per 0.4 mL exposure medium. For the screening of internal concentrations, the exposure was conducted for 96 h at 26 ± 1 °C. For time-resolved uptake experiments, samples of 8 exposed

ZFE were collected after 24 h, 48 h, 72 h and 96 h. As a negative control, fertilized eggs were exposed to ISO water containing 0.1 % MeOH without any test chemicals to check any possibilities of chemical contamination during the whole process. Dead ZFEs were discarded daily to avoid fungal growth. Exposure medium was collected at every sampling time point to confirm concentrations for test chemicals. Medium samples were collected every 24 h and stored at -20 °C until HPLC-MS/MS analysis. For the internal concentration screening of 63 chemicals, at least two technical replicates were analyzed per chemical (n = 2). For uptake experiments, three technical replicates were prepared (n = 3) per experiment. Uptake experiments were performed twice resulting in at least five replicates (n = 5). Internal concentration data are presented as mean bioconcentration factors (BCF) considering the volume of the ZFE at the respective life stage.

1.6 Devices and Software

Table S3: Summary of devices used in this study with model and supplier information.

Device	Supplier, model
Analytical Balance	Mettler, PM4800
Incubator	Heraeus Vötsch, Bioline VB 1514
Centrifuge	VWR, Galaxy 14 D
Ultrasonic bath	Emerson, Branson 5200
Vortexer	Heidolph REAX 2000
pH-Meter	Inolab /WTW; Knick, pH-Meter 765
Light microscope	Olympus, SZX2-ILLT
MilliQ Water System	Merck, Direct 8

Table S4: Software with information about supplier and version.

Software	Supplier, version
MassLynx	Waters, 4.1
TargetLynx	Waters, 4.1
MarkerLynx	Waters, 4.1
Analyst	AB Sciex, 1.6.2
MulitQuant	AB Sciex, 3.0.3
GraphPad Prism	10.0.2 (171)

2. Prediction of internal concentrations based on mass balance model (MBM)

Internal concentrations and bioconcentration factors (BCF) were estimated using the MBM from Bittner et al.[3] Simplifying, it was assumed that neutral and ionic chemicals show similar membrane permeability. As experiments were performed at a constant pH of 7.4, similar to the internal pH, no ion-trapping was considered, which effectively means that the internal aqueous concentration ($C_{ZFE,w}$) equals to the external aqueous concentration (C_w) (eq. 1).

$$C_w(\text{neutral+ion}) = C_{ZFE,w}(\text{neutral+ion}) \quad (1)$$

Based on the $C_{ZFE,w}$, the total internal concentration (C_{ZFE}), meaning the entire concentration of the chemical in the ZFE, can be predicted. For hydrophilic chemicals, the C_{ZFE} is assumed to be equal to the $C_{ZFE,w}$. However, the distribution into other compartments must be considered for more hydrophobic compounds. Chemicals can interact particularly strongly with proteins and lipids.[4] Simplifying, all other compartments were assumed to be not adsorbing. Descriptors for the distribution of the chemicals into these phases are ion-corrected lipid and structural protein distribution ratios ($\log D_{lipw}$ and $\log D_{SPw}$), which were either predicted or determined experimentally (Excel file, sheet S1). The distribution ratios consider the dissociation of the chemicals at the ambient pH by taking into account the proportion of the neutral and ionic species in the solution. The fraction of the neutral species can be expressed by the Henderson-Hasselbalch equation distinguishing between monoprotic acids (eq. 2) and bases (eq. 3) :

$$\alpha_{\text{neutral, acid}}[\%]= \frac{1}{1+10^{\text{pH-p}K_a}} \quad (2)$$

$$\alpha_{\text{neutral, base}}[\%]= \frac{1}{1+10^{\text{p}K_a\text{-pH}}} \quad (3)$$

The liposome- and structural protein distribution ratios at a specific pH can be defined as the sum of the fractions of all chemical species with their respective partition constants (K_{lipw} or K_{SPw}) (eq. 4 and 5):

$$D_{lipw}(\text{pH})=\sum_{i=1}^{n} \alpha_i {*} K_{lipw}(i) \quad (4)$$

$$D_{SPw}(pH)=\sum_{i=1}^{n}\alpha_i{*}K_{SPw}(i) \quad (5)$$

The distribution of the chemical between proteins and water in the ZFE was estimated using the distribution ratio to muscle proteins (MP) rather than to bovine serum albumin (BSA) since there is evidence, that particular organic anions exhibit a strong affinity to BSA by binding to specific binding sites of albumin.[4,5,3] Based on these parameters, the fraction of the chemical in the aqueous phase ($f_{w,\ internal}$) in the ZFE can be calculated (eq. 6), which is required to assess the whole concentration of the chemical in the ZFE (C_{ZFE}).

$$f_{w,\ internal} = \frac{1}{1+D_{lipw}(pH\ 7.4){*}\frac{Vf_{lip}}{Vf_w}+D_{SPw}(pH\ 7.4){*}\frac{Vf_{protein}}{Vf_w}} \quad (6)$$

Thereby, Vf is referred to volume fractions of lipids (Vf_{lipid}) and proteins ($Vf_{protein}$) and the internal aqueous phase (Vf_w) of the ZFE. D_{lipw} and D_{SPw} are the distribution coefficients of the test chemicals between lipids or structural proteins (SP) and water, respectively. Considering the previously defined compartments in the ZFE, the total internal concentration can be calculated according to eq. 7.

$$\begin{aligned} C_{ZFE} &= C_{ZFE,w}{*}\frac{Vf_w}{f_{w,internal}} \\ &= C_{ZFE,w}{*}Vf_w\left(1+D_{lipw}(pH\ 7.4){*}\frac{Vf_{lip}}{Vf_w}+D_{SPw}(pH\ 7.4){*}\frac{Vf_{protein}}{Vf_w}\right) \quad (7) \\ &= C_{ZFE,w}{*}\left(Vf_w+D_{lipw}(pH\ 7.4){*}Vf_{lip}+D_{SPw}(pH\ 7.4){*}Vf_{protein}\right) \end{aligned}$$

In eq. 3, C_{ZFE} and $C_{ZFE,w}$ are total and aqueous internal concentrations in the ZFE, respectively. Substituting equation 7 into equation 8 gives the predicted bioconcentration factor (eq. 8), which can be simplified because $C_w = C_{ZFE,w}$.

$$\begin{aligned} BCF_{MBM} &= \frac{C_{ZFE}}{C_w} \\ &= \frac{C_{ZFE,w}{*}\left(Vf_{lip}{*}D_{lipw}(pH\ 7.4)+Vf_{protein}{*}D_{SPw}(pH\ 7.4)+Vf_w\right)}{C_w} \quad (8) \\ &= Vf_{lip}{*}D_{lipw}(pH\ 7.4)+Vf_{protein}{*}D_{SPw}(pH\ 7.4)+Vf_w \end{aligned}$$

Further model inputs were physicochemical properties of the chemicals and the body mass parameter of the embryo (Vf_{lip} = 0.0146 kg_{lip}/L_{embryo}, $Vf_{protein}$ = 0.0823 kg_{lip}/L_{embryo}, V_w = 0.9031 kg_{lip}/L_{embryo}).[6] The pK_a was used to calculate the speciation of ionizable chemicals to correct the K_{lipw} for the speciation at pH 7.4 and to derive the D_{lipw}. The D_{SPw} was the measured or

predicted value for the neutral species and not corrected for speciation. These data were previously partially measured and partially collated from literature by Huchthausen et al. (2024)[7] (Excel file, sheet S1).

3. Internal membrane concentrations

The total internal lethal concentration causing 50 % of mortality (ILC_{50}) is calculated by multiplication of both experimental or predicted BCF and external LC_{50} of the exposed ZFEs (LC_{50}) (eq. 9).

$$ILC_{50}\left[\frac{\text{mmol}}{L_{\text{embryo}}}\right] = \text{BCF}\left[\frac{L_{\text{medium}}}{L_{\text{embryo}}}\right] * LC_{50}\left[\frac{\text{mmol}}{L_{\text{medium}}}\right] \quad (9)$$

Based on ILC_{50} , logD_{lipw} (pH 7.4) and logD_{SPw} (pH 7.4) of the chemical and measured body mass parameter of the ZFE[6], the mass fraction of the chemical in the cellular membrane ($f_{\text{lip,internal}}$) can be estimated (eq. 10):

$$f_{\text{lip,internal}} = \frac{1}{1+\frac{1}{D_{\text{lipw}}(\text{pH }7.4)}\frac{Vf_{w}}{Vf_{\text{lip}}}+\frac{D_{\text{SPw}}(\text{pH }7.4)}{D_{\text{lipw}}(\text{pH }7.4)}\frac{Vf_{MP}}{Vf_{\text{lip}}}} \quad (10)$$

Based on $f_{\text{lip,internal}}$, the volume fraction of the lipid phase in the ZFE (Vf_{lipw}) and the ILC_{50}, the internal membrane concentration causing 50 % mortality ($ILC_{50,\text{membrane}}$) can be calculated (eq. 11). Chemicals showing an $ILC_{50,\text{ membrane}}$ around a critical membrane concentration of 226±178 mmol/kg_{lipid} ($ILC_{50,\text{baseline toxicity}}$)[8] are considered as baseline toxicants, while chemicals showing a > factor of 10 lower $ILC_{50,\text{ membrane}}$ are proposed to exhibit a specific MOA.

$$ILC_{50,\text{membrane}}\left[\frac{\text{mmol}}{kg_{\text{lipid}}}\right] = \frac{f_{\text{lip,internal}} * ILC_{50}\left[\frac{\text{mmol}}{L_{\text{embryo}}}\right]}{Vf_{\text{flip}}} \quad (11)$$

4. Toxic ratio analysis

The intrinsic toxicity of a chemical can be described by the toxic ratio ($TR_{external}$) calculated by division of the predicted LC_{50} for baseline toxicity (eq.12) and the experimental aqueous LC_{50} (eq. 13). A TR value higher than 10 indicates a specific or reactive mode of action of a chemical.[9,10]

$$LC_{50,\text{baseline toxicity}} = 10^{(-0.99 * \log D_{lipw} - 2.22)} \quad (12)$$

$$TR_{external} = \frac{LC_{50,\text{ baseline toxicity}}}{LC_{50,\text{ experimental}}} \quad (13)$$

For internal measure of the intrinsic potency of a chemical, $TR_{internal}$ is determined as a ratio of the $ILC_{50,membrane}$ and the $ILC_{50,\text{baseline toxicity}}$ of 226 ± 178 mmol/kg_{lipid} (eq.14).

$$TR_{internal} = \frac{226\ \text{mmol/kg}_{lipid}}{ILC_{50,\text{ experimental}}} \quad (14)$$

5. Quality assurance and quality control

5.1 Stability assessment

In total, 8 of 63 test substances showed recoveries of <75%, 7 of which were less than 50% after 96 hours of incubation (**Figure S1 A**). However, when these chemicals were incubated under the same conditions in HPLC glass vials, recoveries increased significantly. Thus, the reason for unstable aqueous concentration of these 7 compounds was most likely adsorption to the plastic plate. Therefore, for these chemicals with <50% recovery in plastic, exposure experiments were conducted using crystallization dishes (7.5 cm diameter), with a proportion of one embryo/0.4mL.

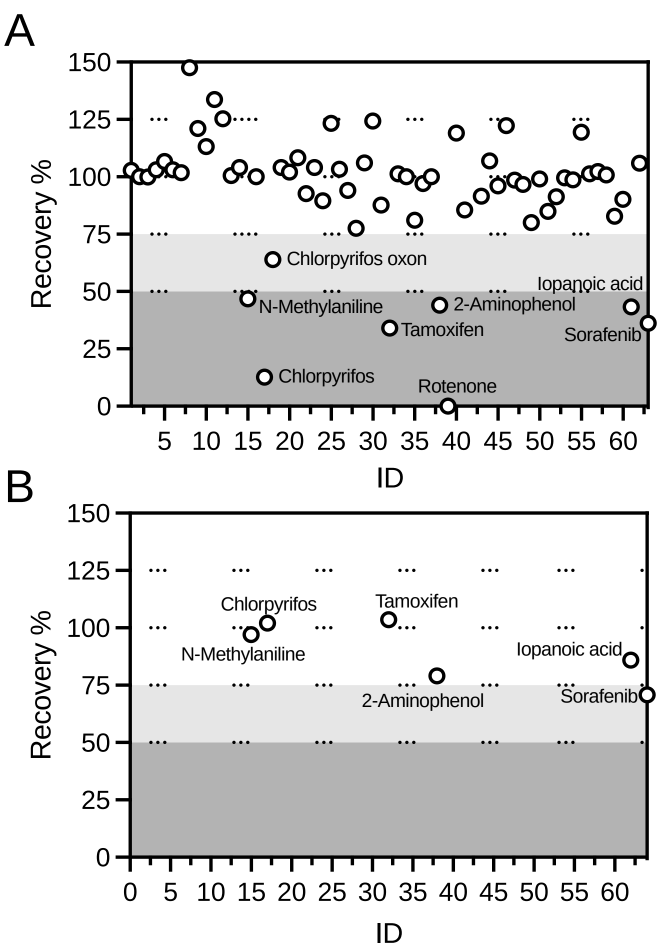

Figure S1: Assessment of recoveries of test compounds in and **A** 96 well plates (plastic) and **B** HPLC glass vials. 63 chemicals were tested at 1 mg/L nominal concentration and incubated at 28 ± 1 °C for 96 h.

5.2 Contribution of adsorption to the ZFE to the detected extract concentration

To investigate how much chemical remains on the surface of ZEF after washing with ISO water, a short-term-exposure (less than 30 s) of ZFE at 96 hpf with 1 mg/L mixture of 63 study chemicals was conducted according to Brox et al..[11] It was supposed that such a short exposure time would not be sufficient for a significant uptake of the test chemicals into the embryonic body. Samples were processed according to section 2.5 and stored at -20 °C until chemical analysis.

No analyte showed a critical sorption tendency to the ZFE affecting the extract concentration. Only for the very hydrophobic chemicals as Chlorpyriphos, Verapamil, Picoxystrobin, Rotenone, and Tamoxifen, concentrations up to 1.5 ng/organism were detected in ZFE extracts after short-term exposure (**Figure S2 B**). However, relative to the detected concentration after 96 hours of exposure the contribution of sorption to the total extract concentration is almost negligible (**Figure S2 D**).

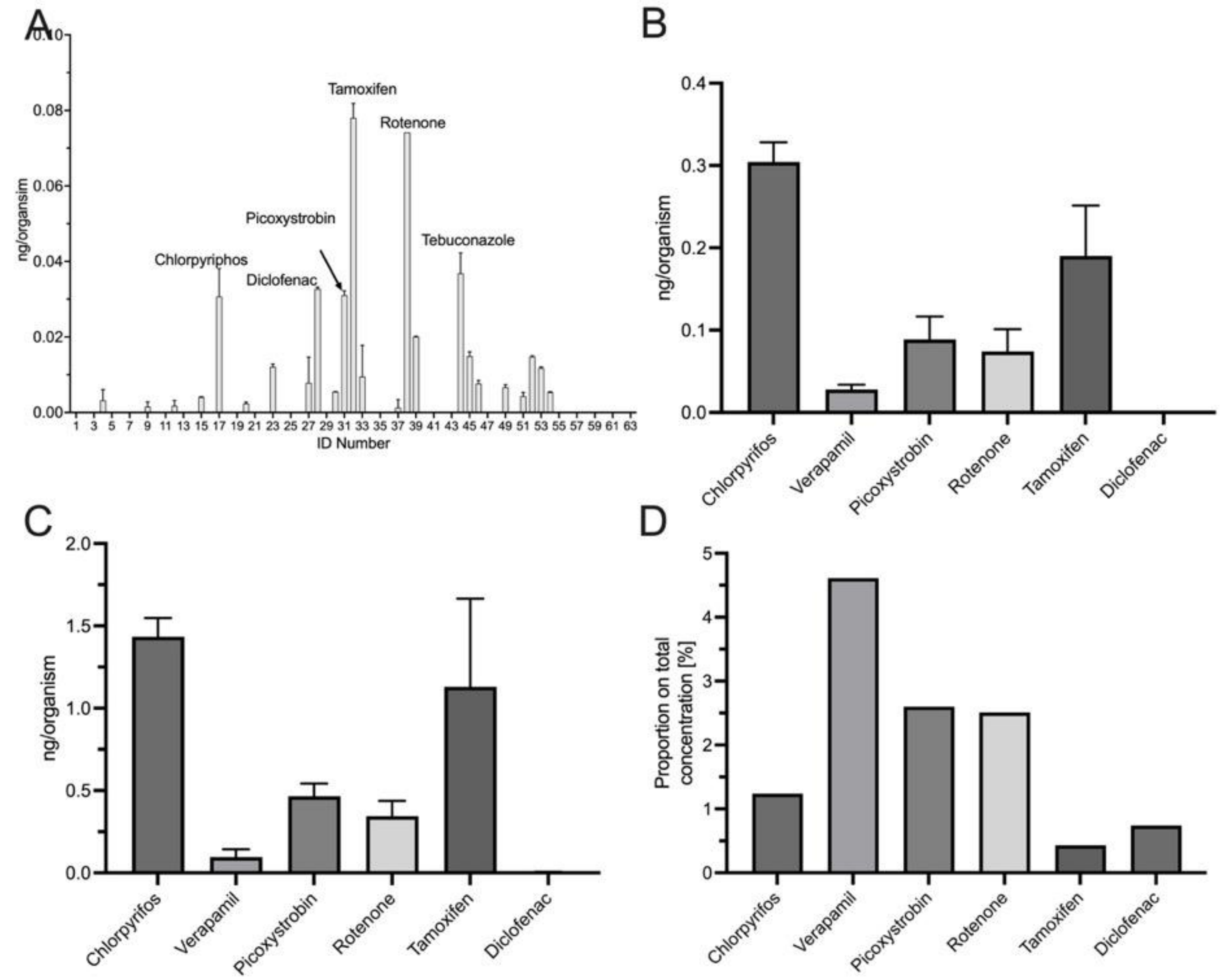

Figure S2: Influence of adsorption to the ZFE on the detected internal concentration. Remaining concentrations of **A** all study chemicals (n = 2) and **B** only hydrophobic chemicals (n = 2) after a short term exposure to a 0.1 mg/L solution. **C** Remaining concentration of hydrophobic study compounds after short-term exposure to a solution of 1 mg/L (n = 3). **D** Contribution of adsorption of test compounds to internal concentration analysis. To quantify the contribution of adsorption a short-term exposure of 30 s (external concentration 1 mg/L) and washing with ISO water was conducted with 96 hpf embroys and compared to concentration found after 96 h of exposure.

6. Instrumental parameters

6.1 HPLC-MS/MS analysis

Quantification of the study chemicals was performed using an Agilent 1290 HPLC system (Agilent) equipped with an Atlantis T3 C_{18}-phase column (2.1 mm x 50 mm, 3 µm; Waters) with an Atlantis T3 Security Guard column (2.1 x 10 mm, Waters) connected to a Qtrap 5500 (Sciex) triple quadrupole MS by multiple reaction monitoring (MRM). MRM parameters are provided in **Table S5**. Processed samples were diluted to the calibration range of the analytes. The final samples contained a water-MeOH ratio of 1:1 (v/v) for chemical analysis. For the determination of calibration curves, a 1/x weighted regression was used.

6.1.1 Mass spectrometric parameters

For targeted analysis, scheduled MRM mode was used. Molecular ions were scanned in 600 cycles with a cycling time of 1.5 seconds. The MRM detection window was set to 90 sec and the target scan time to 0.5 sec. MRM detection window was 20 sec. For scheduled MRM, the minimum dwell was 3 ms and maximum dwell was 250 ms.

Analytes were detected in both positive and negative ionization modes. The source parameters were: : gas 1 at 50 psi, gas 2 at 50 psi and curtain gas at 40 psi. The temperature of the ion source was set to 550 °C and the ionization voltage was 5500 V. The entrance potential was 10 V for positive mode and -10 V for negative mode. Compound-specific MRM parameters are provided in the following table. Single reaction monitoring was performed for valproic acid.

Table S5: Compound-specific parameter for MRM transitions of study compounds including retention times.

**Analyte**	**ESI mode**	**Precursor m/z**	**Product ion m/z**	**RT [min]**	**DP [V]**	**EP [V]**	**CE [V]**	**CXP [V]**
Caffeine	positive	194.9	137.9	2.3	80	10	27	8
		194.9	110.0	2.3	80	10	31	18
Chlorpyrifos	positive	349.9	96.7	4.05	40	10	47	6
		349.9	197.9	4.05	40	10	29	10
N-methylaniline	positive	108.0	93.1	0.75	91	10	15	8
		108.0	66.1	0.75	91	10	35	8
Methylacrylamide	positive	86.0	58.1	0.97	56	10	17	8
		86.0	41.1	0.97	56	10	23	6
N,N-diethylacrylamide	positive	128.0	74.1	2.61	61	10	19	8
		128.0	55.0	2.61	61	10	23	8
N,N-methylenediacrylamide	positive	155.0	72.2	1.29	36	10	13	6
		155.0	81.0	1.29	36	10	39	10
N-butoxymethyl-acrylamide	positive	158.03	55.0	2.97	56	10	23	8
		158.0	84.0	2.97	56	10	9	10
Verapamil	positive	454.8	165.1	3.14	201	10	37	10
		454.8	150.0	3.14	201	10	59	10
Ethylenethiourea	positive	102.9	44.0	0.56	61	10	23	6
		102.9	60.0	0.56	61	10	41	8
Atorvastin	positive	559.1	440.3	3.7	111	10	31	12
		559.1	250.2	3.7	111	10	61	20
Chlorpyriphos oxon	positive	333.9	277.9	3.84	81	10	25	24
		333.9	198.0	3.84	81	10	43	16
Picoxystrobin	positive	368.1	145.2	3.8	56	10	31	12
		368.1	205.1	3.8	56	10	13	16
Niflumic acid	positive	283.0	265.1	3.87	66	10	31	20

		283.0	245.0	3.87	66	10	45	20
Dexamethasone	positive	393.1	91.1	3.59	86	10	89	12
		393.1	147.2	3.59	86	10	39	12
Diphenylamine	positive	170.1	93.1	3.79	91	10	35	10
		170.1	92.0	3.79	91	10	27	8
1,2-dimethylimidazole	positive	97.0	56.0	0.37	51	10	23	8
		97.0	61.1	0.37	51	10	25	8
1-methylimidazole	positive	82.9	69.0	0.37	101	10	9	6
		82.9	42.0	0.37	101	10	25	6
2-ethyl-4-methyl-imidazole	positive	111.0	96.0	0.81	71	10	25	8
		111.0	56.2	0.81	71	10	25	6
2-methylimidazole	positive	82.9	68.9	0.5	126	10	11	8
		82.9	56.0	0.5	126	10	27	8
4-methylimidazole	positive	82.9	68.9	0.5	101	10	9	6
		82.9	56.2	0.5	101	10	25	4
		82.9	42.1	0.4	101	10	33	6
Imidazole		68.9	68.9	0.43	100	10	5	6
Genistein	positive	271.0	153.0	3.5	71	10	39	12
		271.0	91.1	3.5	71	10	51	12
Rotenone	positive	395.0	213.1	3.84	91	10	33	16
		395.0	192.1	3.84	91	10	33	16
o-Aminophenol	positive	110.0	65.0	0.48	101	10	29	8
		110.0	92.1	0.48	101	10	21	8
Cyproconazole	positive	292.0	70.2	3.73	96	10	25	6
		292.0	125.0	3.73	96	10	43	12
Imazalil	positive	297.0	159.0	3.15	101	10	33	14
		297.0	201.0	3.15	101	10	25	18
NMPMA	positive	158.0	55.0	2.96	56	10	25	8
		158.0	84.0	2.96	56	10	11	8
Triadimenol	positive	296.0	70.1	3.73	106	10	27	10
		296.0	98.9	3.73	106	10	21	48
Acetaminophen	positive	152.0	110.1	1.53	91	10	23	10
		152.0	65.1	1.53	91	10	39	8
DMF	positive	74.0	46.1	0.71	46	10	17	8
		74.0	74.0	0.71	46	10	5	6
NBHENPD	positive	242.1	146.1	1.32	26	10	29	10
		242.1	166.1	1.32	26	10	31	14
Atrazine	positive	216.1	174.1	3.55	126	10	25	14
		216.1	104.0	3.55	126	10	39	14
Tamoxifen	positive	372.1	72.0	3.53	171	10	29	8
		372.1	172.0	3.53	171	10	61	18
2-ethylimidazole	positive	97.0	82.0	0.41	101	10	29	10
		97.0	69.0	0.41	101	10	23	8

Azoxystrobin	positive	404.0	372.1	3.64	61	10	21	32
		404.0	344.1	3.64	61	10	33	30
Colchicine	positive	400.1	358.1	3.27	91	10	31	10
		400.1	382.1	3.27	91	10	29	30
Propylthiouracil	positive	171.00	154.0	2.25	61	10	23	14
		171.00	112.1	2.25	61	10	27	12
Tebuconazole	positive	308.05	125.0	3.82	96	10	55	12
		308.05	89.0	3.82	96	10	91	10
1-Vinylimidazole	positive	95.0	69.0	0.38	86	10	29	8
		95.0	42.0	0.38	86	10	37	6
Lidocaine	positive	235.1	86.0	1.92	96	10	23	14
Carbamazepine	positive	237.0	194.1	3.44	106	10	25	6
		237.0	193.1	3.44	106	10	47	8
Carbendazim	positive	192.0	160.0	1.77	76	10	25	14
		192.0	132.0	1.77	76	10	41	12
Methotrexate	positive	455.2	308.2	1.99	116	10	27	26
		455.2	175.1	1.99	116	10	51	16
Cylcophosphamide	positive	261.1	183.0	3.14	61	10	15	16
		261.1	140.1	3.14	61	10	29	12
Citalopram	positive	325.2	109.1	2.97	51	10	35	14
		325.2	262.1	2.97	51	10	27	22
Mebendazole	positive	296.1	264.0	3.44	161	10	31	16
		296.1	105.0	3.44	161	10	43	12
Chlorpromazine	positive	319.1	241.1	3.33	71	10	11	16
		319.1	86.1	3.33	71	10	29	12
Haloperidol	positive	376.1	165.2	3.1	96	10	33	14
		376.1	358.1	3.1	96	10	27	26
Imidalcloprid	positive	256.2	209.0	2.49	76	10	21	16
		256.2	175.1	2.49	76	10	27	10
Thiamethoxam	positive	291.9	211.1	2.11	126	10	17	18
		291.9	181.1	2.11	126	10	31	14
Metyrapone	positive	227.1	106.1	2.10	121	10	41	18
		227.1	121.2	2.10	121	10	29	18
Iopanoic acid	positive	571.7	445.0	3.93	96	10	19	16
		571.7	318.0	3.93	96	10	33	26
Sorafenib	positive	464.9	252.1	3.85	91	10	47	30
		464.9	387.1	3.85	91	10	15	40
Diclofenac	negative	295.9	252.0	3.72	-40	-10	-16	-11
		295.9	250.0	3.72	-40	-10	-16	-13
Clofibirc acid	negative	212.9	126.9	3.41	-65	-10	-20	-15
		212.9	85.0	3.41	-65	-10	-14	-11
Phenytoin	negative	251.0	101.8	3.37	-15	-10	-28	-7
		251.0	207.9	3.37	-15	-10	-24	-9

Fipronil	negative	435.0	330.0	3.79	-80	-10	-23	-10
		435.0	250.0	3.79	-80	-10	-40	-8
Valproic acid*	negative	143.0	143.0	4.69	-55	-10	-10	-9
Triatricol	negative	576.6	126.9	3.76	-150	-10	-26	-19
		576.6	448.9	3.76	-150	-10	-12	-9
Naproxen	negative	229.0	184.8	3.65	-50	-10	-8	-33
		229.0	169.5	3.65	-50	-10	-26	-55

*Different mobile phase was used

6.1.2 High-performance liquid chromatography

Determination of internal concentrations was performed on a 1290 Infinity HPLC system (Agilent Technologies, Böblingen, Germany) coupled to a Qtrap 5500 triple-quadrupole mass spectrometer (AB Sciex, Darmstadt, Germany) equipped with a TurboIonSpray interface. Measurements were controlled by Analyst (version 1.5.2, AB Sciex). The LC system was equipped with a degasser, a binary pump with high-pressure gradient mixer, a thermostatic autosampler (temperature set to 10 °C) and a column oven (set to 35 °C). The injection volume was 10 µL. The chromatographic separation was conducted on an Atlantis T3 C_{18}-phase column (2.1 mm x 50 mm, 3 µm; Waters, Eschborn, Germany) with an Atlantis T3 Security Guard column (2.1 x 10 mm, Waters, Eschborn, Germany). The eluents were MilliQ water with 0.1% formic acid (eluent A) and methanol with 0.1% formic acid (eluent B). Elution of analytes was performed with 0.5 mL/min flow rate using the following gradient program: 0.0 min, 10 % eluent B; 2 min, 50 % eluent B; 3 min, 100 % eluent B; 6 min, 100 % eluent B; 6.1 min, 10 % eluent B; 15 min, 10 % eluent B. For valproic acid, a different mobile phase was used, but the same separation method as follows: MilliQ water (eluent A), MeOH with 5 mM ammonium formate (eluent B).

6.1.3 Limits of detection, limits of quantification and linear dynamic range

A standard mix with a concentration of 10 µg/mL in MeOH was used to determine the limit of detection (LOD) and limit of quantification (LOQ). Depending on the linear range of each chemical, calibration solutions ranging from 0.05 to 250 ng/mL were prepared in ZFE matrix (8 ZFE per concentration) from this standard and measured three times via HPLC-MS/MS. Each calibration standard had a water/MeOH ratio of 1:1 (v/v). The LOD and LOQ were then calculated from the peak areas obtained using equations (15) and (16).

$$LOD = \frac{s_y}{b} * t * \sqrt{\frac{1}{n} + \frac{1}{m} + \frac{\bar{x}^2}{\sum_{i=1}^{n}(x_i - \bar{x})^2}} \quad (15)$$

$$LOQ = k * \frac{s_y}{b} * t * \sqrt{\frac{1}{n} + \frac{1}{m} + \frac{(k * x_N - \bar{x})^2}{\sum_{i=1}^{n}(x_i - \bar{x})^2}} \quad (16)$$

Legend:

s_y = standard deviation

t = value of t-distribution

b = slope of calibration curve

n = number of calibration values

m = number of replicates

x_i= measured value

$\bar{x}$ = average

The obtained LOD, LOQ and linear dynamic range (LDR) data are summarized in the following table.

Table S6: LOD, LOQ and LDR data for each study chemicals.

**Chemical**	**LDR [ng/mL]**	**LOD [ng/mL]**	**LOQ [ng/mL]**	R^2
Ethoprofos	2 - 250	1.49	7.22	0.9993
Pirinixic acid	2 - 250	1.50	7.50	0.9996
Methylenebisacrylamide	1 - 20	0.80	3.70	0.9955
N-butoxy-methyl-acrylamide	0.5 - 35	0.45	2.10	0.9975
NMPMA	0.5 - 35	0.25	1.20	0.9935
Diethylacrylamide	1.0 - 35	0.68	3.16	0.9979
Methacrylamide	1.0 - 35	0.26	1.12	0.9939
imidazole	1.5 - 50	1.30	5.80	0.9899
1-methylimidazole	0.5 - 50	0.25	1.20	0.9983
4-methylimidazole	0.25 - 50	0.18	0.80	0.9953
1,2-dimethylimidazole	0.5 - 35	0.46	2.31	0.9945
2-ethyl-4-methylimidazole	2.5 - 50	2.00	9.20	0.9918
Caffeine	0.5 - 15	0.36	1.74	0.9978
N-methylanilline	0.25 - 25	0.10	1.00	0.9951
Triadimenol	2.5 - 35	0.97	4.43	0.9837
Chlorpyrifos	2.5 - 25.0	1.50	6.70	0.9978
Chlorpyrifos oxon	1.0 - 25	0.60	2.80	0.9872
Genistein	1.0 - 250	0.56	2.60	0.9974
Niflumic acid	0.5 - 25	0.45	2.10	0.9946
Dexamethasone	1.0 - 250	0.77	3.57	0.9947

Ethylenethiourea	0.5 - 20	0.48	2.20	0.9945
Diphenylamine	1.0 - 35	0.65	3.71	0.9862
Atrovastatin	1.0 - 100	0.56	2.80	0.9987
Valproic acid	5 - 250	3.17	15.31	0.9996
Verapamil	2.5 - 35	1.10	5.00	0.9948
Diclofenac	1.0 - 35	0.76	3.47	0.9942
Clofibric acid	0.5 - 35	0.42	1.99	0.9936
Carbendazim	0.5 - 35	0.48	2.27	0.9977
Picoxystobin	0.5 - 100	0.37	1.70	0.9984
Tamoxifen	2.5 - 25	1.30	5.90	0.9889
Imazalil	0.5 - 35	0.50	1.20	0.9852
DMF	1.5 - 15	1.50	6.70	0.9969
NBHENPD	0.5 - 35	0.30	1.60	0.9965
Cyproconazole	1.0 - 35	0.62	2.85	0.9907
o-Aminophenol	0.5 - 35	0.40	1.80	0.9952
Rotenone	0.5 - 35	0.50	2.10	0.9959
Atrazine	0.5 - 50	0.36	1.70	0.9923
Acetaminophen	0.25 - 50	0.20	0.95	0.9914
2-ethylimidazole	0.25 - 25	0.20	0.90	0.9956
1-vinylimidazole	0.5 - 70	0.22	1.05	0.9984
Phenytoin	2.5 - 35	1.88	8.63	0.9917
Tebuconazole	2.5 - 35	1.53	6.77	0.9930
2-methylimidazole	1.0 - 60	0.60	2.90	0.9952
Haloperidol	0.5 - 35	0.24	1.12	0.9963
Chlorpromazine	1 - 35	0.76	3.78	0.9806
Carbamazepine	0.5 - 25	0.30	1.41	0.9978
Imidalcloprid	1 - 35	0.53	2.49	0.9981
Fipronil	1 - 50	0.79	3.32	0.9949
Thiamethoxame	0.5 - 25	0.32	1.52	0.9929
Azoxystrobin	0.5 - 35	0.50	2.50	0.9967
Mebendazole	0.5 - 35	0.29	1.37	0.9960
Citalopram	0.5 - 35	0.38	1.78	0.9941
Lidocaine	0.5 - 50	0.36	1.70	0.9964
Cyclophosphamide	0.5 - 50	0.34	1.59	0.9965
Propylthiouracil	0.5 - 30	0.36	1.70	0.9972
Colchicine	1.0 - 35	0.84	1.67	0.9969
Methotrexate	1.0 - 35	0.95	4.29	0.9958
Metapyrone	1.0 – 100	0.50	2.34	0.9981
Iopanoic acid	0.5 – 100	0.33	0.67	0.9993
Naproxen	10 - 100	4.64	9.28	0.9975
Sorafenib	0.5 – 100	0.50	1.0	0.9983

6.1.4 Matrix effects and recovery

a) Sample preparation for evaluation of matrix effects

To assess matrix effects, three replicates consisting of 8 ZFE for three standard solutions in concentrations of 5 ng/mL, 10 ng/mL and 25 ng/mL at 96 hpf were used. For this purpose, 8 ZFEs per replicate were collected in a FastPrep tube and extracted according to the section 2.5 in the manuscript. Briefly, 150 µL of the supernatant was used and diluted with 150 µL of an aqueous standard mixture containing all 63 analytes. After matrix extraction, samples were stored at -20°C until LC-MS/MS analysis. Responses of matrix samples were compared to standards in pure solvent with same concentrations.

b) Sample preparation for determination of method recovery

The same approach as for the evaluation of matrix effects was used for ZFEs at 96 hpf. The difference was that MeOH standard mixtures were used for extraction, which was subsequently diluted with the same amount of water.

c) Results – Matrix effects and recovery

The mean peak area for all analytes in all samples relative to the peak area in pure solvent was 95 %. Significantly higher matrix effects were observed for very hydrophilic substances such as 1-methylimidazole and methylene-bis-acrylamide with matrix recoveries of 25 and 155 %, respectively. However, beside these extreme examples, most matrix recoveries ranged between 75 and 100 %, indicating that for most of the detected concentrations were not substantially affected by matrix effects. Significant differences in matrix effects between developmental stages of the ZFE is not expected as demonstrated by Brox et al.[11] and Grasse et al..[1] Method recoveries ranged from 23 to 139 %, similar to the matrix effect data. Recoveries for most substances were between 75 and 100 %.

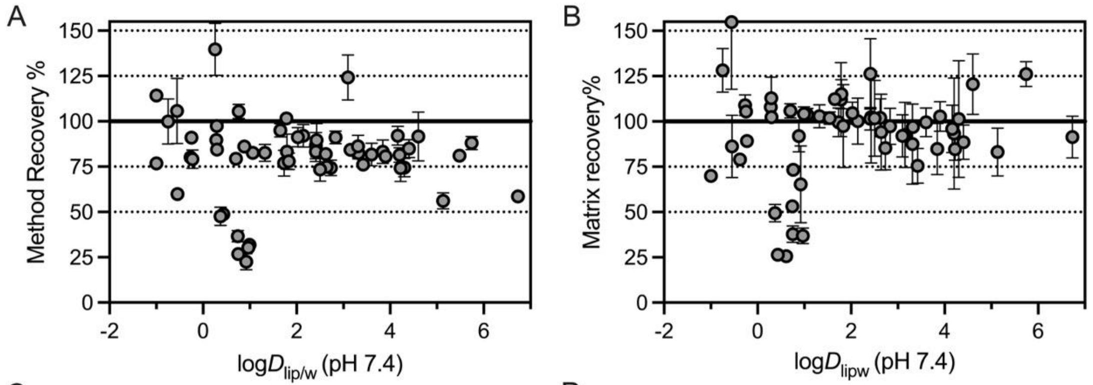

C

Chemical	logD_{lipw}	Method recovery %
2-methylimidazole	0.92	23
4-methylimidazole	0.75	27
1-Vinylimidazole	0.97	30
1,2-dimethylimidazole	0.99	32
o-Aminophenol	0.74	37
2-ethylimidazole	0.37	48
2-methylimidazole	0.43	49

D

Chemical	logD_{lipw}	Matrix recovery %
1-methylimidazole	0.61	26
4-methylimidazole	0.43	26
1-vinylimidazole	0.97	37
imidazole	0.75	38
2-ethylimidazole	0.37	49
o-aminophenol	0.74	53

Figure S3: A Method recovery (n = 3; 25 ng/mL standard mix) and **B** matrix effects (n = 3, 20 ng/mL standard mix) of 63 test compounds in extracts of eight ZFEs. Chemicals showing <50 % recovery **C** and strong (<50% matrix recovery) matrix effects **D**.

6.2 UPLC-QTOF-MS

6.2.1 Detection of transformation products (TPs) using UPLC-QTOF-MS

For the identification of TPs, ZFE extracts were analyzed by ultra-performance liquid chromatography time-of-flight mass-spectrometry (UPLC-TOF-MS) using a AQUITY UPLC I-Class system (Waters) equipped with a HSS T3 column (100 x 2.1 mm, 1.7 µm) coupled to a XEVO G2S (Waters). The detailed instrumental conditions and the corresponding method can be found in the Table S8 . The ZFE extracts were injected without further dilution in water/MeOH (1:1, v/v).

Table S7: Instrumental parameters of UPLC-qTOF-MS method for transformation product analysis.

Parameter	Value
Flow rate	0.45 µL/min
Column temperature	45 °C
Capillary voltage	0.7 kV (positive mode)<br>- 1.3 kV (negative mode)
Source temperature	140 °C
Desolvation temperature	550 °C
Sampling cone voltage	20 V
Source offset	50 V
Cone gas	nitrogen
Collision gas	argon
Desolvation gas flow	950 L/h
Scan time	0.15 s
Collision energy	4 eV (molecular ions); 15 - 35 eV (fragments)
Mobile phase	A: MilliQ water + 0.1 % formic acid<br>B: methanol + 0.1 % formic acid
Solvent gradient	0 min 2% B, 12.25 min 99% B, 15.00 min 99% B; 15.10 min 2% B, 17.00 min 2% B.

6.2.2 Identification of TPs via suspect- and non-target-screening

TPs were identified using MarkerLynx (Waters, version 4.1). For suspect screening, Biotransformer 3.0 was used to predict potential metabolites of each study chemical. Predicted exact masses of potential TPs were searched in the m/z data set of exposed ZFEs and compared to the data of the control group. To detect a wide range of TPs, a non-target screening approach using MarkerLynx was used. More details on parameter settings can be found in Table S9. UPLC-HRMS data were evaluated in a retention time window of 1 to 10 min and a mass range of m/z 50 to 1200. The peak picking was conducted using MarkerLynx with a 0.1 min deviation in retention time and 0.01 Da deviation in the exact mass.

Chemical formulas were generated using a mass tolerance of 5 ppm and elemental composition of C (0-100), H (0-100), N (0-20), O (0-20), P (0-1), S (0-20), Cl (0-3,) Br (0-3), F (0-3) and Na (0-2). The results were exported to Excel and all further statistical analysis steps were performed there. A principal component analysis (PCA) using retention times, m/z data and the intensity of each peak was performed. For data visualization, GraphPad Prism was used. Newly appeared peaks in the exposed ZFE extracts compared to the unexposed ZFE samples and the solvent blanks were selected as candidate TPs. In addition, MS/MS fragment ions were considered for structure elucidation based on possible biotransformation that may happen to the parental compounds. If standards were available, MS/MS data and retention time of TPs in ZFE extracts were confirmed by comparing them with the reference standard.

Table S8: Parameter of MarkerLynx method used for detection of transformation products.

Property	Value
Function	1
Analysis type	Peak Detection
Initial retention time	1.0
Final retention time	10.0
Low mass	50.0
High mass	1200.0
XIC window (Da)	0.01
Use relative retention time	No
Apply smooting	No
Noise elimination level	0.0
Deisotope data	Yes
Replicate % Minimum	0.0

7. Correlations of internal concentrations with physicochemical properties

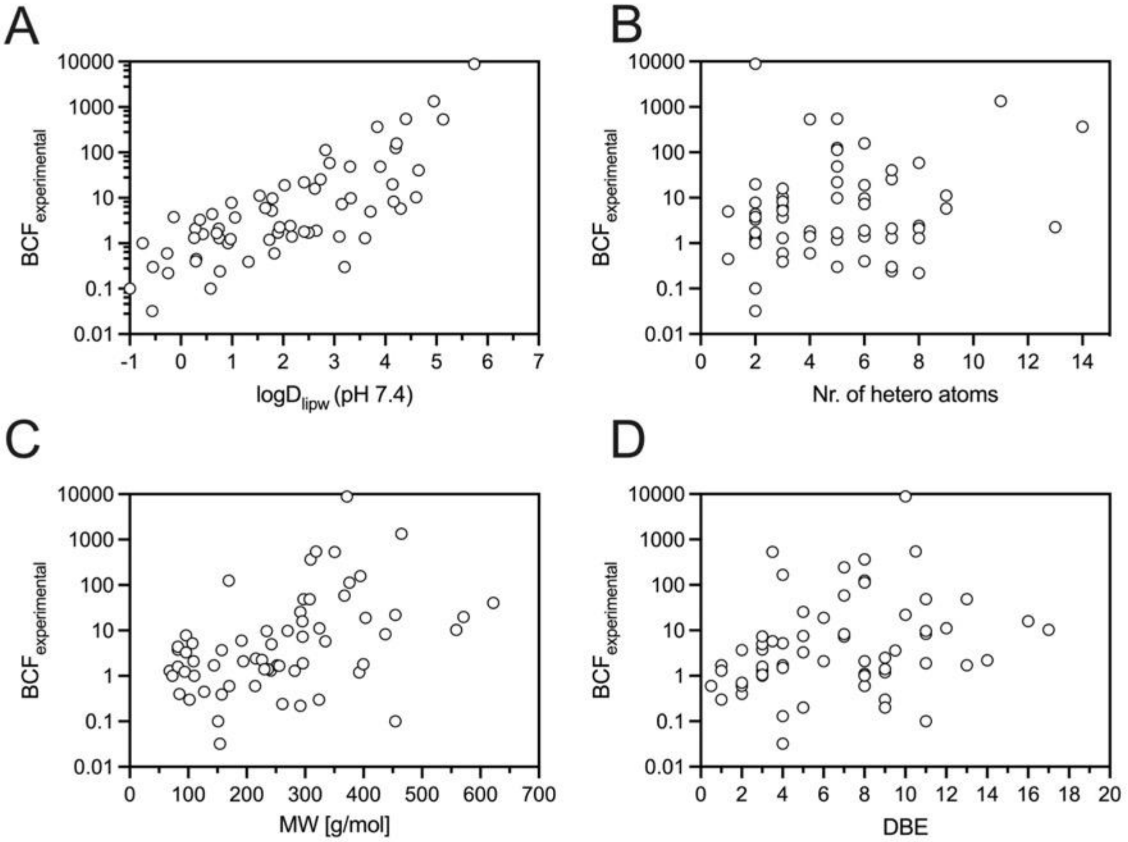

Figure S4. Dependency of observed bioconcentrations factors ($BCF_{experimental}$) to **A** hydrophobicity expressed as liposome-water distribution ratio (logD_{lipw}), **B** the number of heteroatoms of all 63 chemicals, **C** the molecular weight and **D** equivalents of double bonds (DBE) of all 63 study chemicals.

8. Non-target screening of transformation products

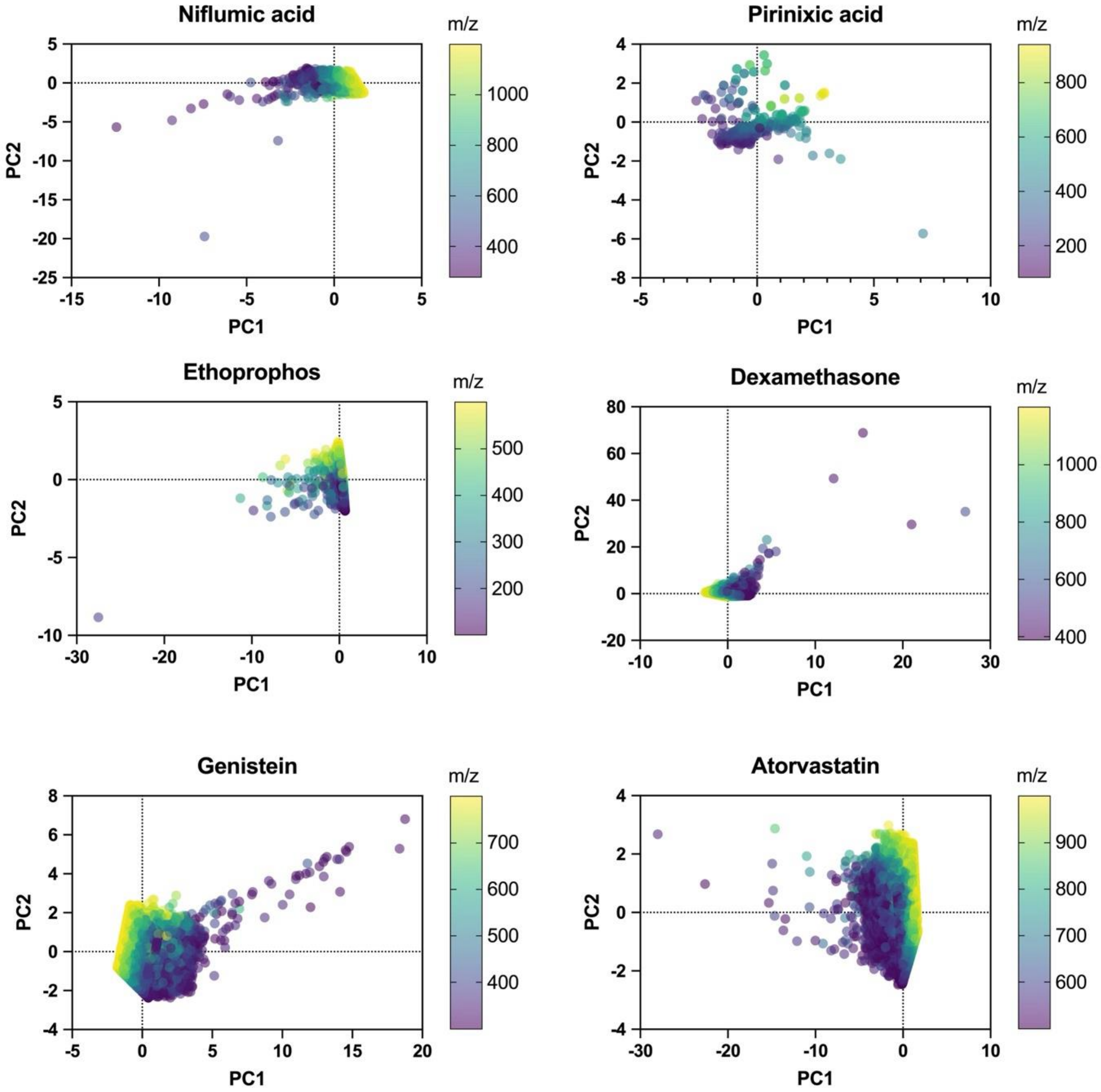

Figure S5: Feature analysis of analyzed extracts of ZFEs exposed to the respective chemical for 96 h and non-exposed ZFEs. Principle component analysis was performed using retention times, m/z data and the response of each signal in exposed and non-exposed ZFEs. Chemical analysis was conducted via UPLC-QTOF-MS. Mass spectrometric data were recorded in positive ionization and negative mode. Non-target screening (NTS) was performed for extracts of 8 ZFEs exposed to Niflumic acid, Pirinixic acid, Ethoprophos, Dexamethasone, Genistein and Atorvastatin. The exposure was started at 4 ± 1 hpf.

9. Specificity analysis based on toxic ratios and correlation between external and internal concentration of atorvastatin

Table S9: Study chemicals with known mode of action (MOA) with their predicted and experimental bioconcentration factors (BCF_{MBM} and $BCF_{Exp.}$), half-maximal lethal concentrations (LC_{50}), baseline toxicities ($LC_{50,baseline}$), critical membrane concentrations ($ILC_{50,membrane}$) and toxic ratios (TRs). Based on previous studies, these chemicals with known MOA in the ZFE were selected to verify the performance of the critical membrane concentration based MOA classification. TRs between 0.1 and 10 indicate baseline toxicity. TR > 10 indicate a specific or reactive mode of action and TR < 0.1 indicate experimental artefacts or strong internal degradation.

Chemical	Class	BCF_{MBM}	$BCF_{Exp.}$	LC_{50} [mM]	$LC_{50,\ baseline}$ [mM]	$ILC_{50,membrane}$ [mmol/kg_{lipid}]	TR_{extern}
Diphenylamine	Baseline toxicant[12]	291	125.1 ± 83.7	0.09	0.01	586	0.1
N-Methylaniline	Baseline toxicant[13]	2.1	5.2 ± 1.8	1.64	2.88	248	1.8
Tamoxifen	Baseline toxicant[14]	11361	8898 ± 950	0.01	3.45E-04	3009	0.05
Verapamil	Baseline toxicant[13]	7.6	22 ± 7	0.09	0.67	71	7.2
Chlorpyriphos oxon	Specific[15]	299	5.80 ± 4.09	6.25E-04	9.18E-03	0.24	14.7
caffeine	Specific[12]	1.0	2.1 ± 0.2	0.121	87.66	0.50	727
Carbendazim	Specific[16]	2.0	6.0 ± 0.4	2.50E-03	3.82	0.34	1527
Diclofenac	Specific[17]	31.8	1.9 ± 0.3	2.11E-03	0.39	0.06	186
Picoxystrobin	Specific[18]	29.2	58.6 ± 41.9	2.30E-04	0.22	0.38	949
Rotenone	Specific[12]	423.3	158.4 ± 62.1	1.09E-04	0.01	0.68	101
Acrylamides (4)	Reactive[19]	0.91 – 1.32	0.03 – 3.70	0.17 – 9.02	8.18 – 594	0.1 – 6.12	17 - 86

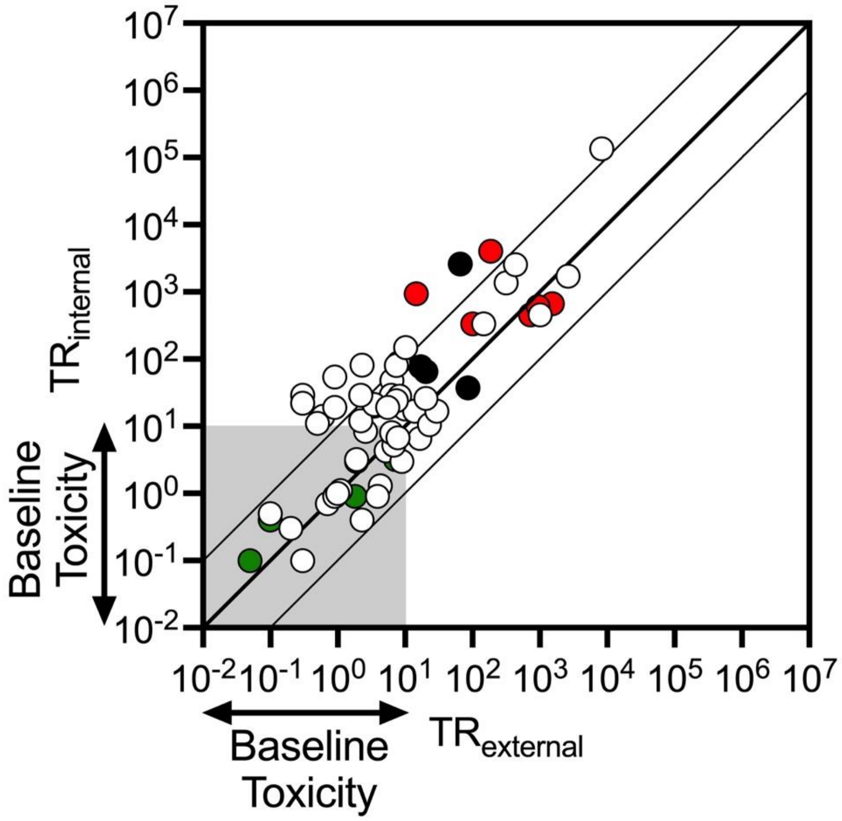

464
465 **Figure S6:** Comparison nominal $TR_{external}$ values from the FET using experimental aqueous LC_{50} data to $TR_{internal}$
466 based on experimental internal ILC_{50} data. Putative baseline and specific acting toxicants were used to check the
467 classification performance of both approaches.

468

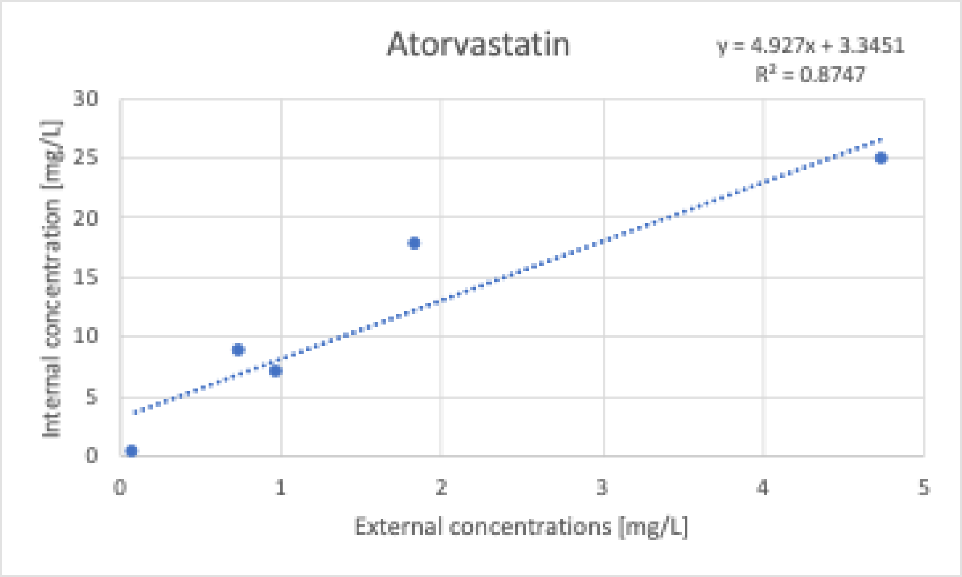

469
470 **Figure S7:** Correlation between external and internal concentrations of atorvastatin. Measured exposure
471 concentrations were 0.09; 0.99; 0.75; 1.85 and 4.75 mg/L. The exposure experiments were started at 4±1 hpf.

472

10. Extracted-ion-chromatograms, MS and MS/MS spectra of biotransformation products

Negative controls represent unexposed ZFEs which were extracted according to the extraction protocol used in the present study. “Extracts” is referred to extracted Matrix from ZFEs exposed to the respective chemical.

10.1 TPs of Pirinixic acid

Compound ID: PA_0

Compound name: Pirinixic acid (Parental Compound)

Chemical formula: $C_{14}H_{15}N_3O_2SCl$ (+) / $C_{14}H_{13}N_3O_2SCl$ (-)

m/z: 324.0580 (+) / 322.042 (-)

Retention time: 9.98 min

Confidence level: 3

Proposed chemical structure:

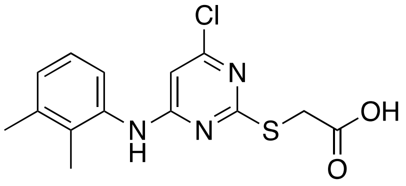

XIC:

Positive mode:

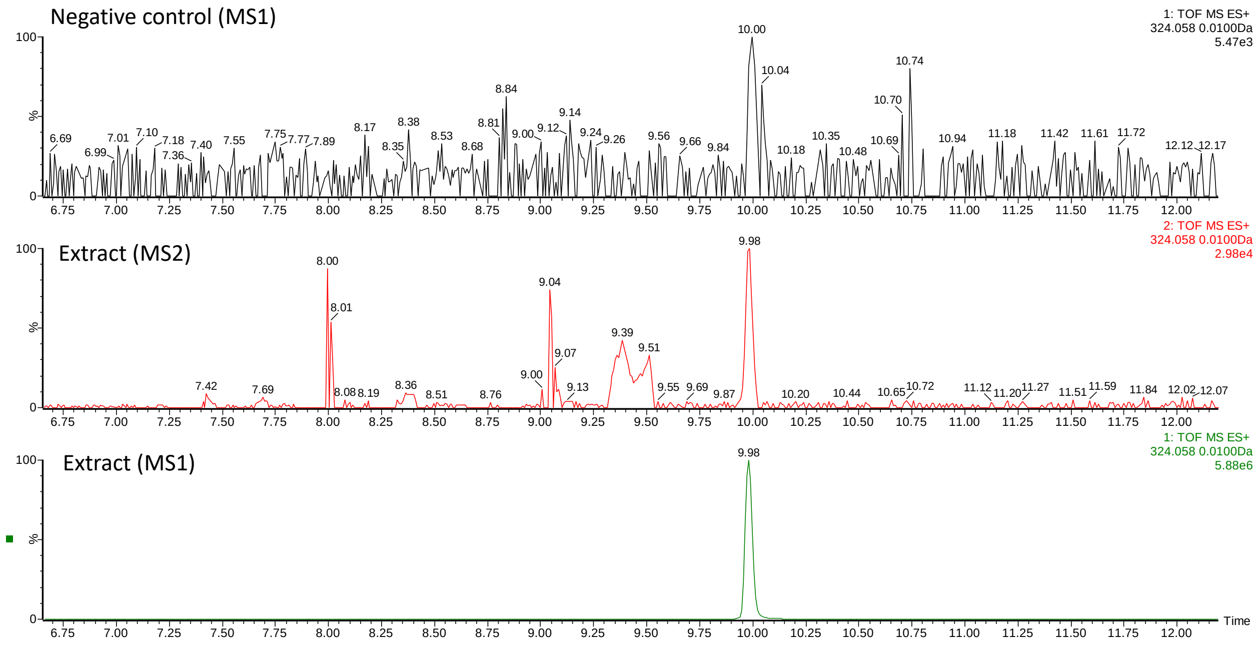

MS/MS spectrum:

Positive mode:

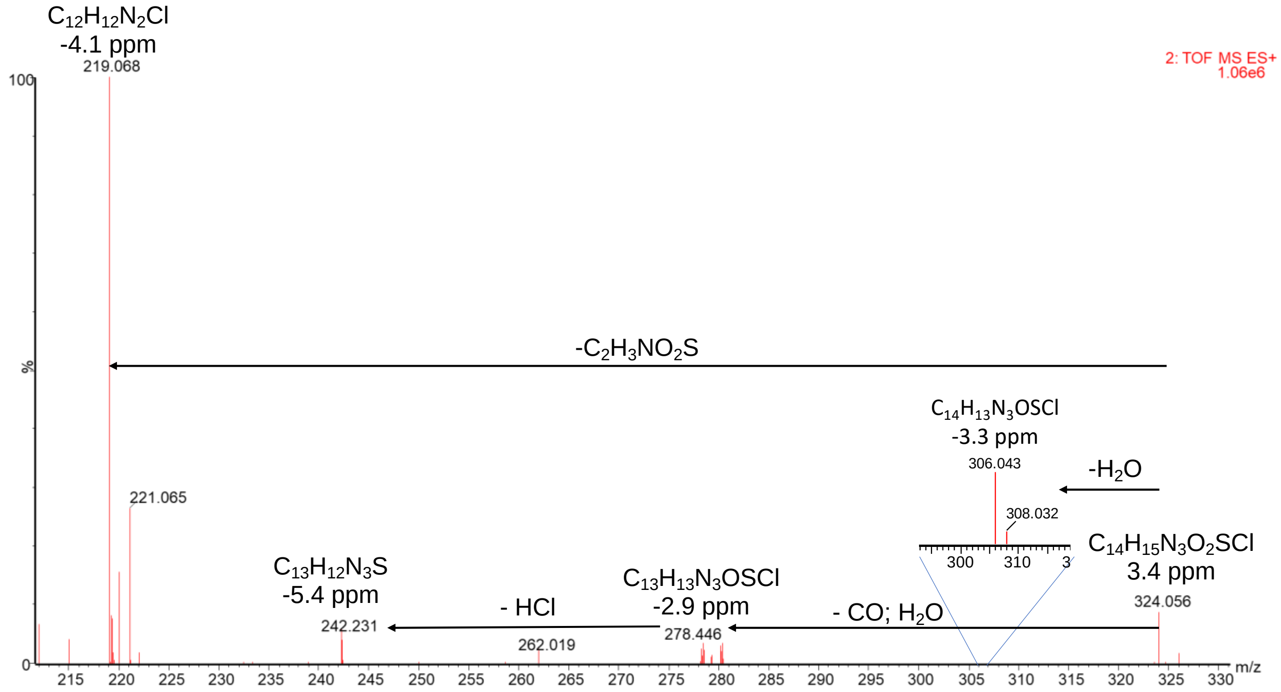

Reference spectrum: Mass Bank; MSBNK-EPA-ENTACT_AGILENT002046

Compound ID: PA_1

Compound name: PA+Glucuronide

Chemical formula: $C_{20}H_{23}N_3O_8SCl$ (+) / $C_{20}H_{21}N_3O_8SCl$ (-)

m/z: 500.0888 (+) / 498.0728 (-)

Retention time: 9.38+9.51 min (+) / 9.38+9.49 min (-)

Confidence level: 4

Proposed chemical structure:

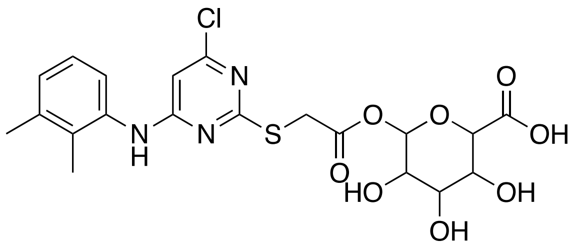

XIC:

Positive mode:

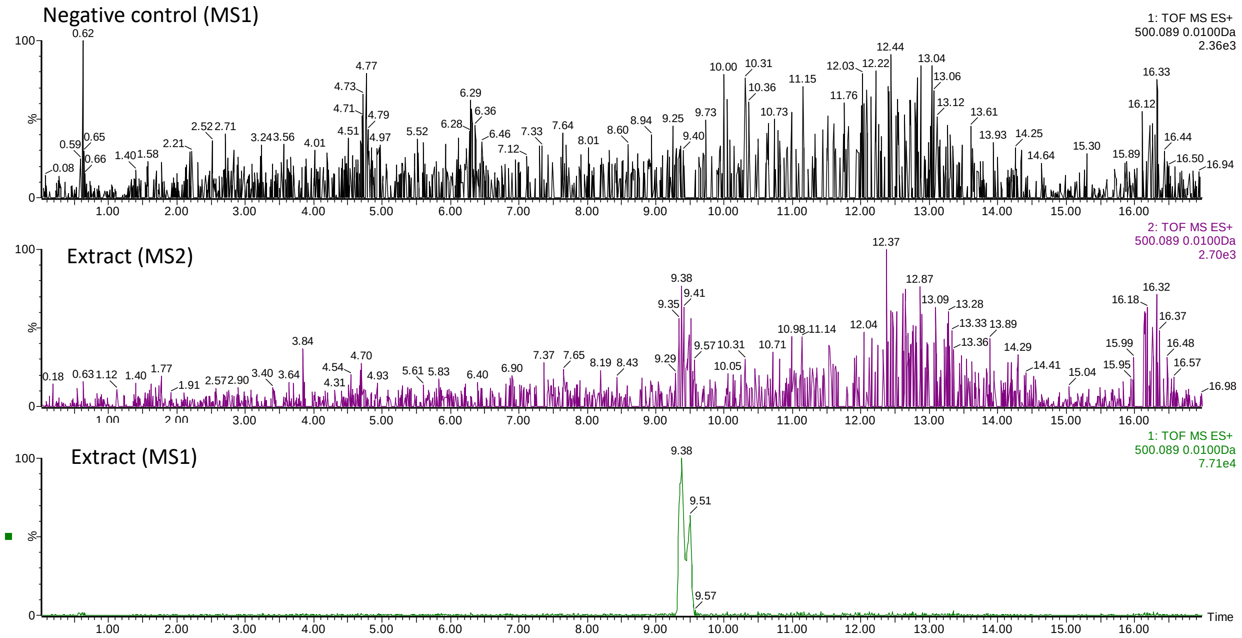

MS spectrum:

Positive mode

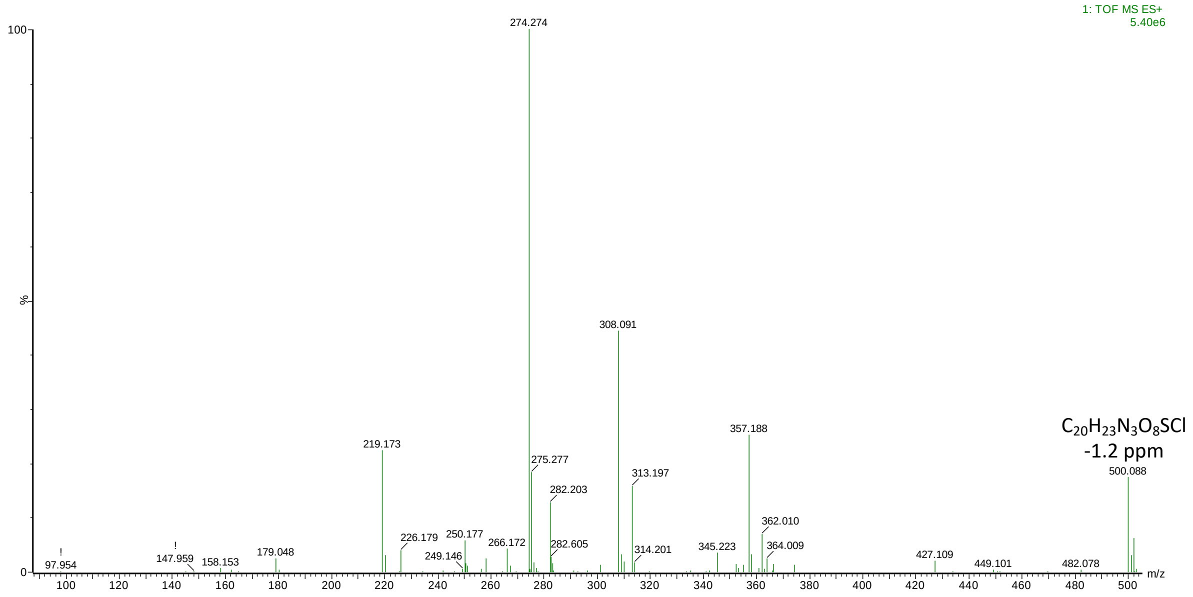

Compound ID: PA_2

Compound name: PA+O

Chemical formula: $C_{14}H_{14}N_3O_3SClNa$ (+) / $C_{14}H_{13}N_3O_3SCl$ (-)

m/z: 362.0303 (+) (Na adduct) / 338.036 (-)

Retention time: 7.95+8.69 min (+) / 7.97+8.71 min (-)

Confidence level: 3

Proposed chemical structure:

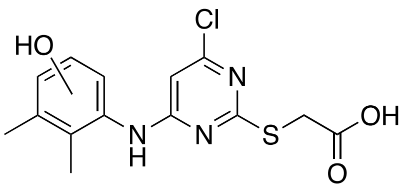

XIC:

Positive mode:

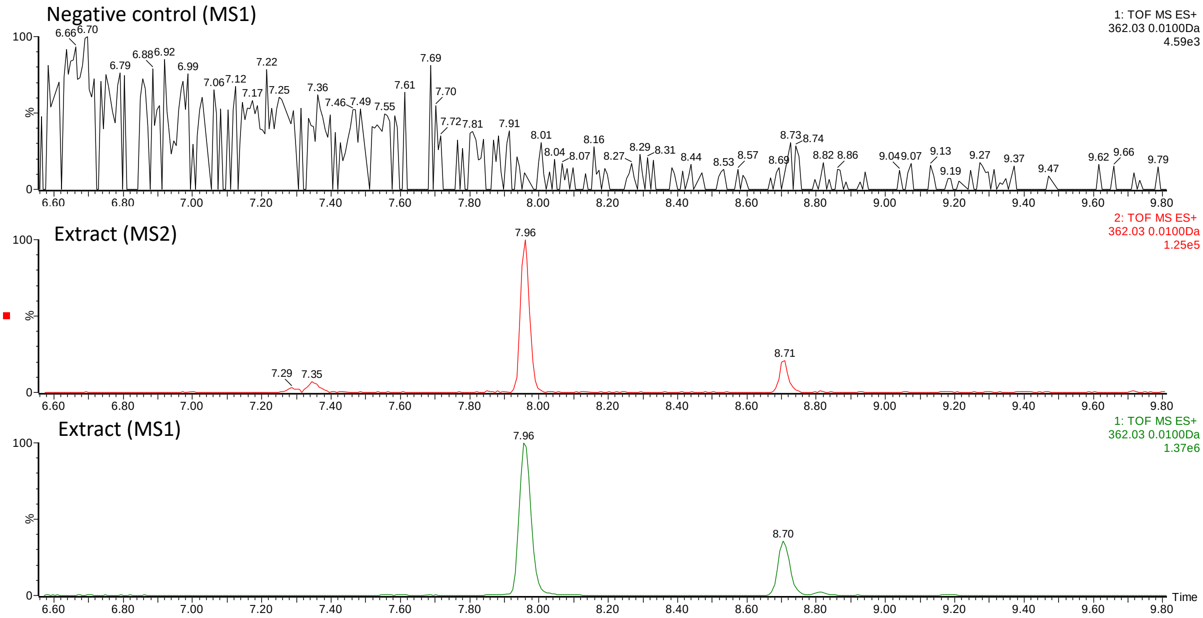

MS/MS spectrum:

Positive mode:

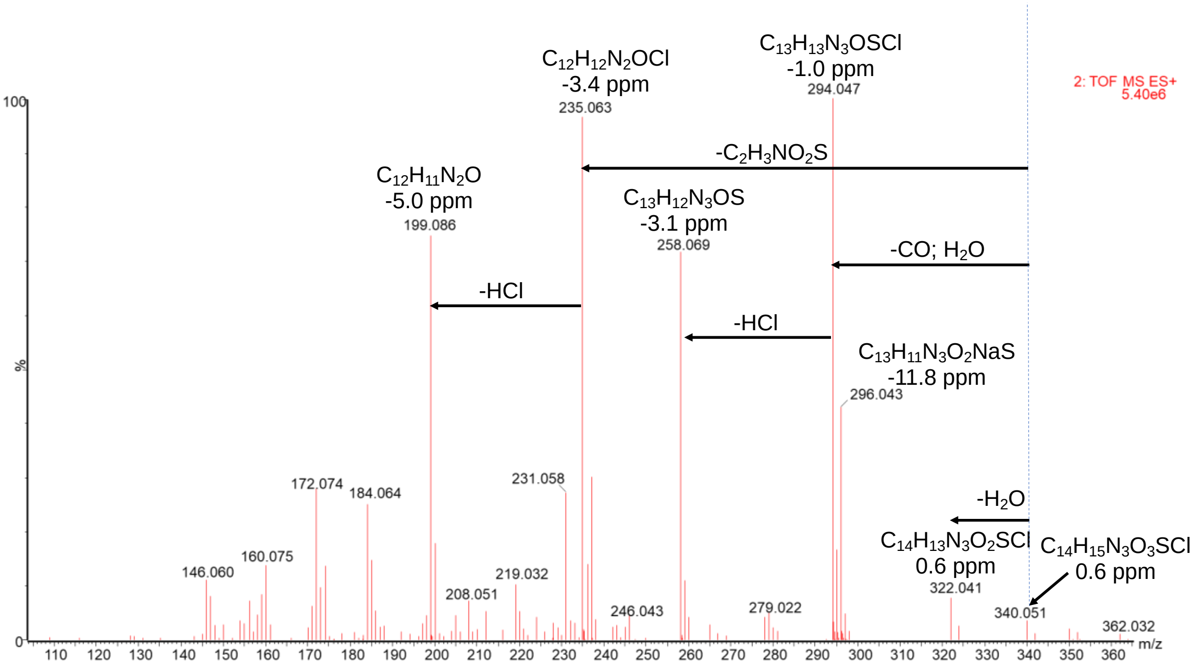

Compound ID: PA_3

Compound name: PA+Me

Chemical formula: $C_{15}H_{17}N_3O_2SCl$ (+) / $C_{15}H_{15}N_3O_2SCl$ (-)

m/z: 338.0718 (+) / 336.0578 (-)

Retention time: 10.57 min (+) / 10.59 (-)

Confidence level: 3

Proposed chemical structure:

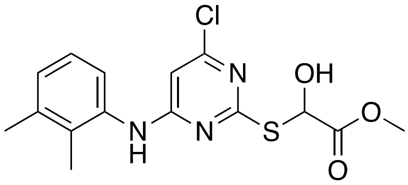

XIC:

Positive mode:

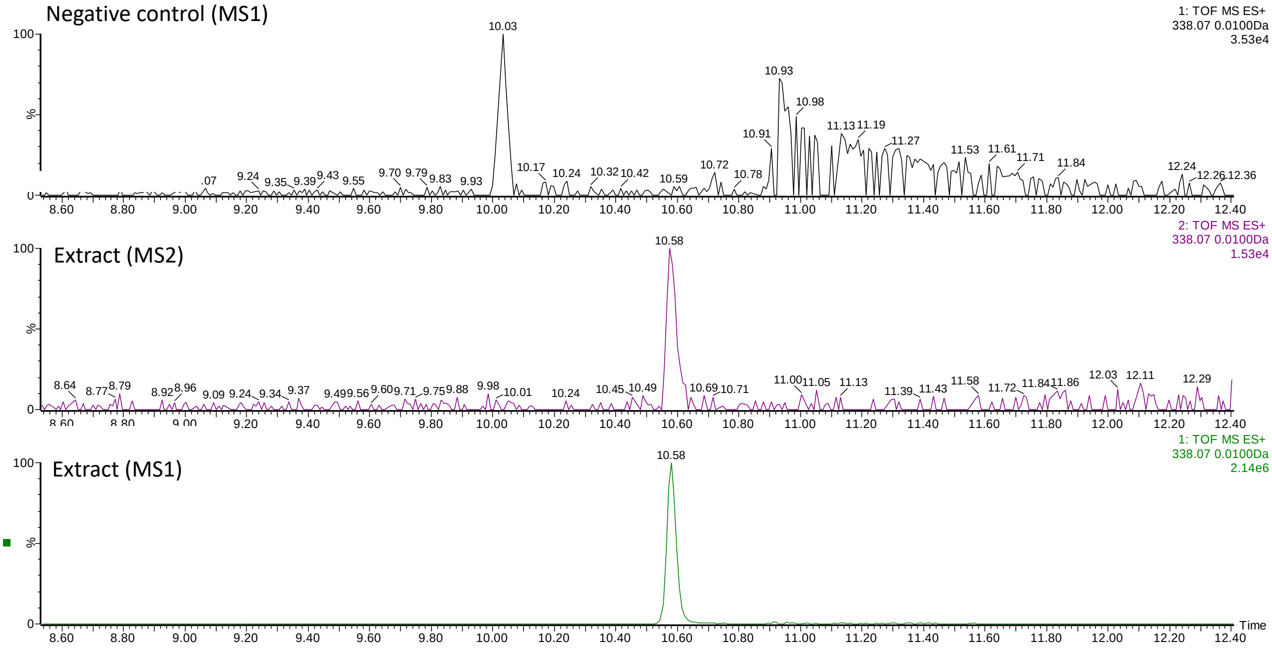

MS/MS spectrum:

Positive mode:

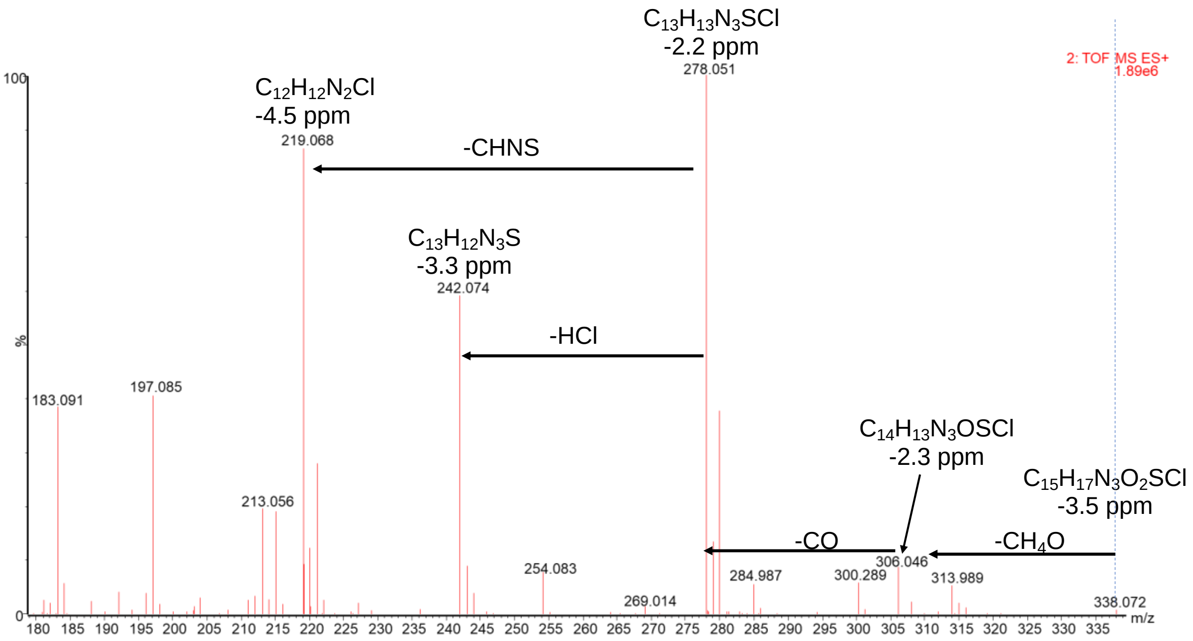

Compound ID: PA_4

Compound name: PA+Taurine

Chemical formula: $C_{16}H_{20}N_4O_4S_2Cl$ (+) / $C_{16}H_{18}N_4O_4S_2Cl$ (-)

m/z: 431.0606 (+)/ 429.047 (-)

Retention time: 9.64 min (+) / 9.67 (-)

Confidence level: 3

Proposed chemical structure:

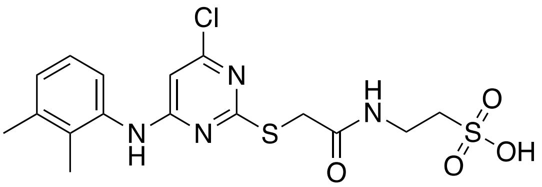

XIC:

Positive mode:

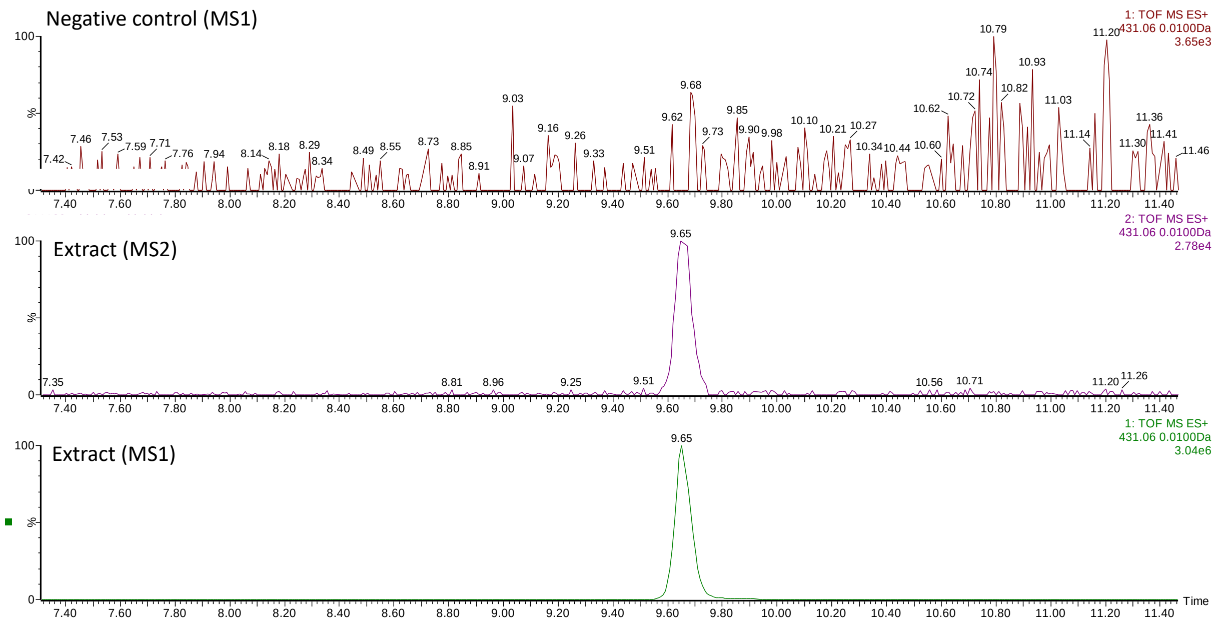

Negative mode:

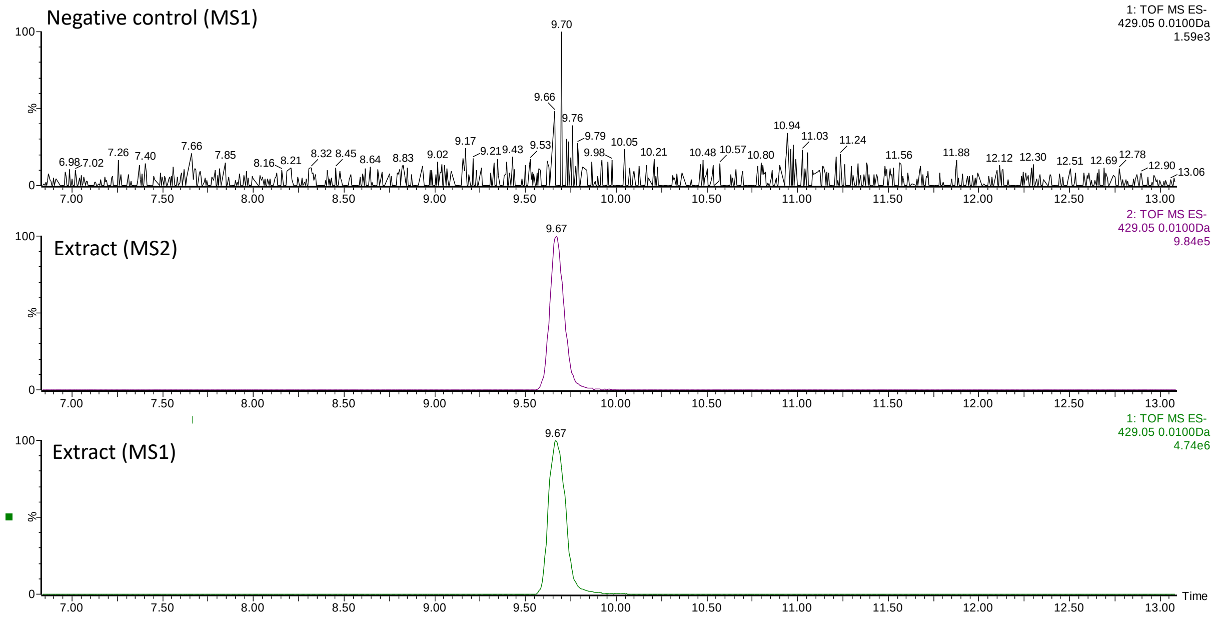

MS/MS spectrum:

Positive mode:

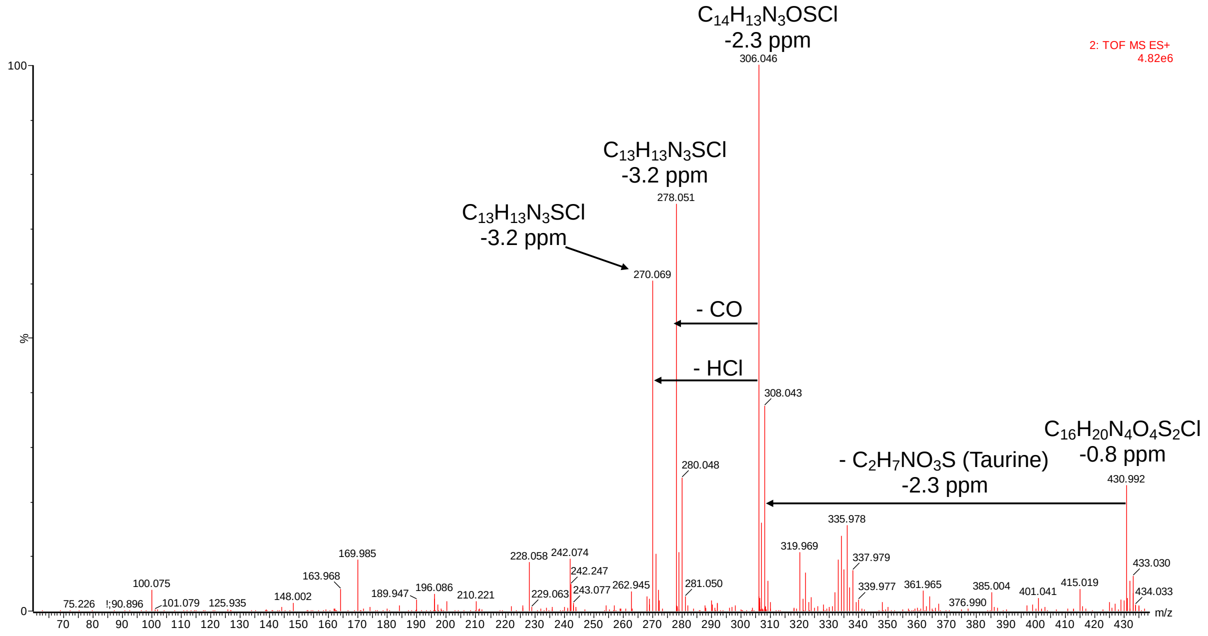

Negative mode:

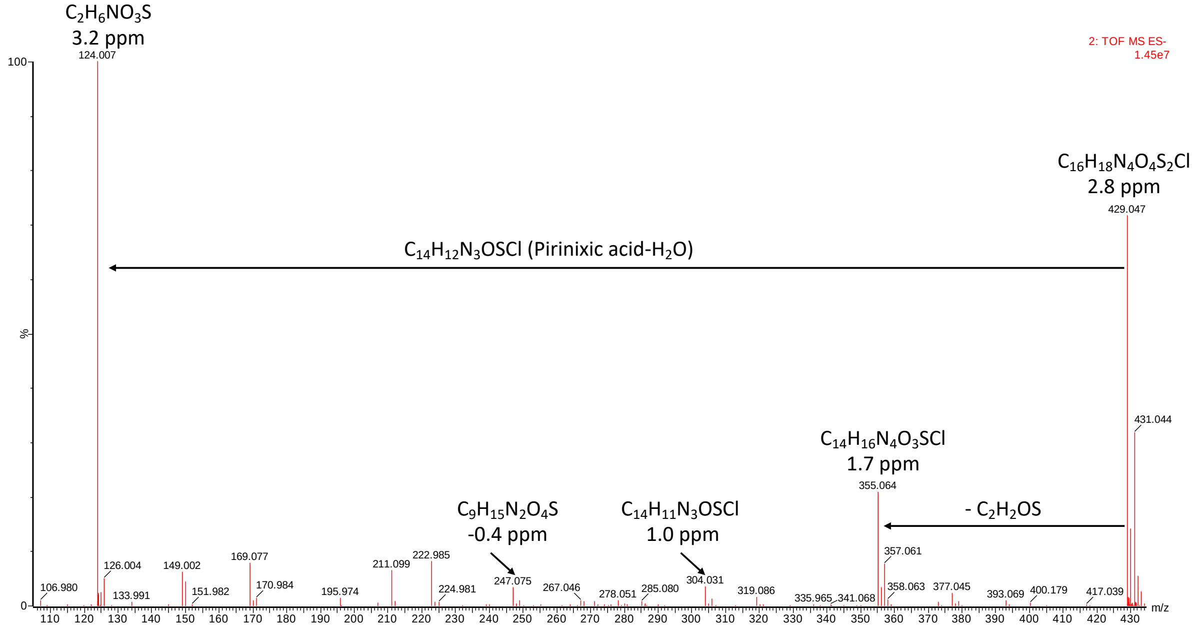

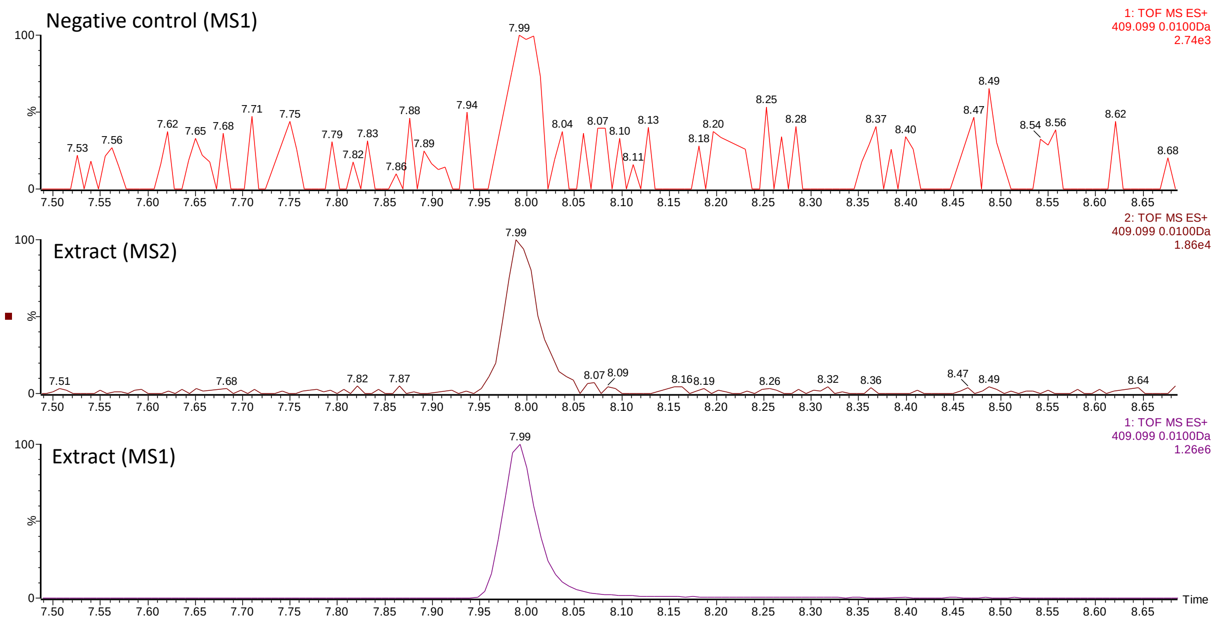

Compound ID: PA_5

Compound name: PA+SO3

Chemical formula: $C_{14}H_{13}ClN_3O_6S_2$ (-)

m/z: 417.9930 (-)

Retention time: 7.82+7.89 min (-)

Confidence level: 4

Proposed chemical structure:

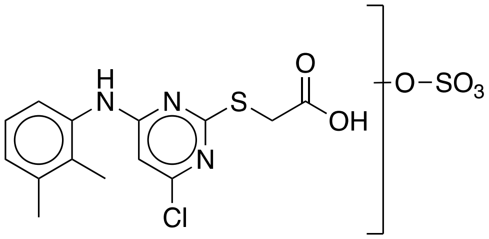

XIC:

Negative mode

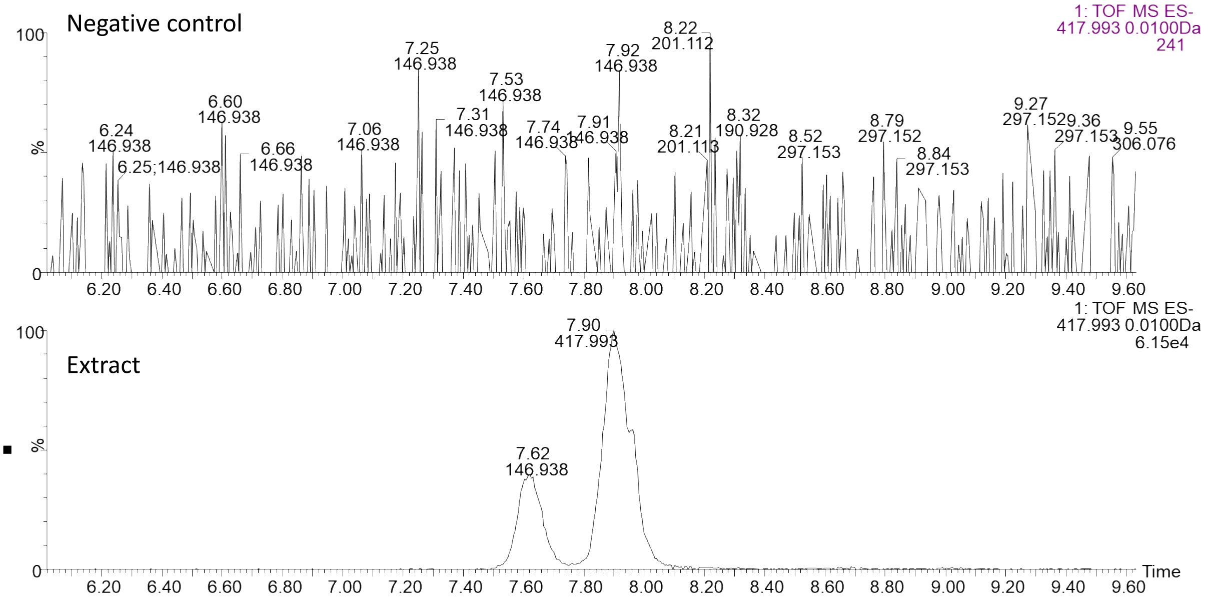

Compound ID: PA_6

Compound name: PA +Cys+O

Chemical formula: $C_{16}H_{18}N_4O_5S_2Cl$ (-)

m/z: 445.0410 (-)

Retention time: 7.30+8.26 min (-)

Confidence level: 4

Proposed chemical structure:

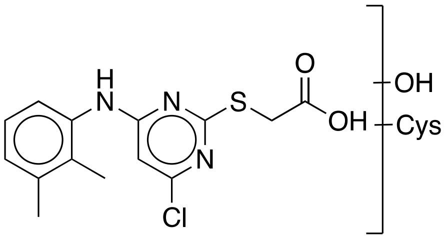

XIC:

Negative mode

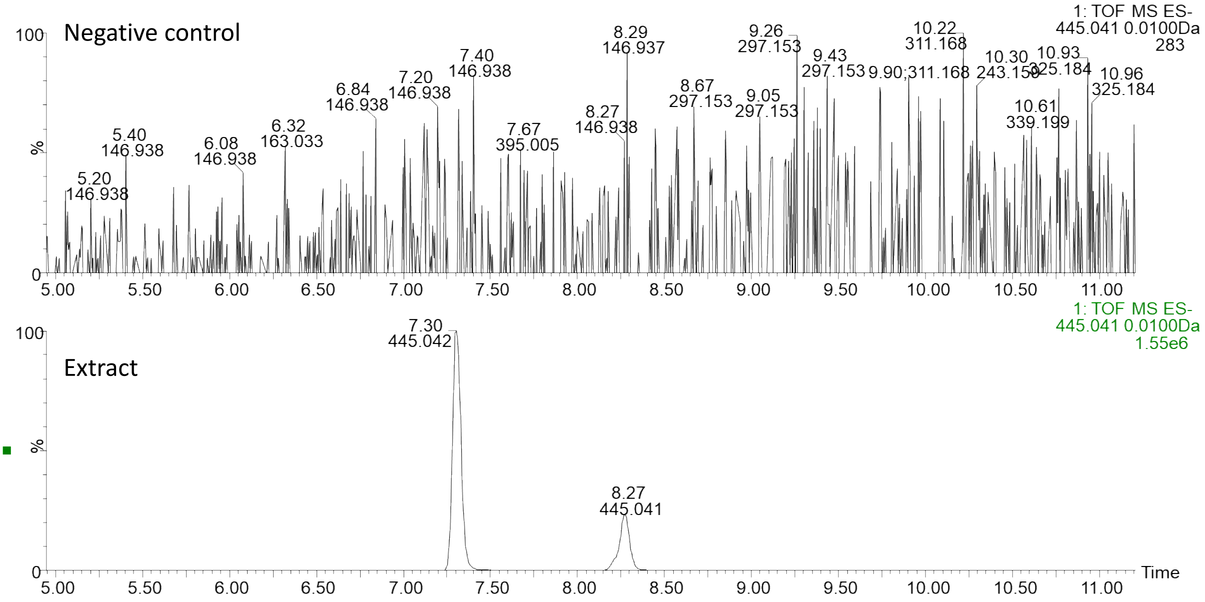

MS/MS Spectrum:

Negative mode

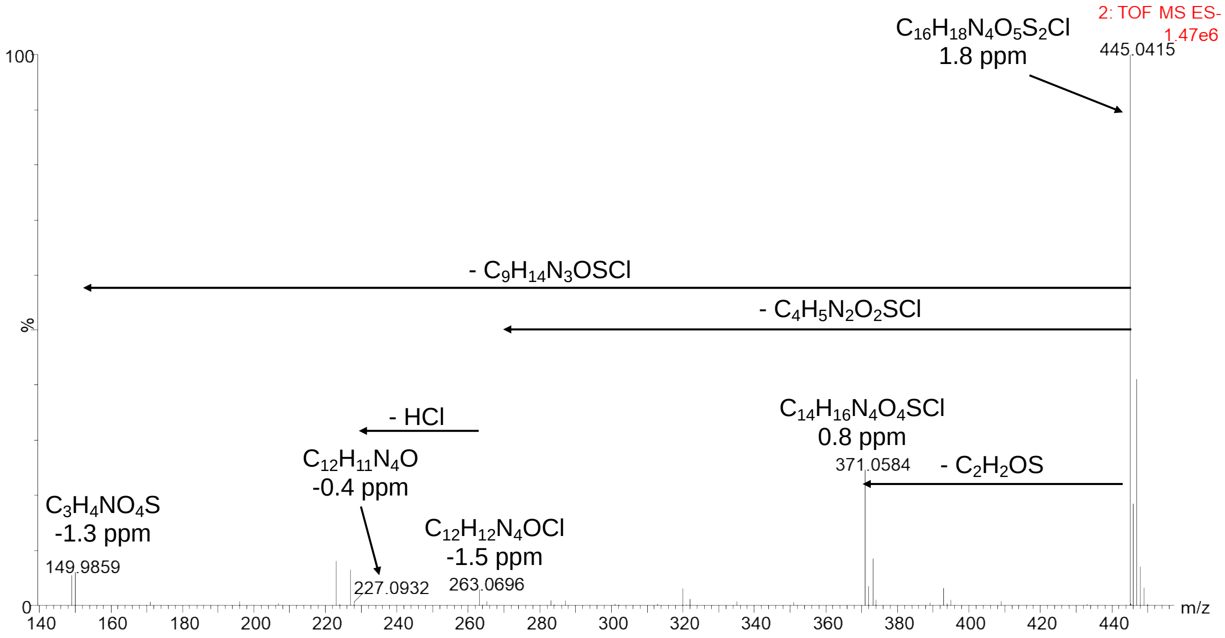

10.2 TPs of atorvastatin

Compound ID: AT_0

Compound name: Atorvastatin (Parental compound)

Chemical formula: $C_{33}H_{36}N_2O_5F$ (+) / $C_{33}H_{34}N_2O_5F$ (-)

m/z: 559.2613 (+) / 557.249 (-)

Retention time: 6.1 min

Confidence level: 1

Proposed chemical structure:

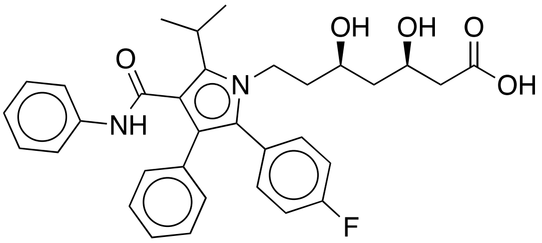

XIC:

Positive mode:

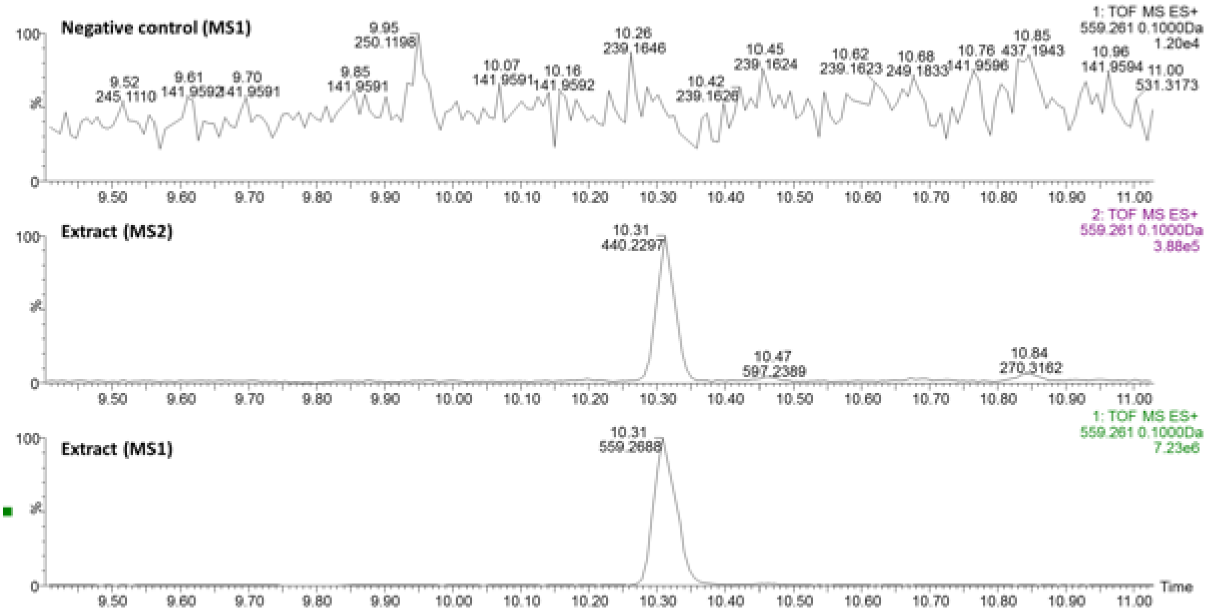

Negative mode:

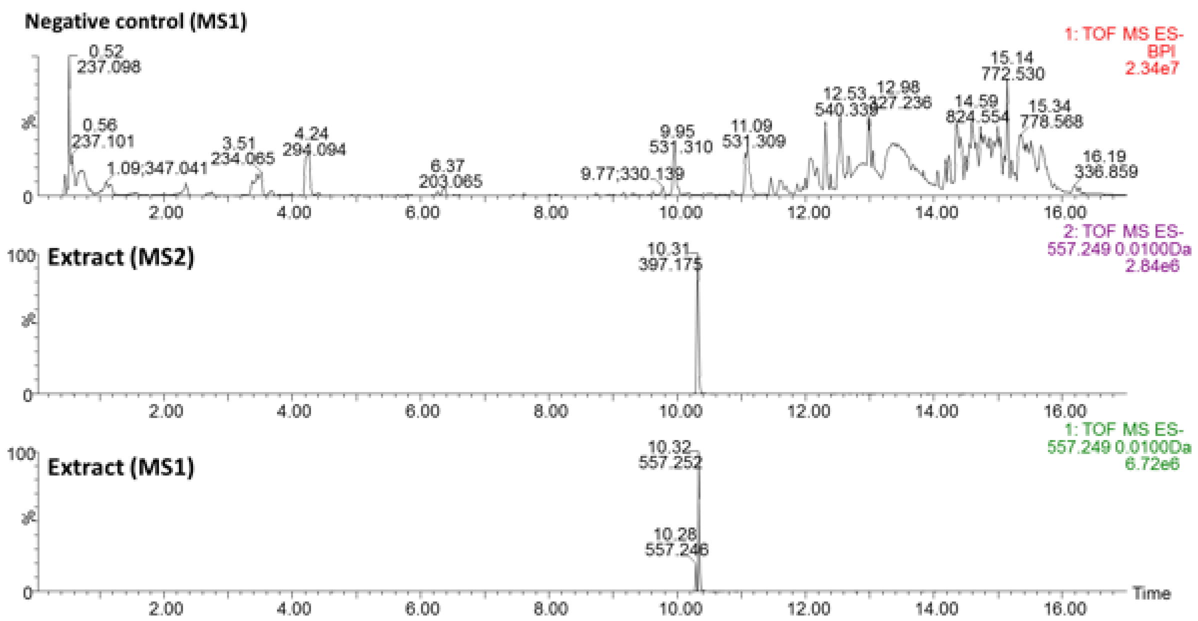

MS/MS spectrum:

Positive mode:

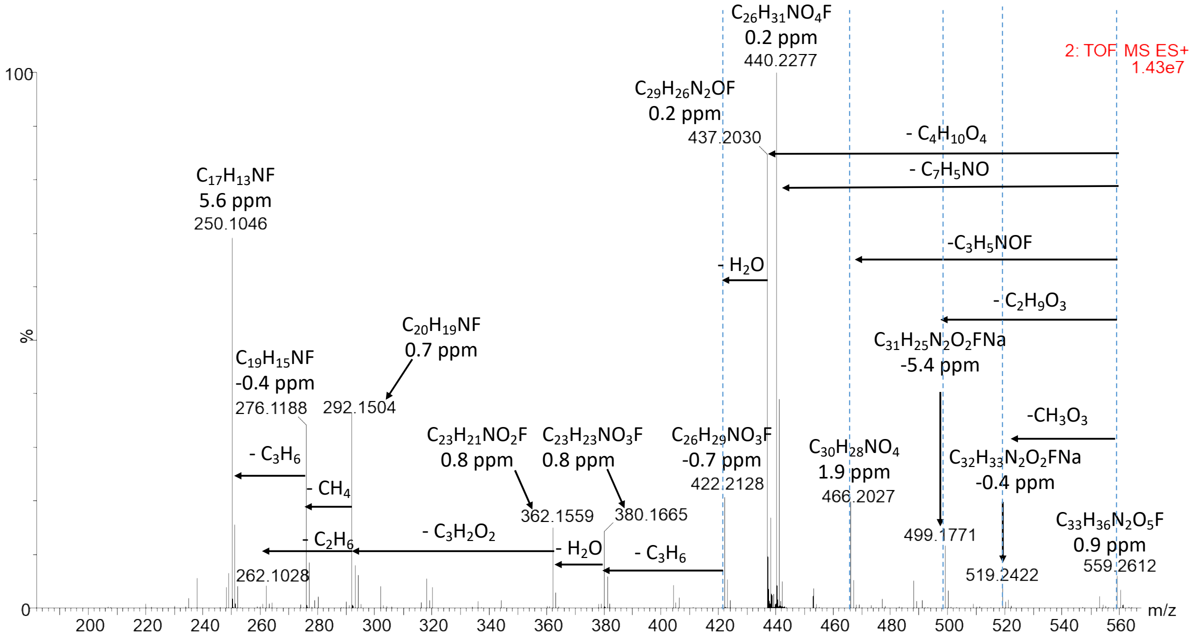

Compound ID: AT_1

Compound name: Hydroxy-Atorvastatin (AT+O)

Chemical formula: $C_{33}H_{35}N_2O_6FNa$ (+) / $C_{33}H_{34}N_2O_6F$ (-)

m/z: 597.239 (+) (Na adduct) / 573.2406 (-)

Retention time: 9.2+10.21+10.46 min(+) / 9.20+10.2+10.46min (-)

Confidence level: 3

Proposed chemical structure:

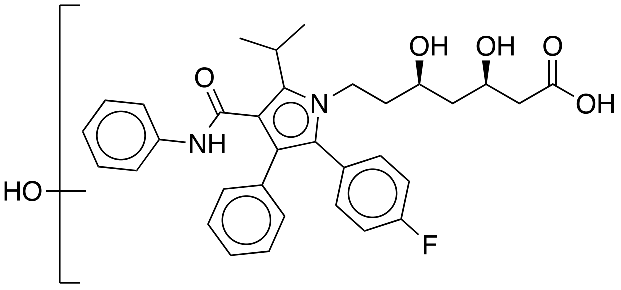

XIC:

Negative mode:

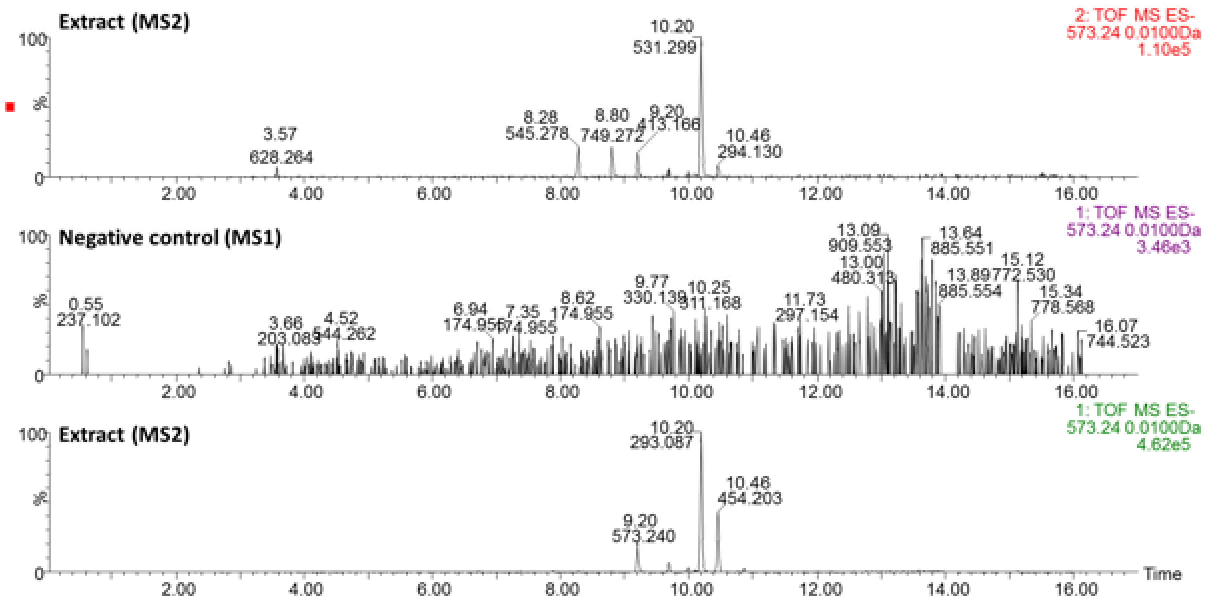

MS/MS spectrum:

Negative mode:

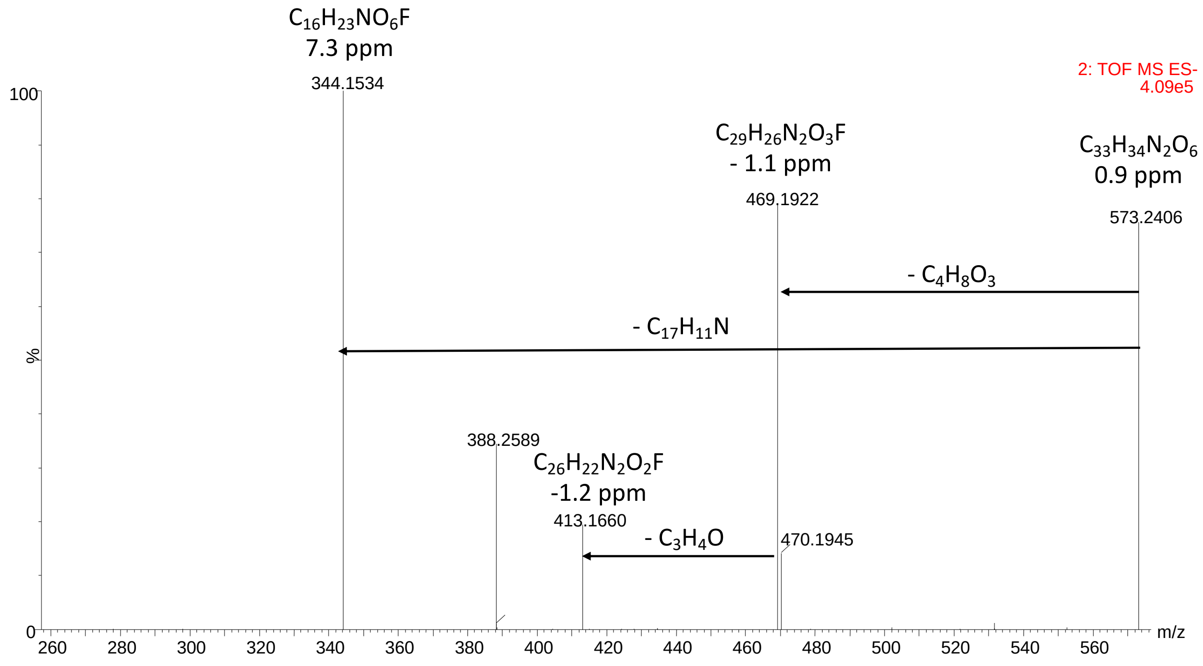

Compound ID: AT_2

Compound name: Atorvastine+O+Glucuronide (AT+O+Gluc)

Chemical formula: $C_{39}H_{44}N_2O_{12}F$ (+) / $C_{39}H_{42}N_2O_{12}F$ (-)

m/z: 751.2900 (+) / 749.272 (-)

Retention time: 8.28+8.81min (+) / 8.28+8.80

Confidence level: 3

Proposed chemical structure:

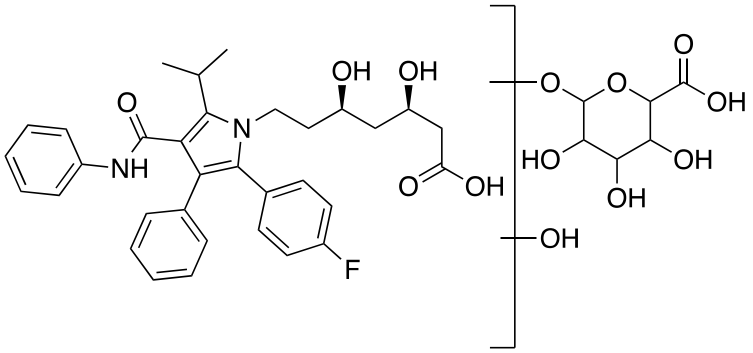

XIC:

Negative mode

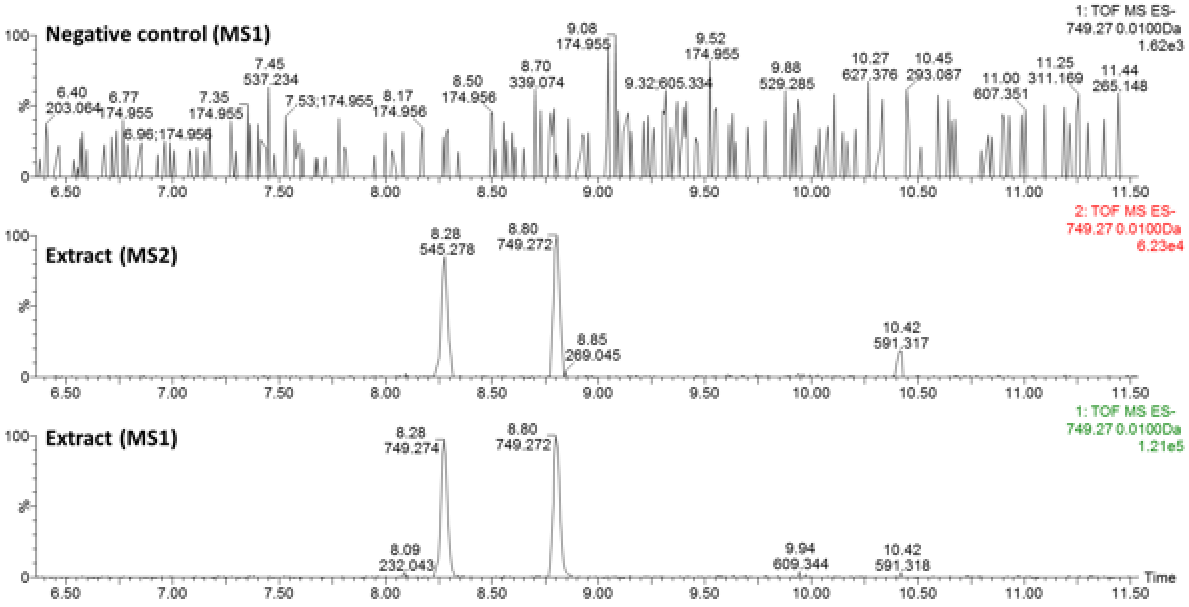

MS/MS spectrum:

Negative mode:

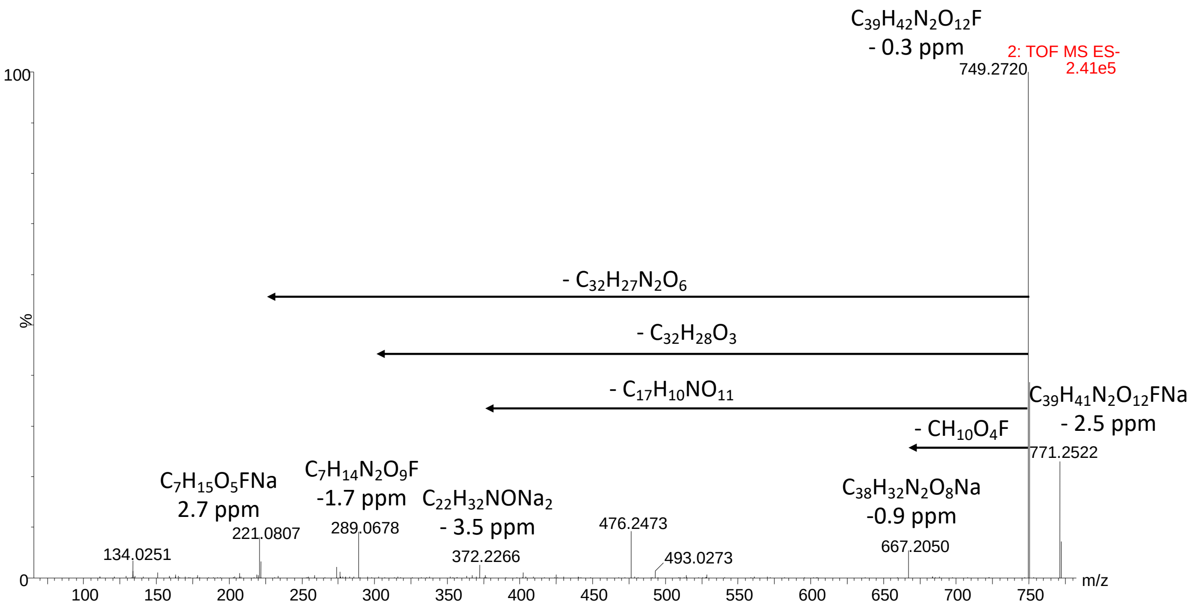

Compound ID: AT_3

Compound name: Atorvastatin – 2 H_2O

Chemical formula: $C_{33}H_{31}N_2O_3FNa$ (+)

m/z: 545.2220 (+)

Retention time: 9.94+10.78 min (+)

Confidence level: 3

Proposed chemical structure:

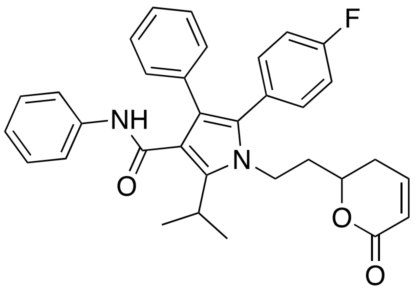

XIC:

Positive mode:

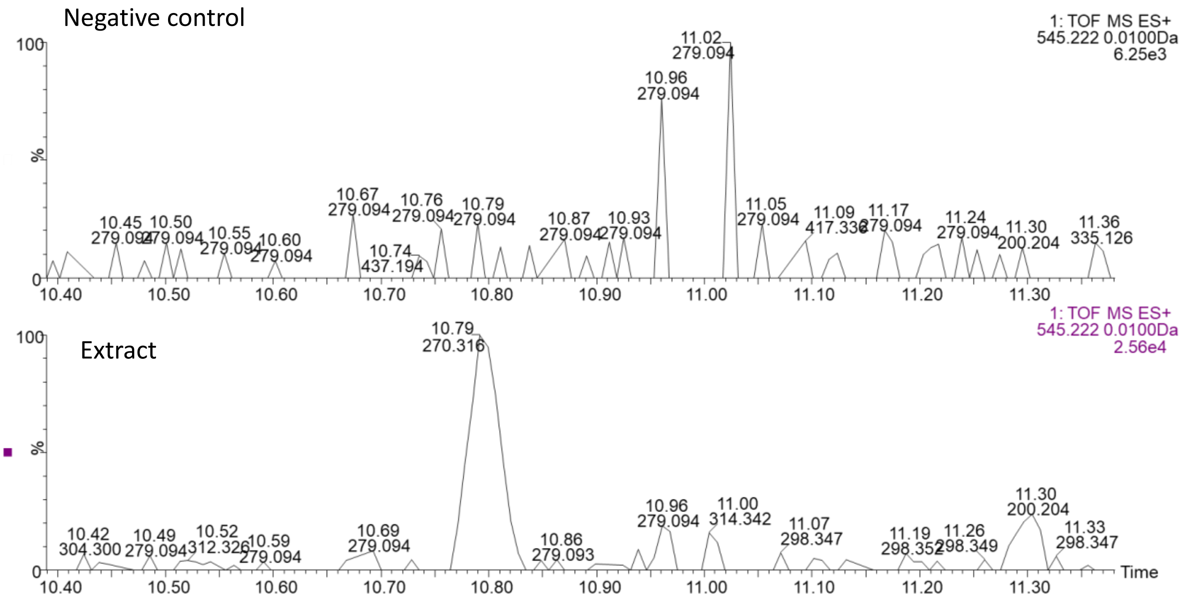

MS/MS spectrum

Positive mode:

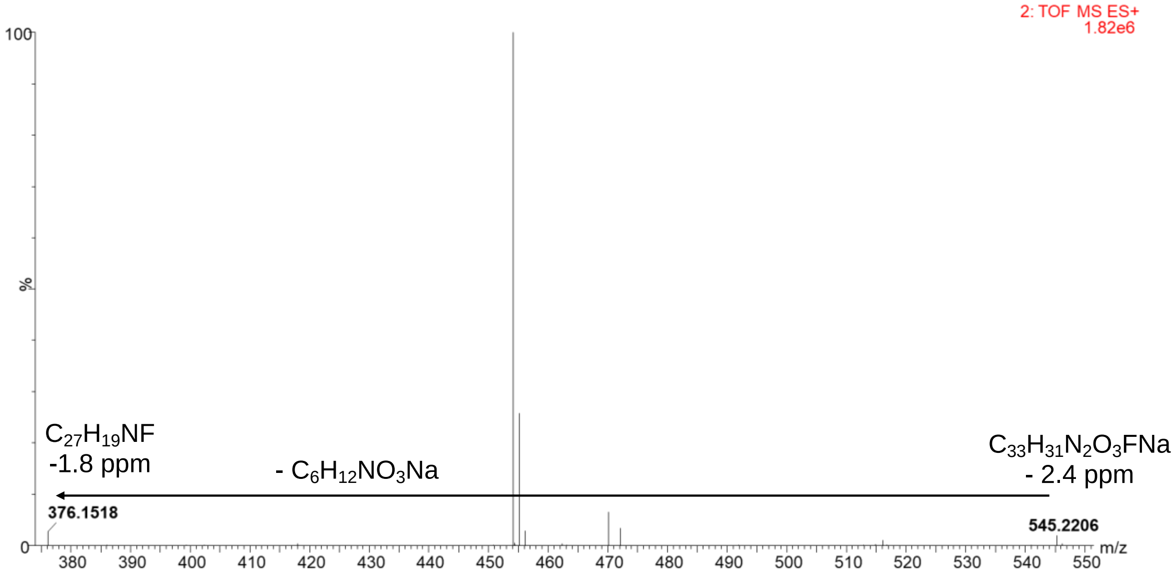

Compound ID: AT_4

Compound name: Atorvastatin + Cysteine

Chemical formula: $C_{35}H_{39}N_3O_6FS$ (-)

m/z: 648.2540 (-)

Retention time: 10.00 min (-)

Confidence level: 3

Proposed chemical structure:

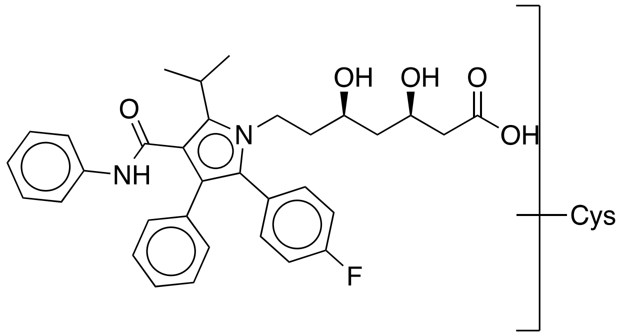

XIC:

Negative mode

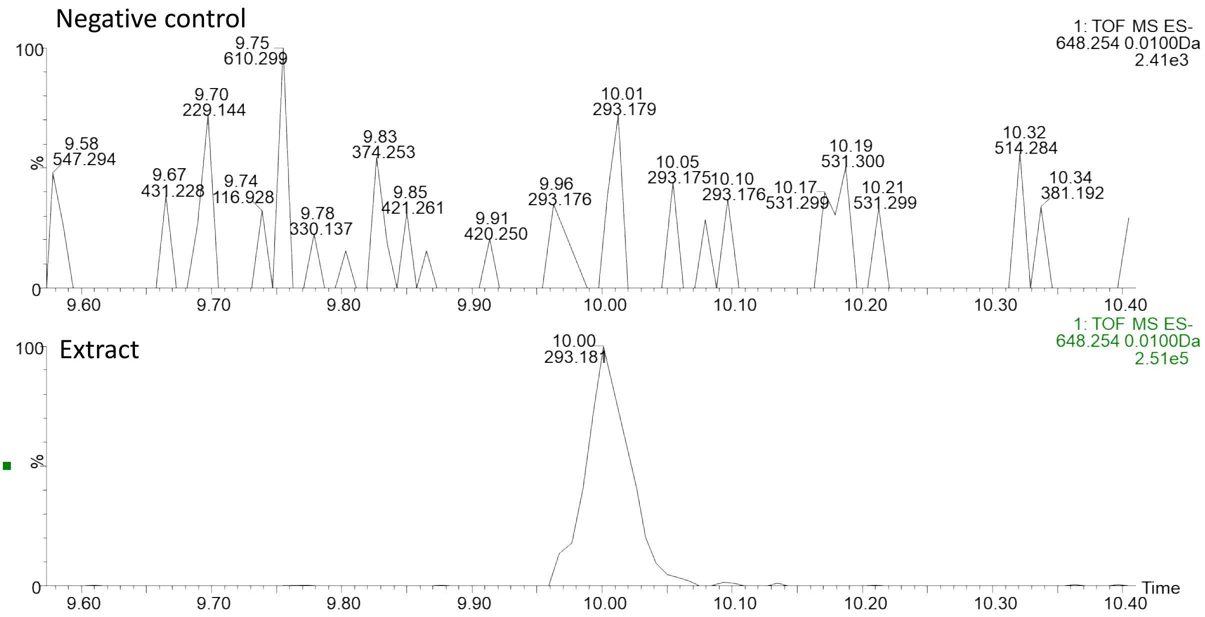

MS/MS spectrum:

Negative mode

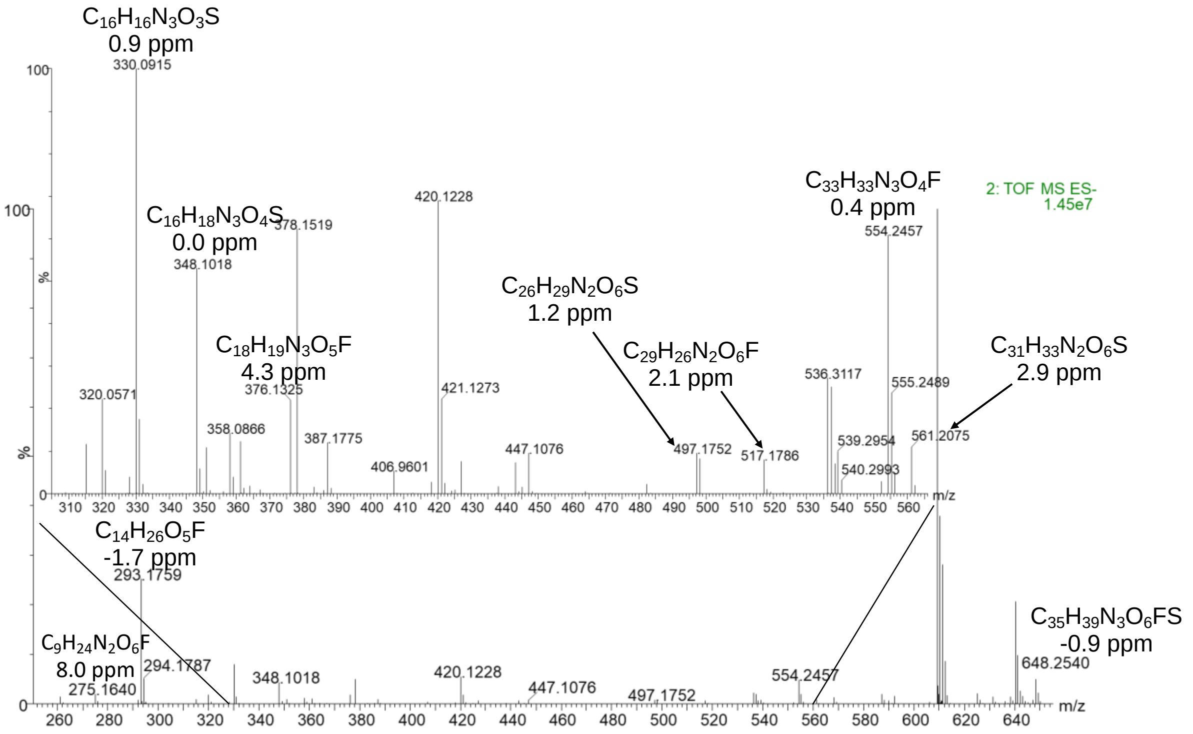

Compound ID: AT_5

Compound name: unidentified TP

Chemical formula: $C_{35}H_{39}N_3O_6FS$ (-)

m/z: 629.219 (-)

Retention time: 9.69 min (-)

Confidence level: 5

Proposed chemical structure:

XIC:

Negative mode

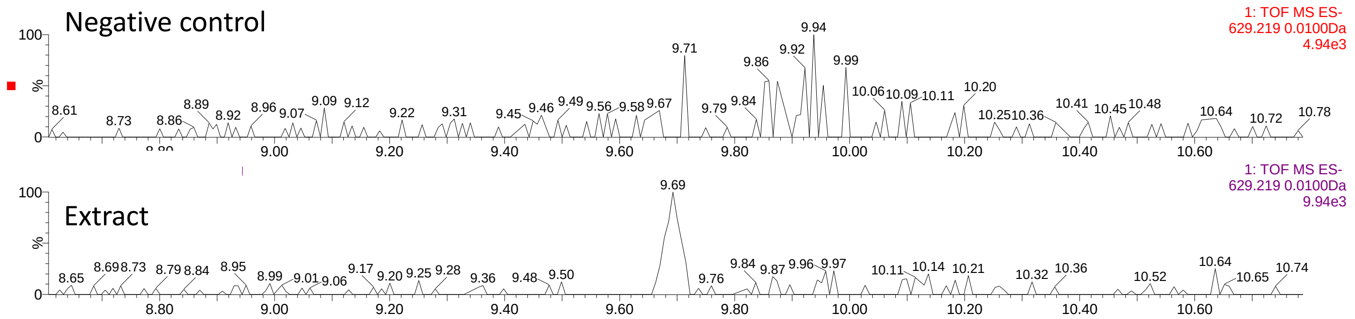

MS-spectrum:

Negative mode

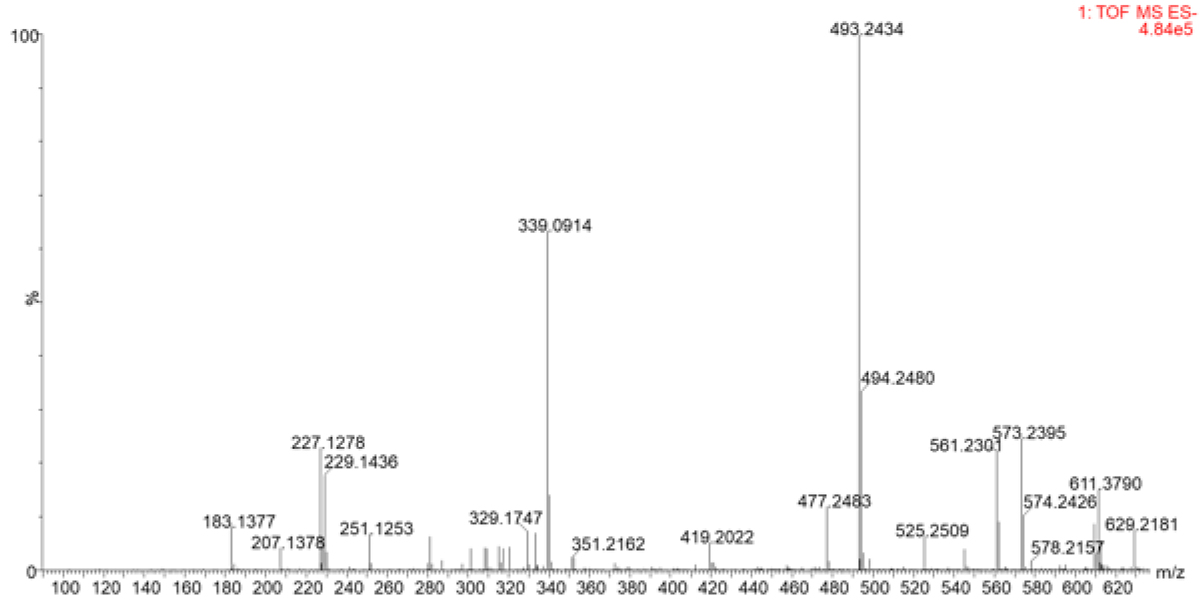

Compound ID: AT_6

Compound name: Atorvastatin+O+CH2

Chemical formula: $C_{34}H_{37}N_{2}O_{6}FNa$ (+)

m/z: 611.2525 (+)

Retention time: 9.67+10.84 min (+)

Confidence level: 3

Proposed chemical structure:

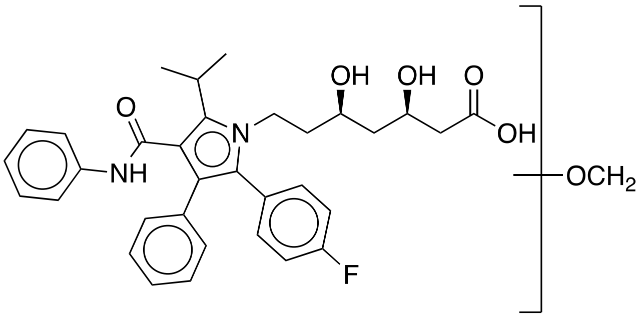

XIC:

Positive mode

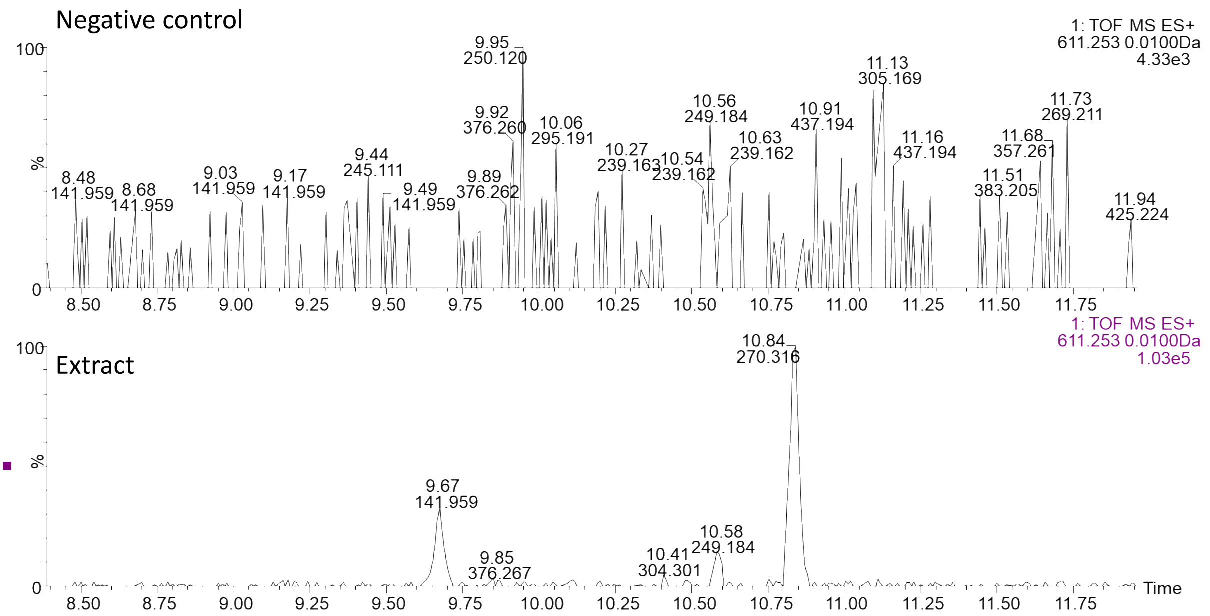

MS/MS spectrum:

Positive mode

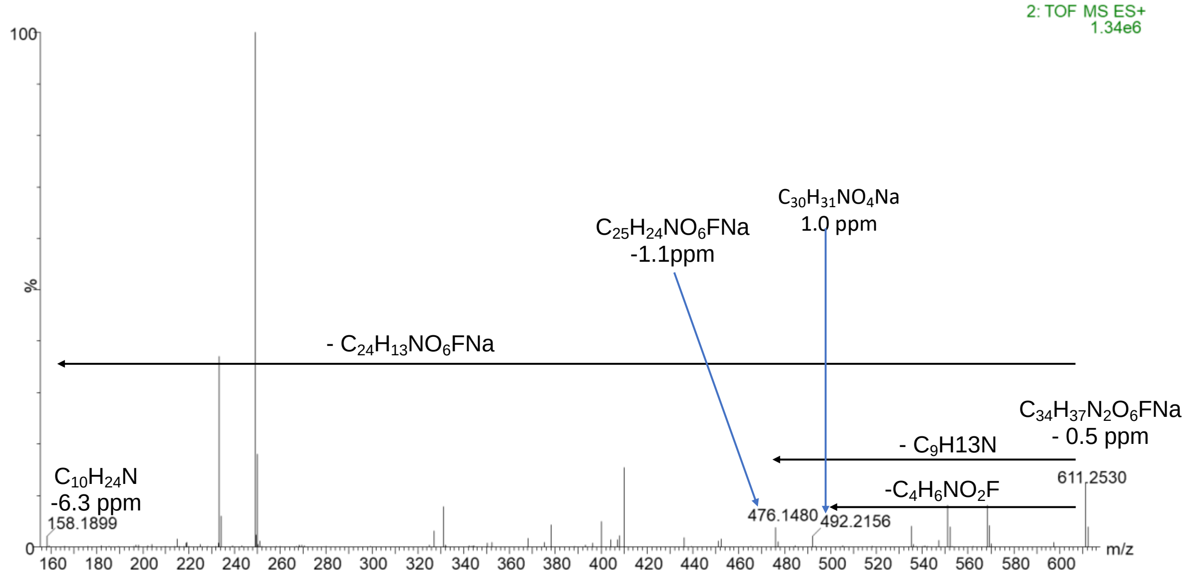

10.3 TPs of Genistein

Compound ID: Gen_0

Compound name: Genistein (Parental compound)

Chemical formula: $C_{15}H_{11}O_5$ (+) / $C_{15}H_9O_5$ (-)

m/z: 271.061 (+) / 269.046 (-)

Retention time: 8.31 min (+) / 8.30 min (-)

Confidence level: 3

Proposed chemical structure:

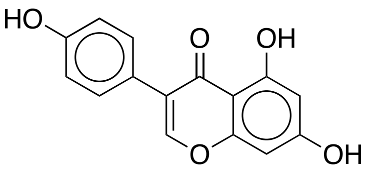

XIC:

Positive mode:

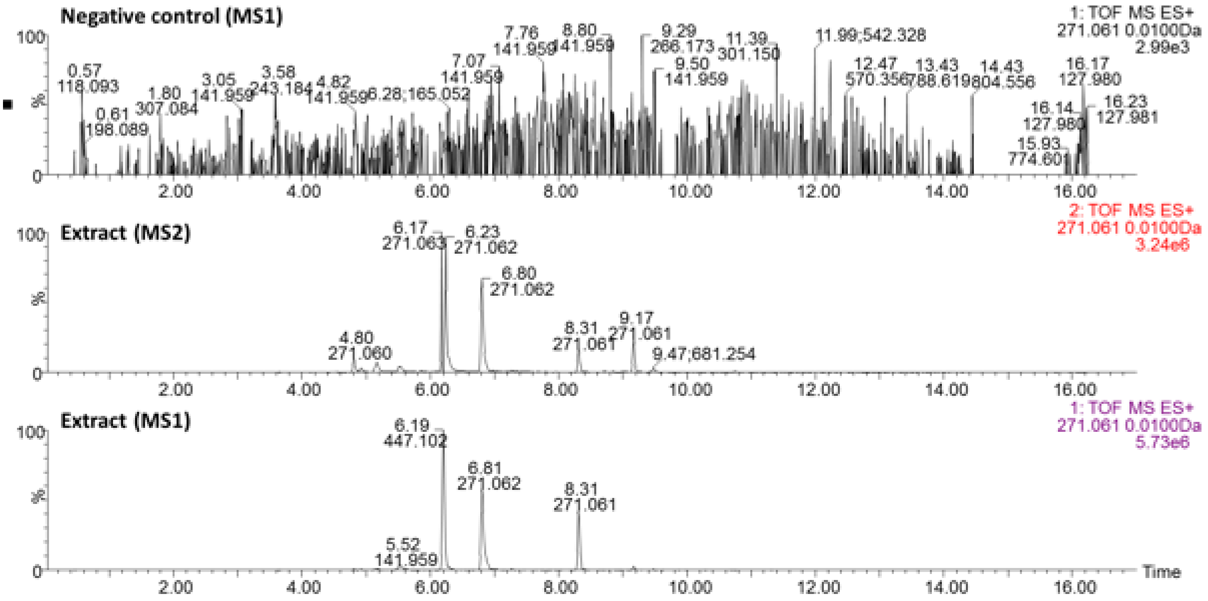

MS/MS spectrum:

Positive mode:

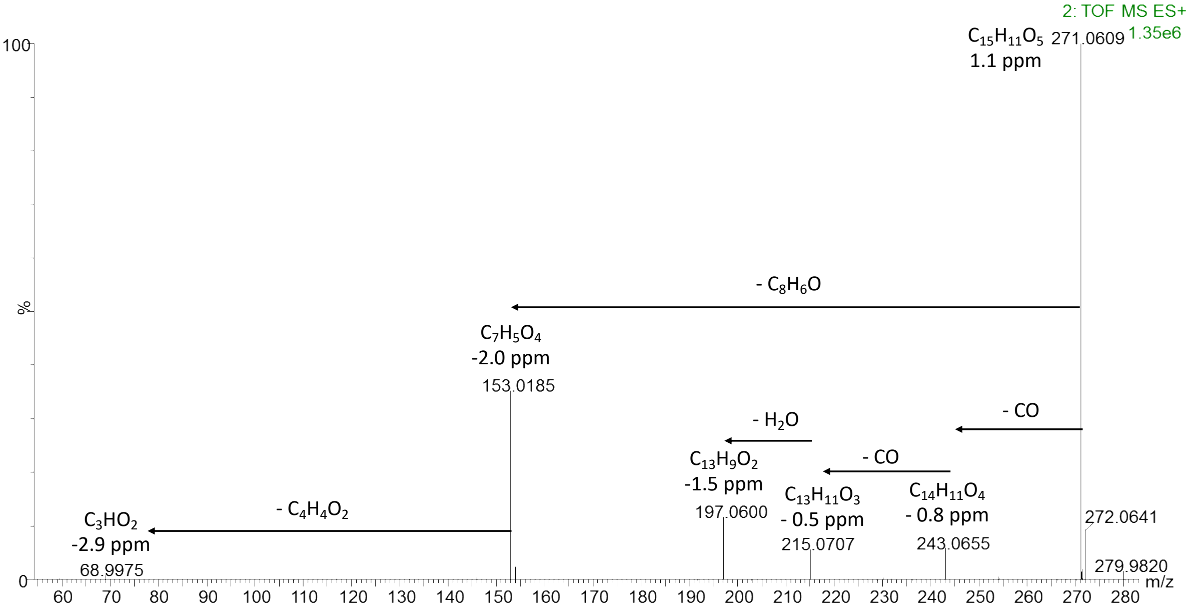

Compound ID: Gen_1

Compound name: Genistein+Glucuronide (Gen+Gluc)

Chemical formula: $C_{21}H_{18}O_{11}Na$ (+) / $C_{21}H_{16}O_{11}Na$ (-)

m/z: 469.075 (+) / 467.0601 (-)

Retention time: 6.19+6.23+6.80 min (+) / 6.19+6.23+6.79 min (-)

Confidence level: 3

Proposed chemical structure:

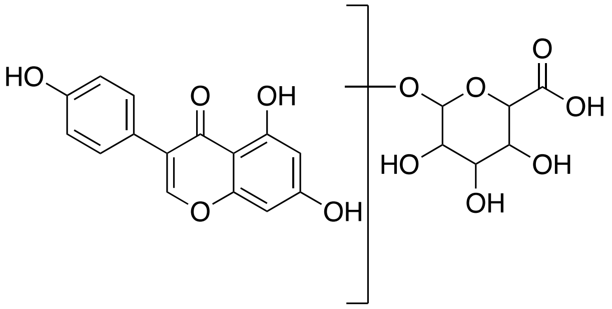

XIC:

Positive mode:

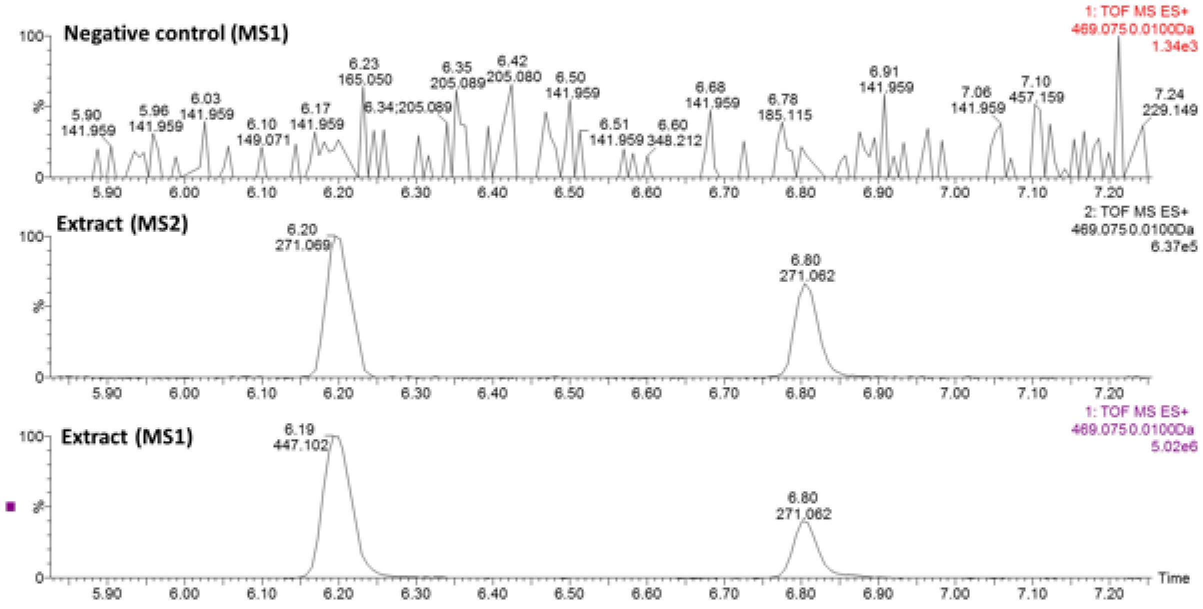

Negative mode:

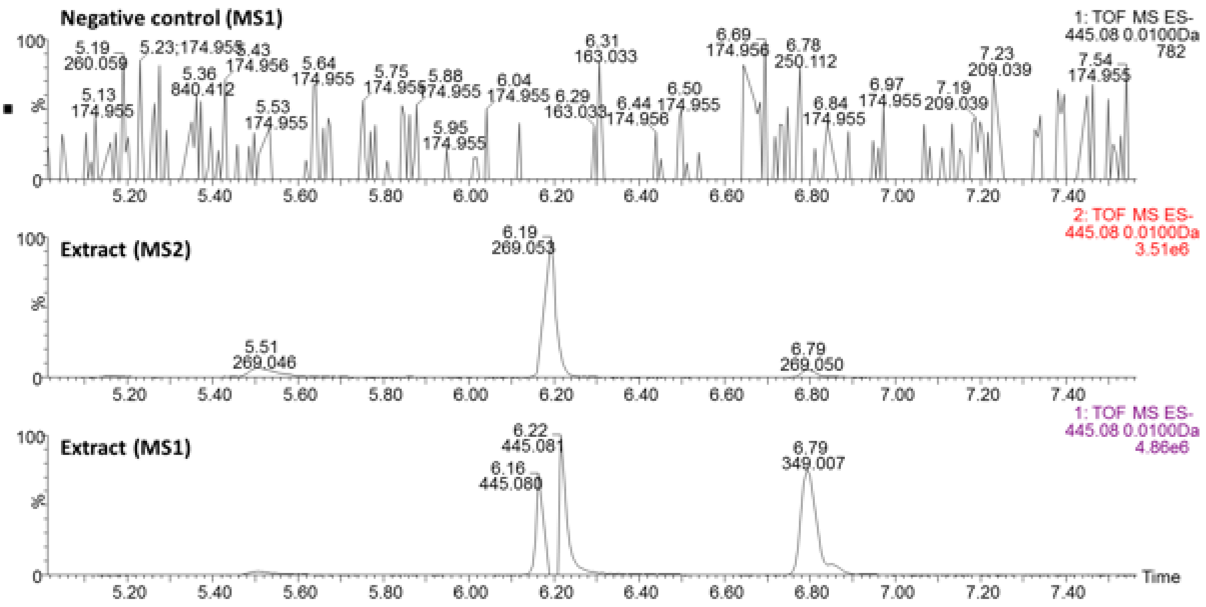

MS/MS spectrum:

Positive mode:

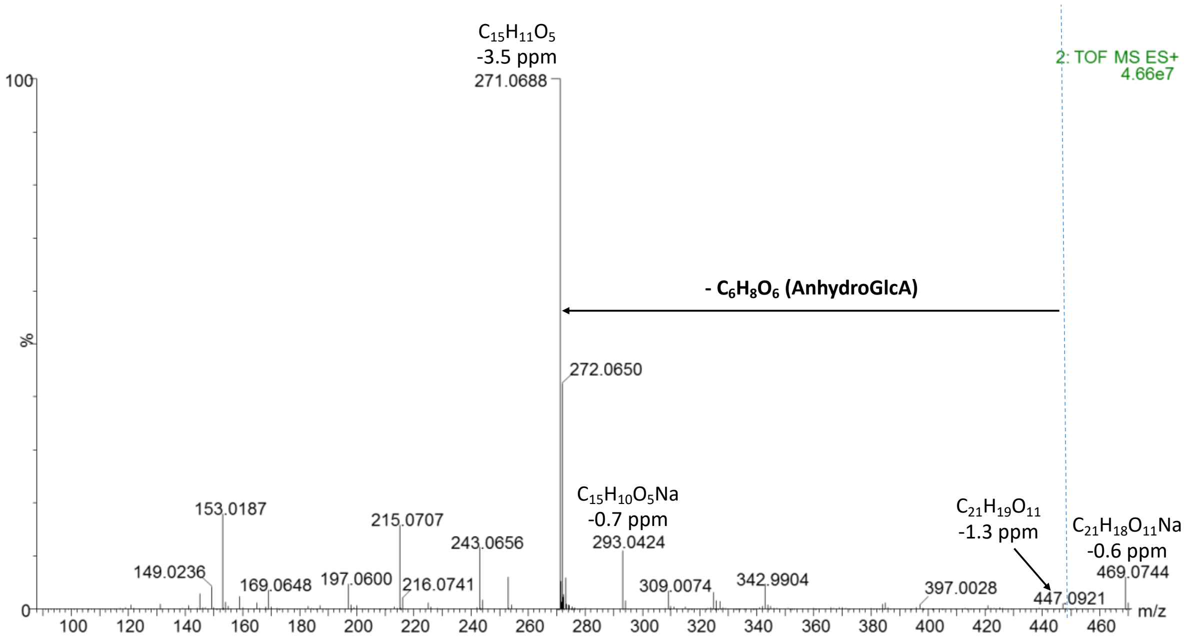

Compound ID: Gen_2

Compound name: Genistein+Glucuronide+SO3+H2O+CH3O2

Chemical formula:

m/z: 621.1100 (-)

Retention time: 4.80+5.16 min (-)

Confidence level: 3

Proposed chemical structure:

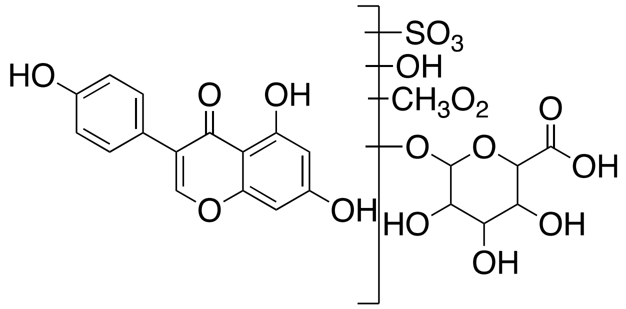

XIC:

Negative mode

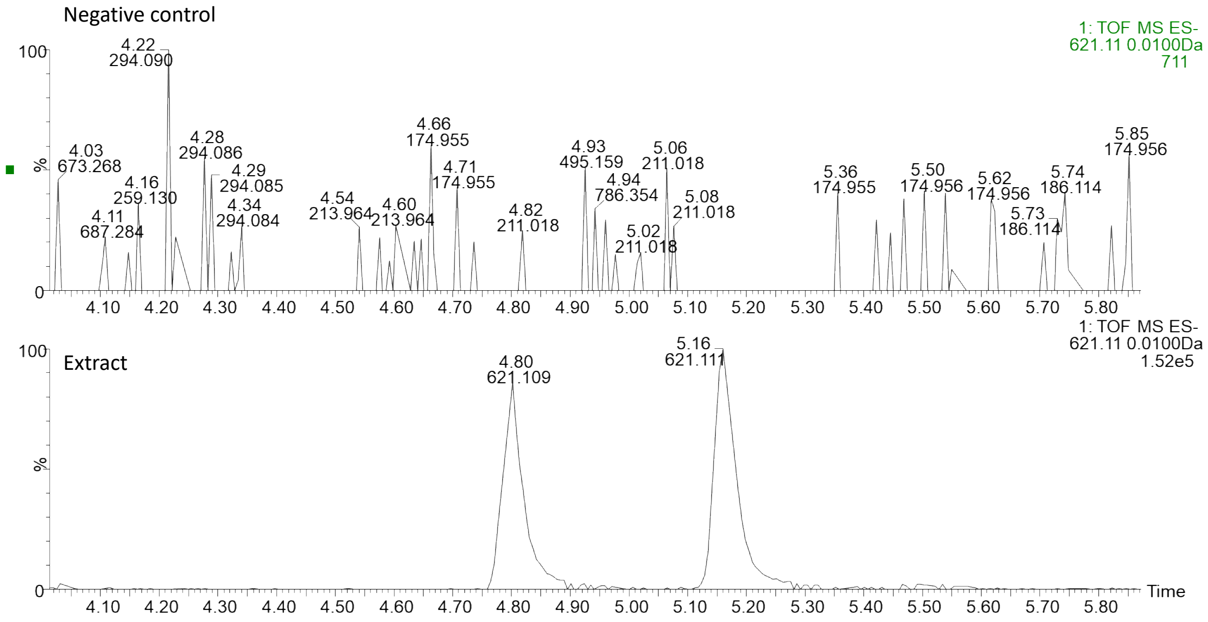

MS/MS spectrum:

Negative mode:

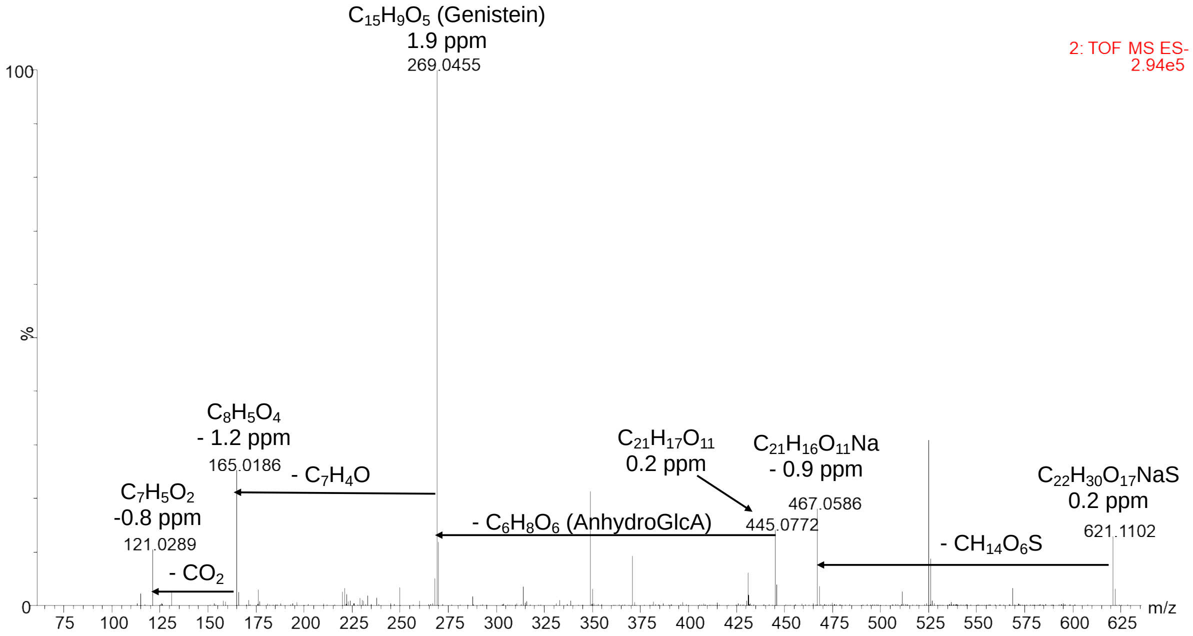

Compound ID: Gen_3

Compound name: Genistein+Glucuronide+SO3

Chemical formula: $C_{21}H_{16}O_{14}NaS$ / $C_{21}H_{16}O_{14}S$

m/z: 547.0150 (Na adduct) / 525.0350

Retention time: 5.13 + 5.51 min (-)

Confidence level: 3

Proposed chemical structure:

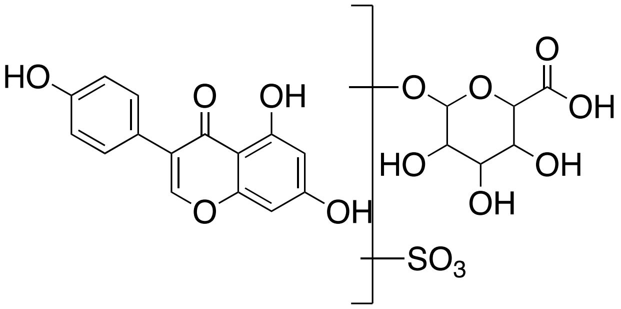

XIC:

Negative mode:

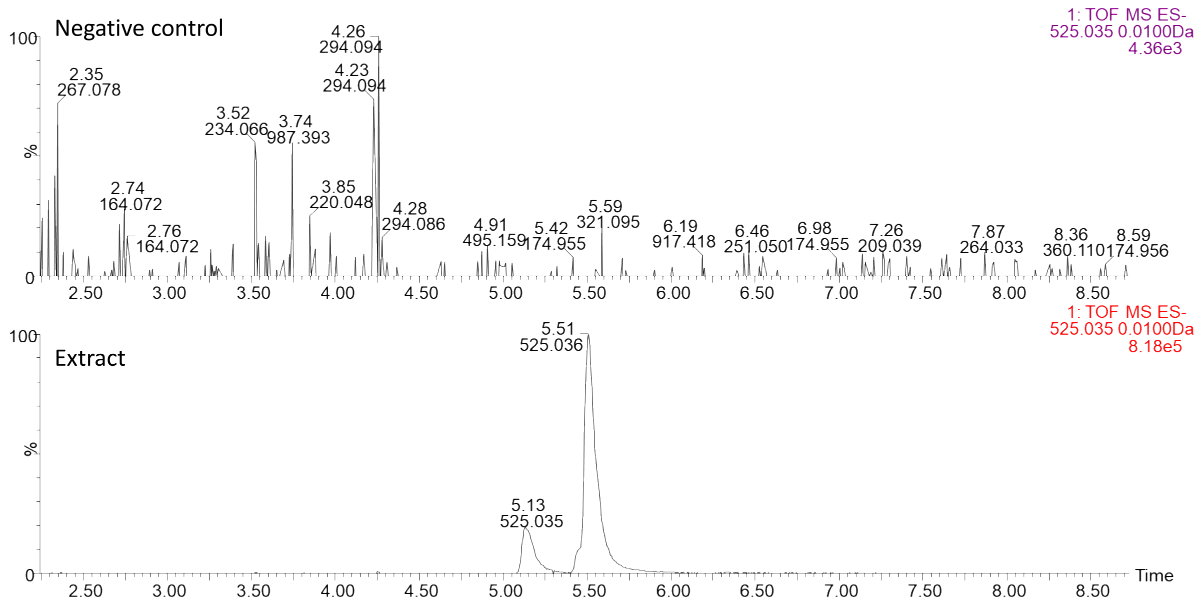

MS/MS spectrum:

Negative mode:

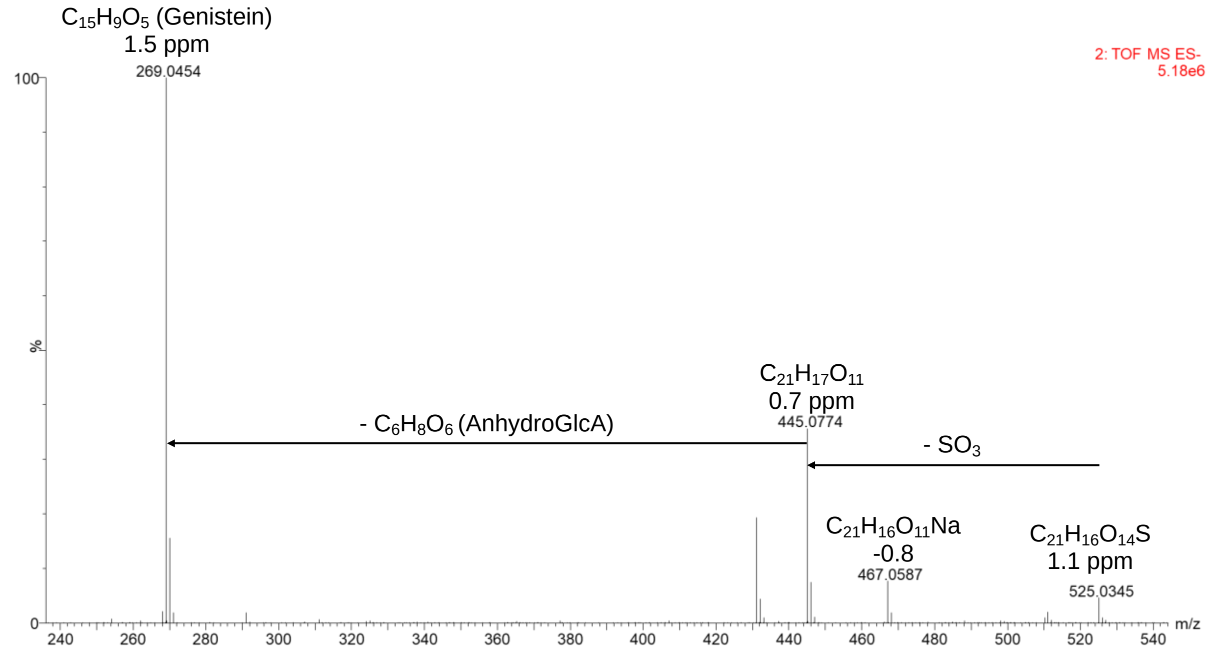

Compound ID: Gen_4

Compound name: Genistein + SO3 + CH2

Chemical formula: $C_{16}H_{11}O_9S$ (-)

m/z: 379.0130 (-)

Retention time: 6.35+6.48+6.92 min (-)

Confidence level: 3

Proposed chemical structure:

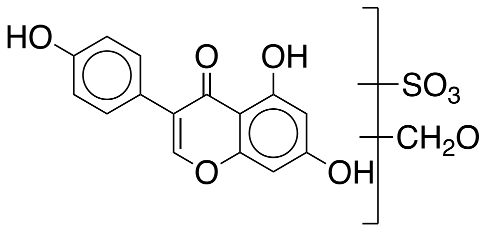

XIC:

Negative mode

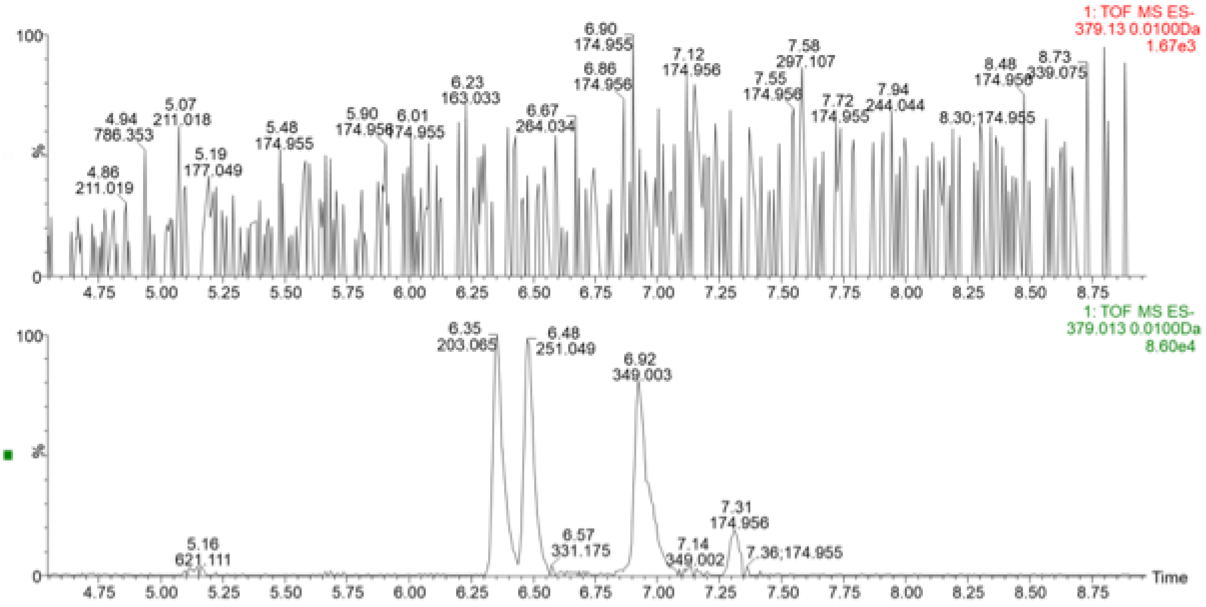

MS/MS spectrum:

Negative mode

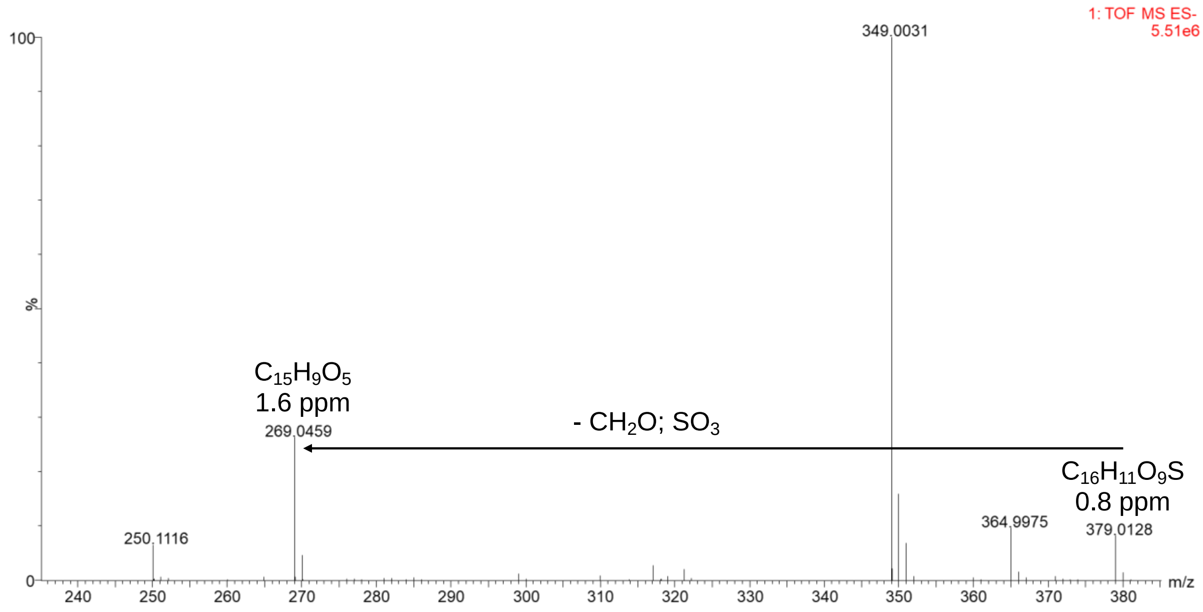

Compound ID: Gen_5

Compound name: Genistein + O + SO3

Chemical formula: $C_{16}H_{11}O_9S$ (-)

m/z: 364.9980 (-)

Retention time: 6.13+6.98 min (-)

Confidence level: 3

Proposed chemical structure:

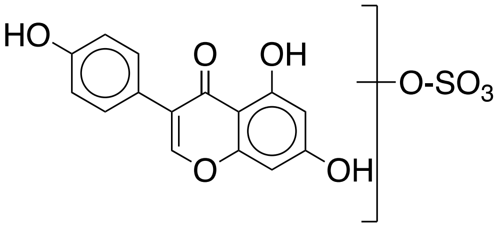

XIC:

Negative mode

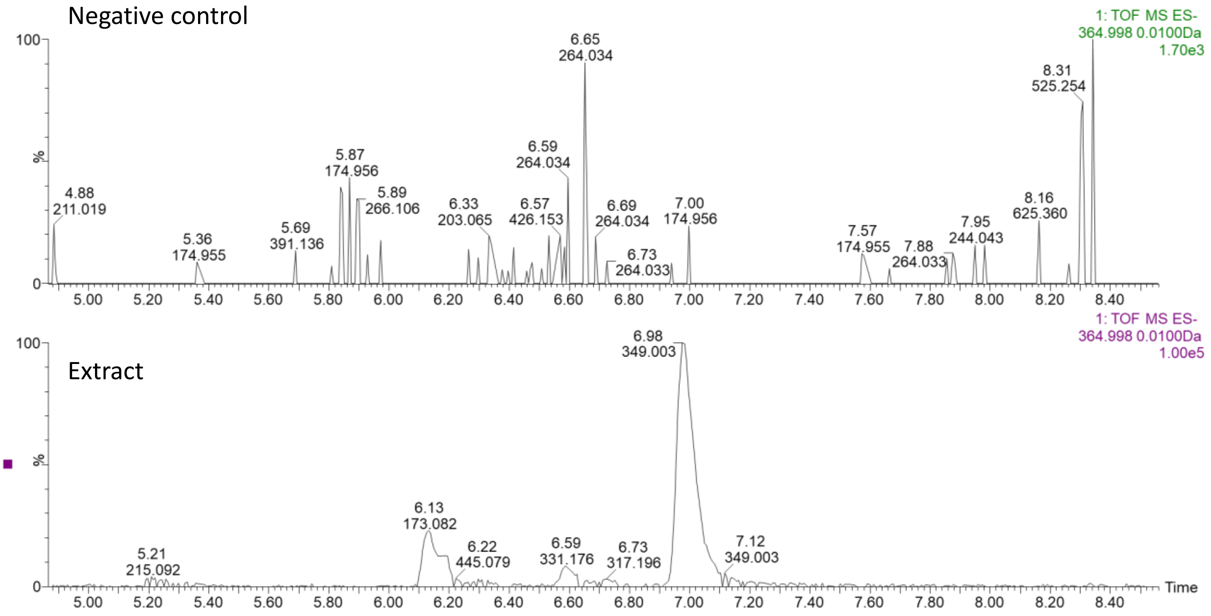

MS/MS spectrum:

Negative mode

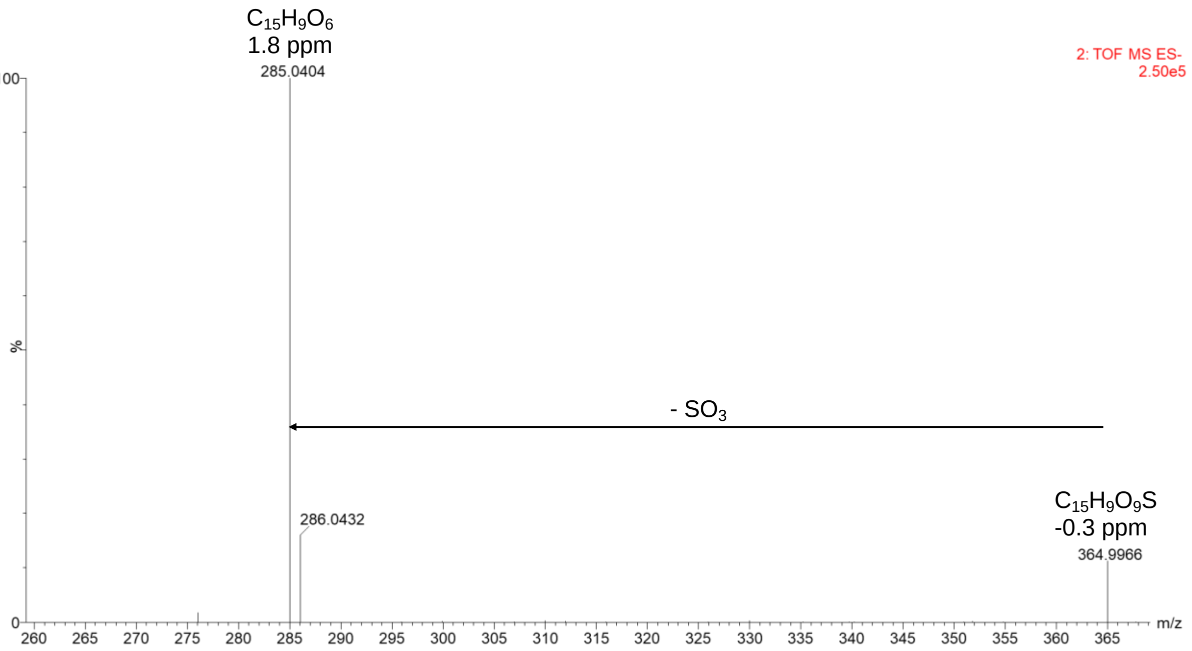

Compound ID: Gen_6

Compound name: Genistein+C7H16O

Chemical formula: $C_{22}H_{26}O_6Na$ (+)

m/z: 409.1632 (+)

Retention time: 9.51 min (+)

Confidence level: 4

XIC:

Positive mode

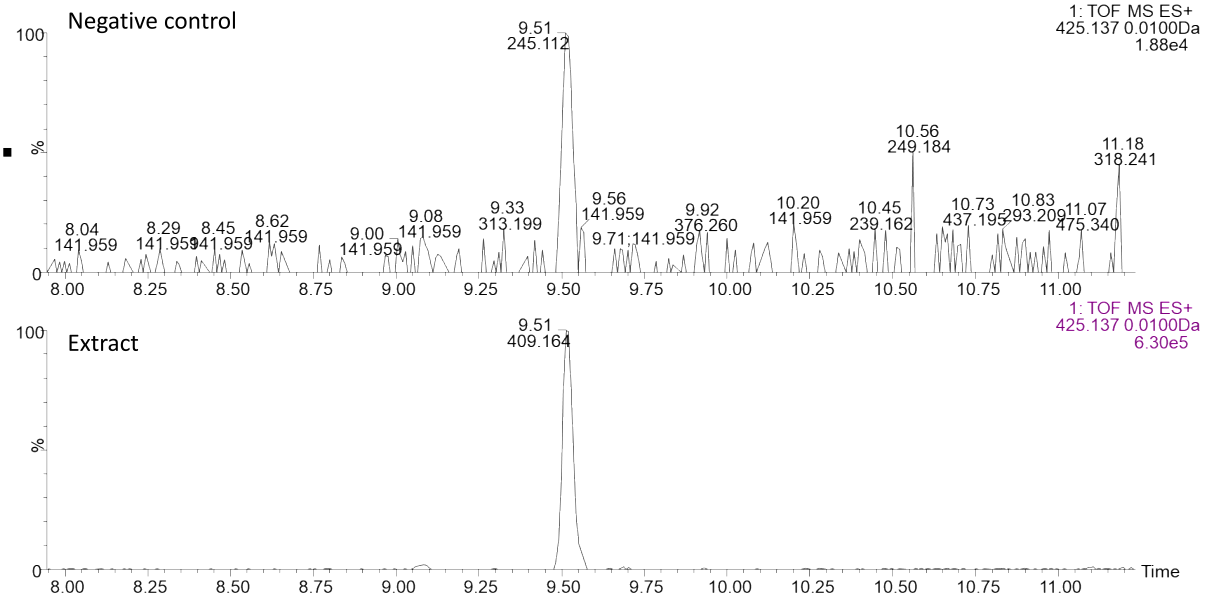

MS/MS spectrum:

Positive mode

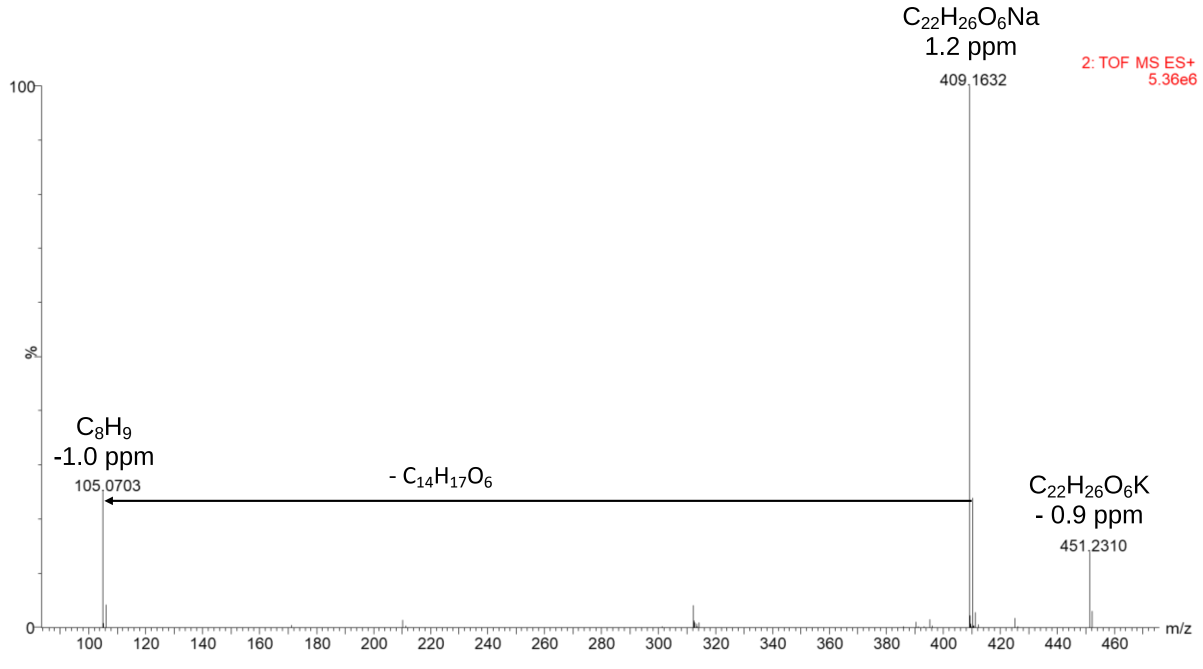

10.4 TPs of Ethoprophos

Compound ID: Et_0

Compound name: Ethoprophos (Parental compound)

Chemical formula: $C_8H_{20}O_2PS_2$ (+)

Proposed chemical structure:

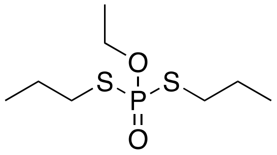

m/z: 243.0650 (+)

Retention time: 10.35 min (+)

Confidence level: 3

XIC (Positive mode):

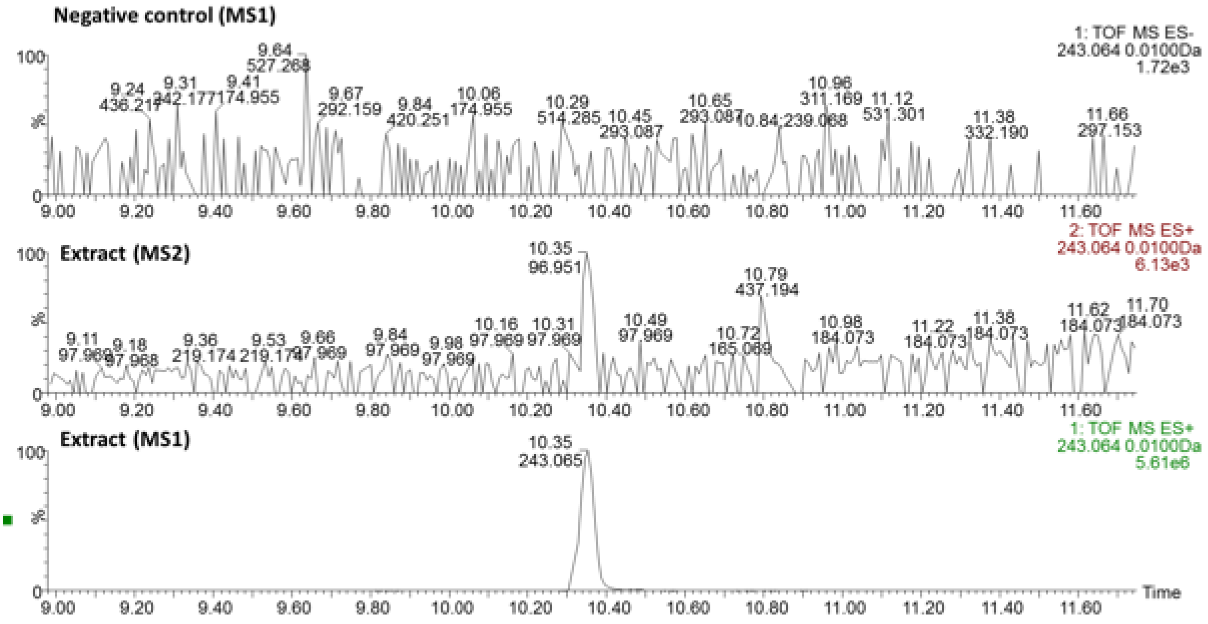

MS/MS Spectrum:

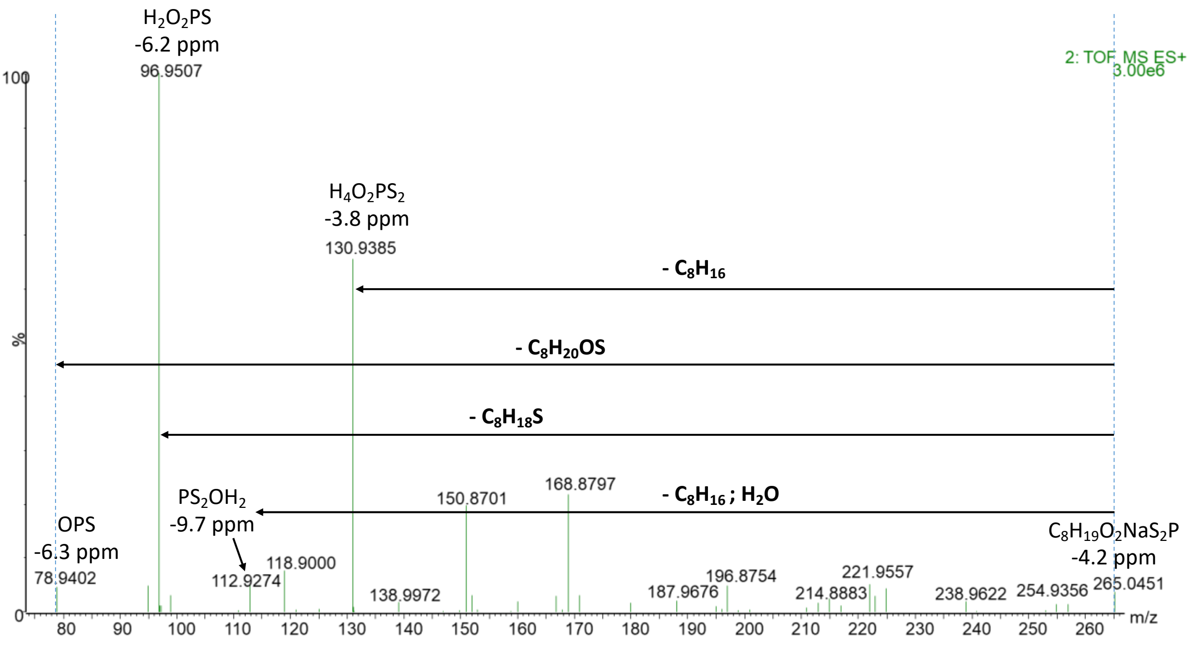

Compound ID: ET_1

Compound name: O-ethyl S-propyl phosphorothioate (EPPA)

Chemical formula: $C_5H_{12}O_3SP$ (-)

m/z: 183.025 (-)

Retention time: 5.67 min (-)

Confidence level: 3

Proposed Chemical structure:

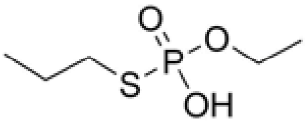

XIC (Negative mode):

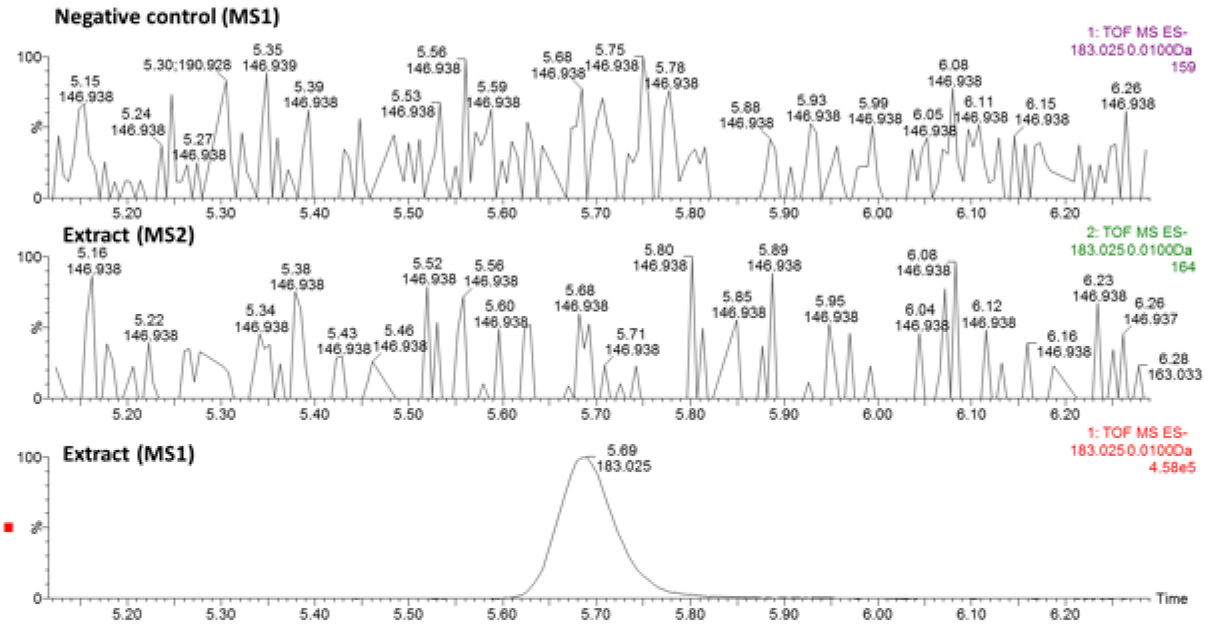

MS/MS-Spectrum (Negative mode):

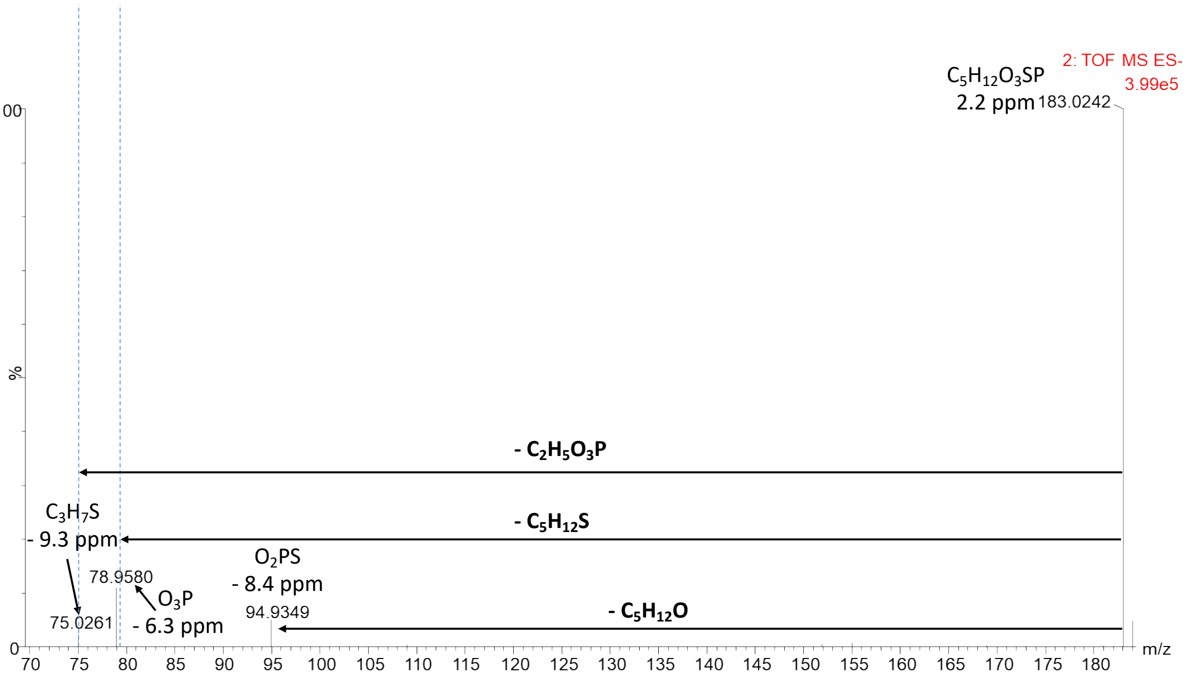

Compound ID: ET_2

Compound name: EPPA+O

Chemical formula: $C_5H_{12}O_4PS$ (-)

m/z: 199.019 (-)

Retention time: 3.03 min (-)

Confidence level: 4

Proposed Chemical structure:

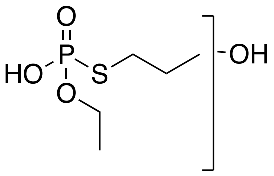

XIC:

Negative mode

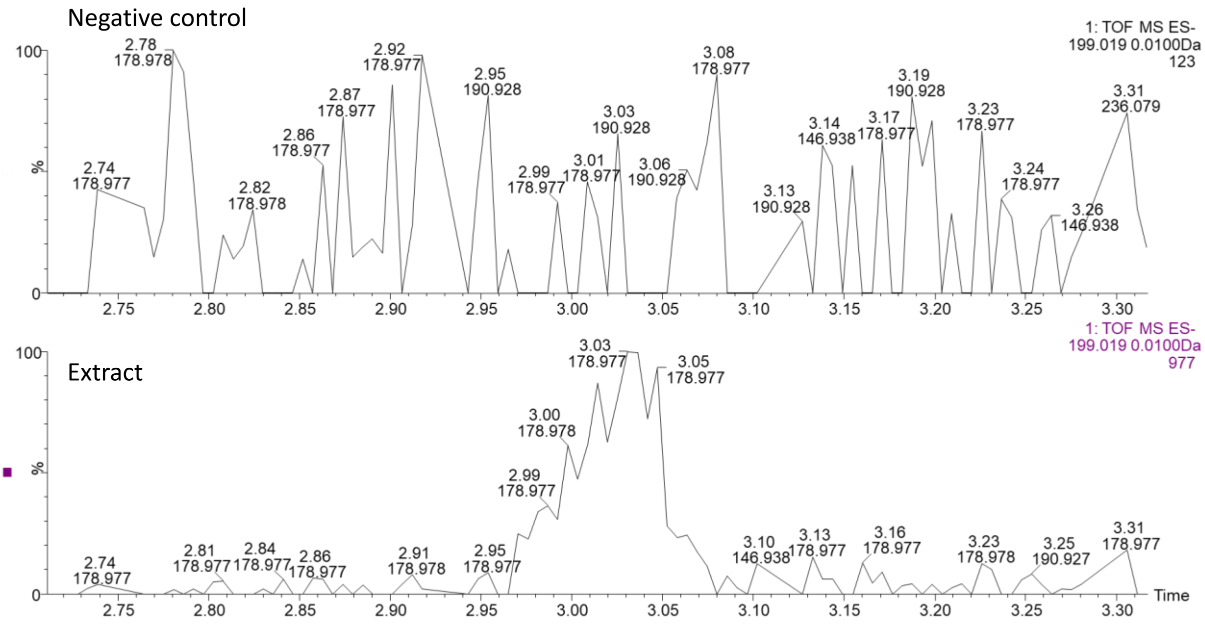

MS-spectrum:

Negative mode

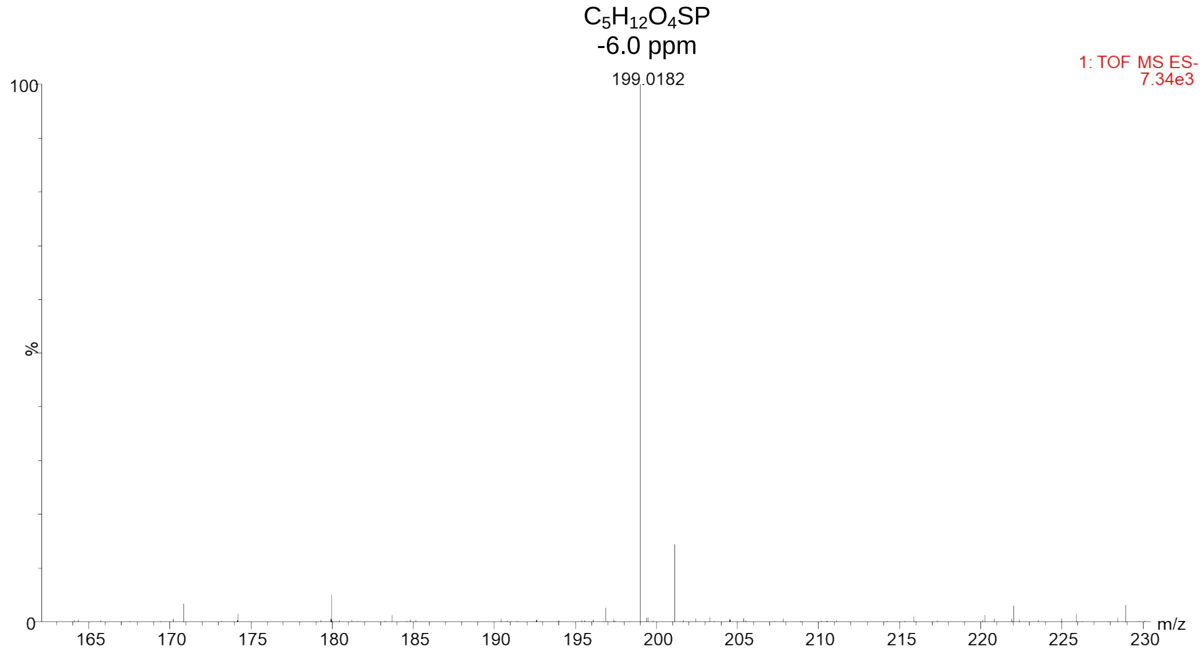

Compound ID: ET_3

Compound name: Ethylphosphate

Chemical formula: $C_2H_6O_4P$ (-)

m/z: 124.9997 (-)

Retention time: 4.33 min (-)

Confidence level: 3

Proposed Chemical structure:

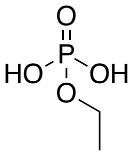

XIC:

Negative mode

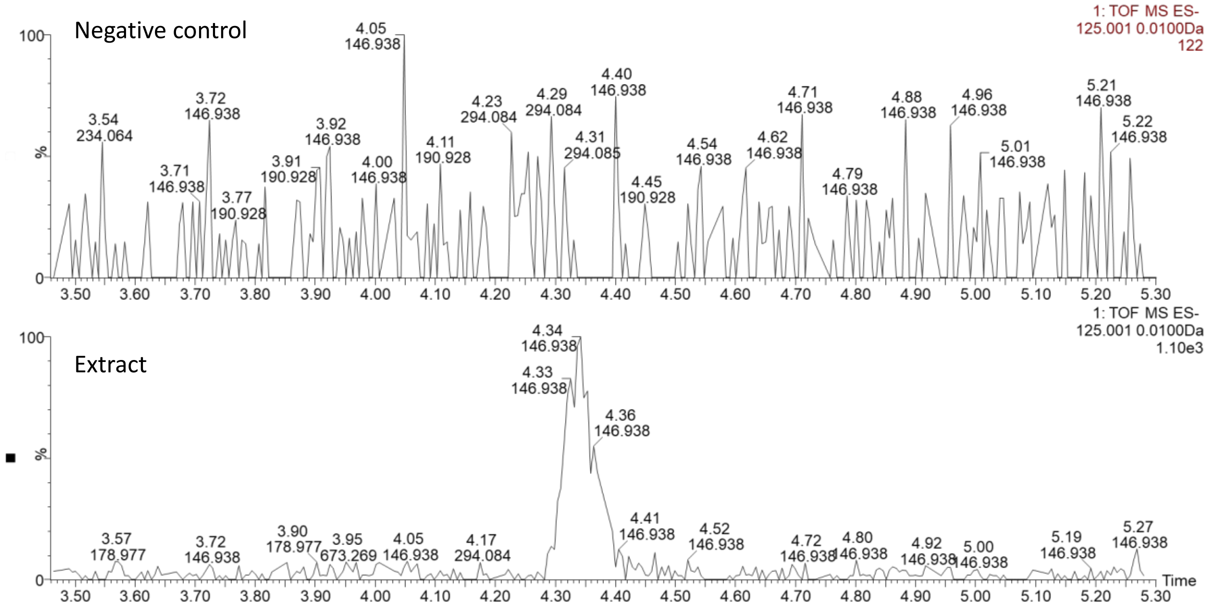

MS-spectrum:

Negative mode

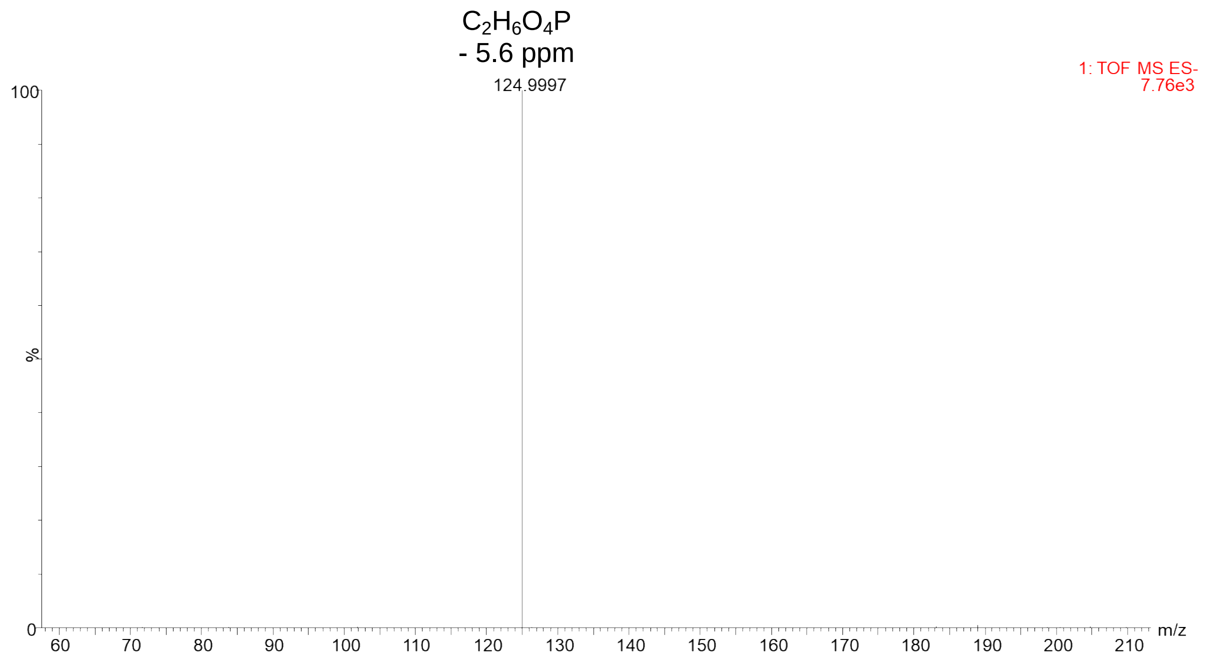

Compound ID: ET_4

Compound name: O-ethyl-O-methyl-S-propyl-phosphorothioate

Chemical formula: $C_6H_{14}O_3PS$ (-)

m/z: 197.0391 (-)

Retention time: 7.02 + 7.33 min (-)

Confidence level: 3

Proposed Chemical structure:

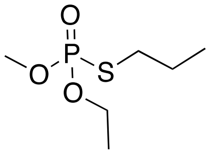

XIC:

Negative mode

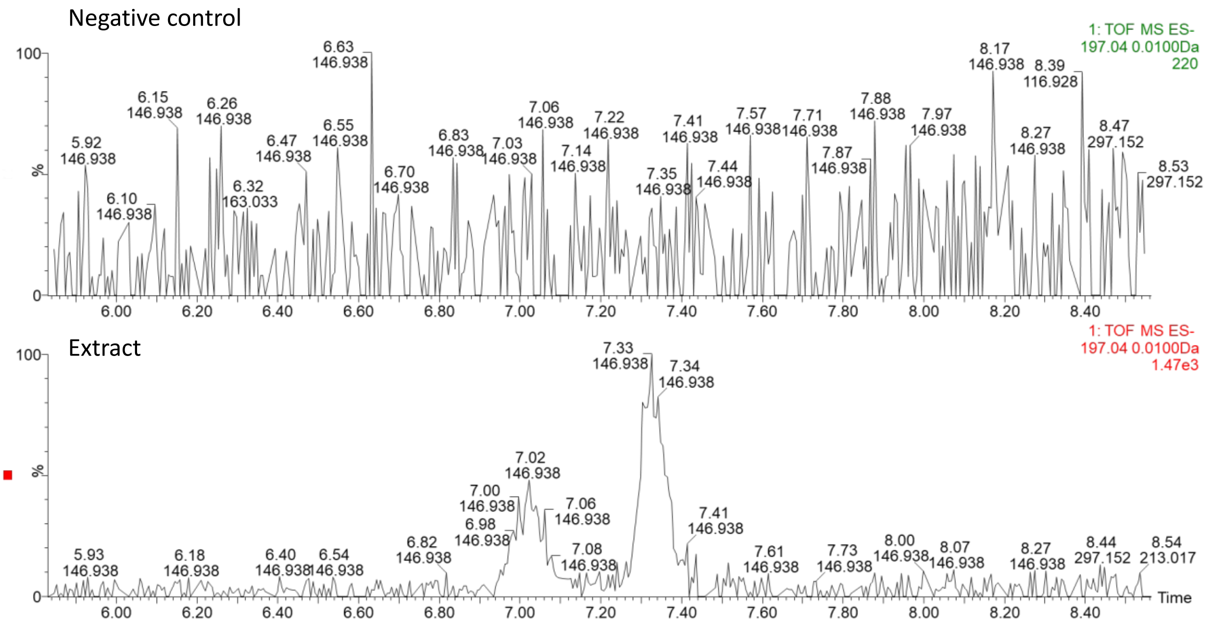

MS-spectrum:

Negative mode

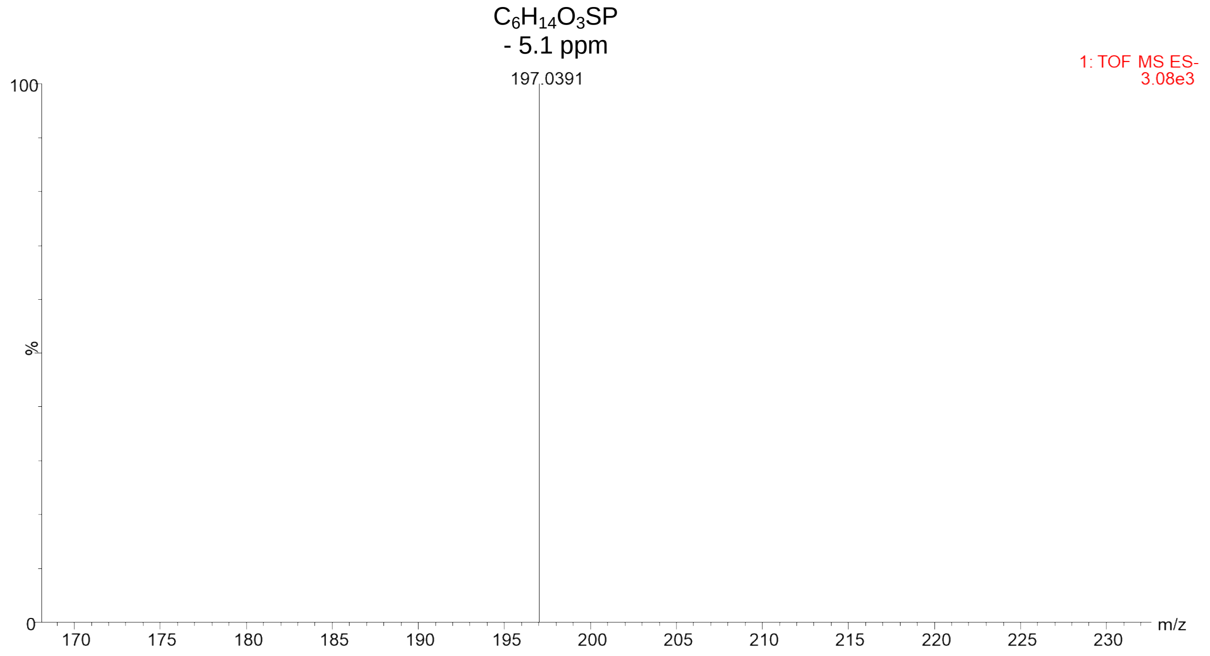

Compound ID: ET_5

Compound name: O-ethyl S-methyl S-propyl phosphorodithioate

Chemical formula: $C_6H_{14}O_2PS_2$ (-)

m/z: 213.0171 (-)

Retention time: 8.54 min (-)

Confidence level: 3

Proposed Chemical structure:

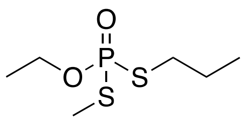

XIC:

Negative mode

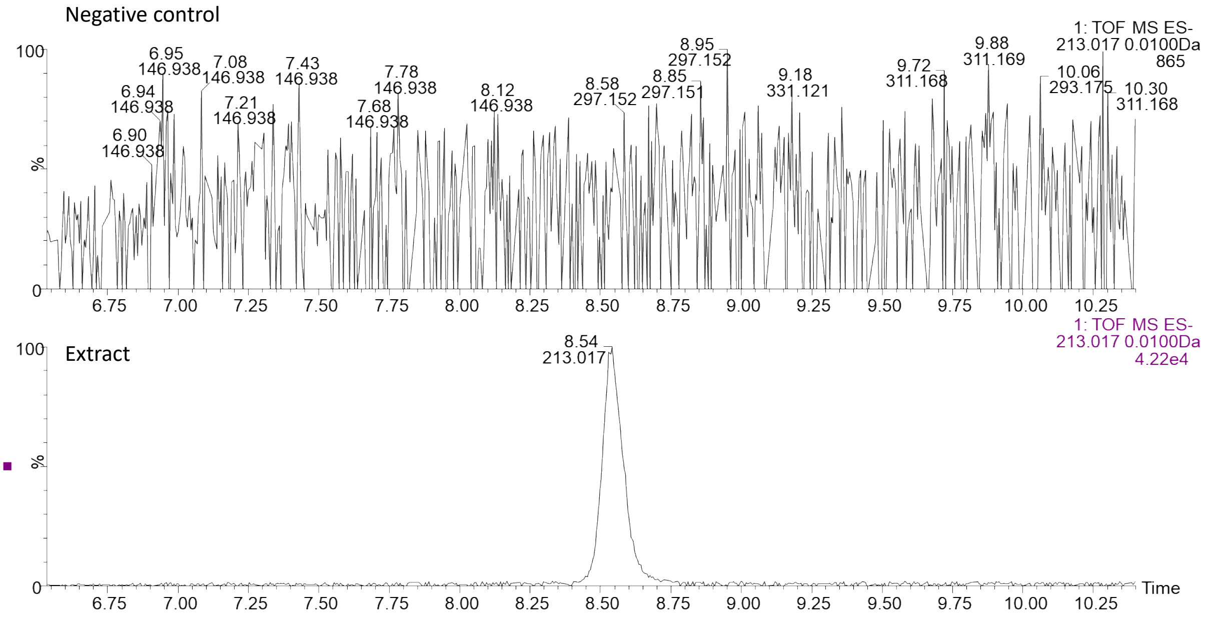

MS-Spectrum

Negative mode:

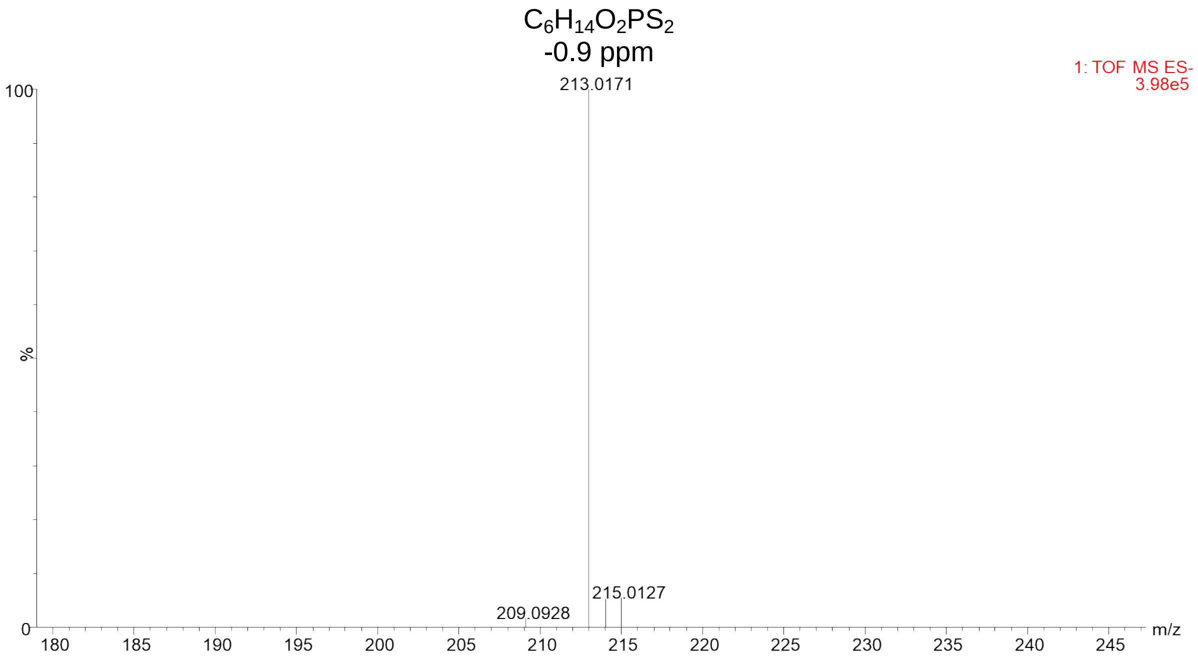

10.5 TPs of Dexamethasone

Compound ID: Dex_0

Compound name: Dexamethasone (Parental compound)

Chemical formula: $C_{22}H_{29}O_5FNa$ (+)

m/z: 415.1899 (sodium adduct) (+)

Retention time: 9.07 min (+)

Confidence level: 3

Proposed chemical structure:

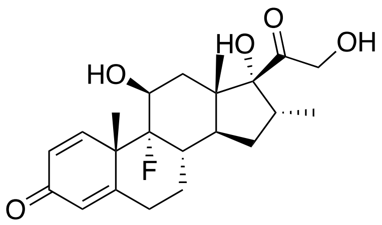

XIC:

Positive mode:

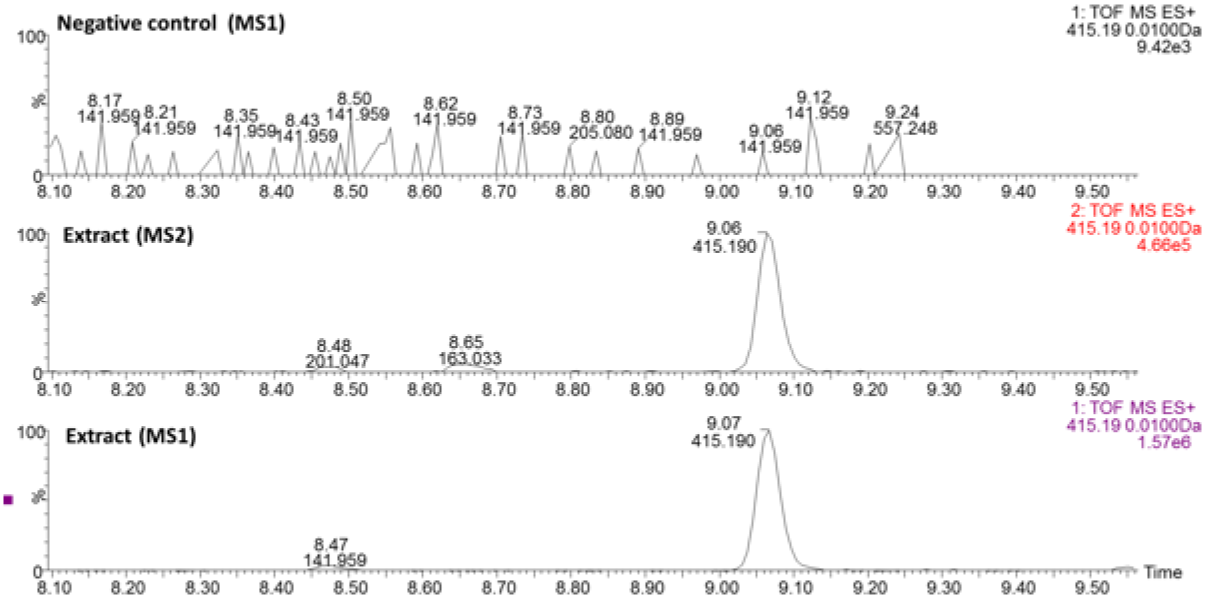

MS/MS spectrum:

Positive mode:

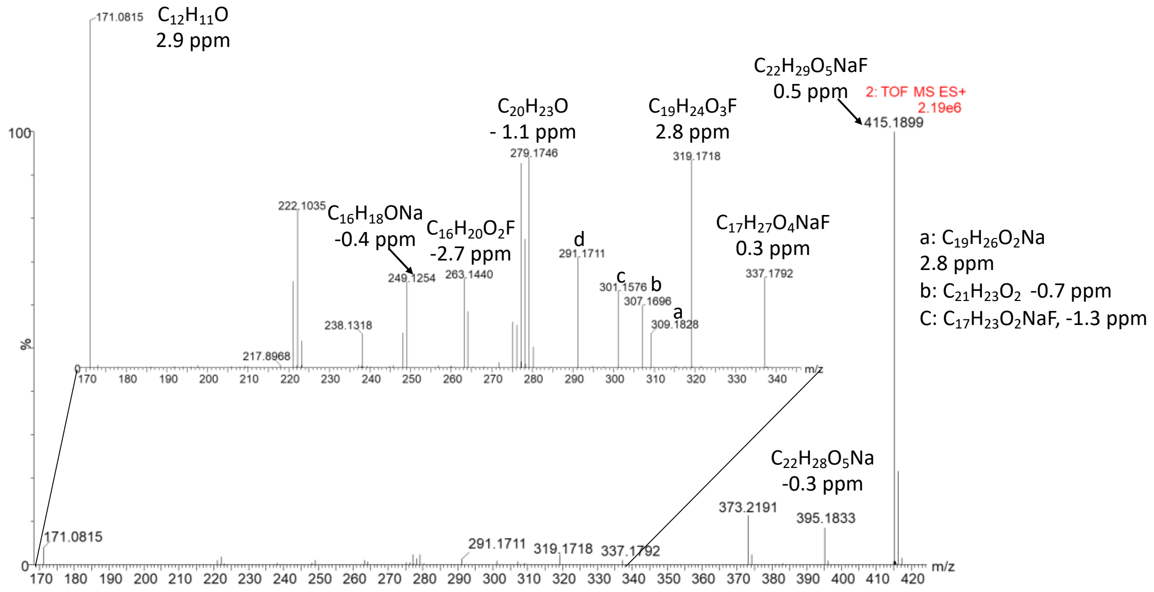

Compound ID: Dex_1

Compound name: Dexamethasone+Glucuronide-2H

Chemical formula: $C_{28}H_{36}O_{11}F$ (+)

m/z: 567.224 (+)

Retention time: 7.80 min (+)

Confidence level: 3

Proposed chemical structure:

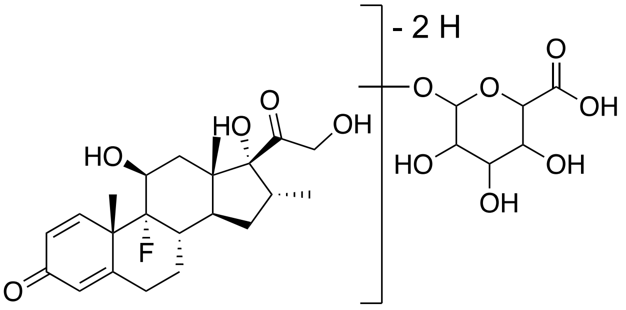

XIC:

Positive mode

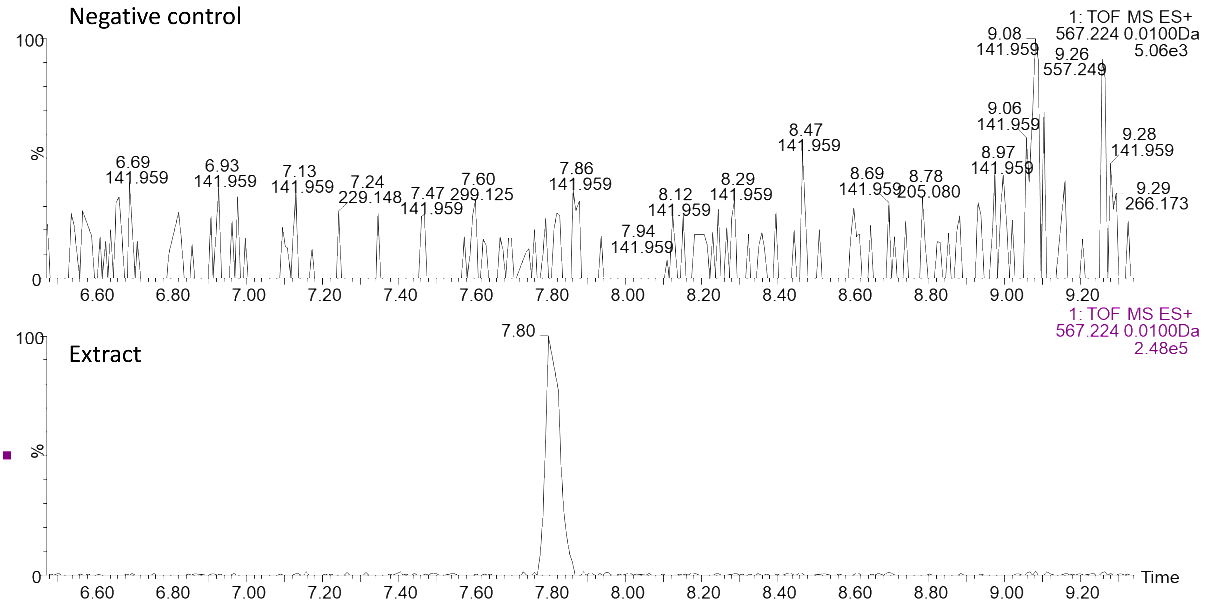

MS-Spectrum:

Positive mode

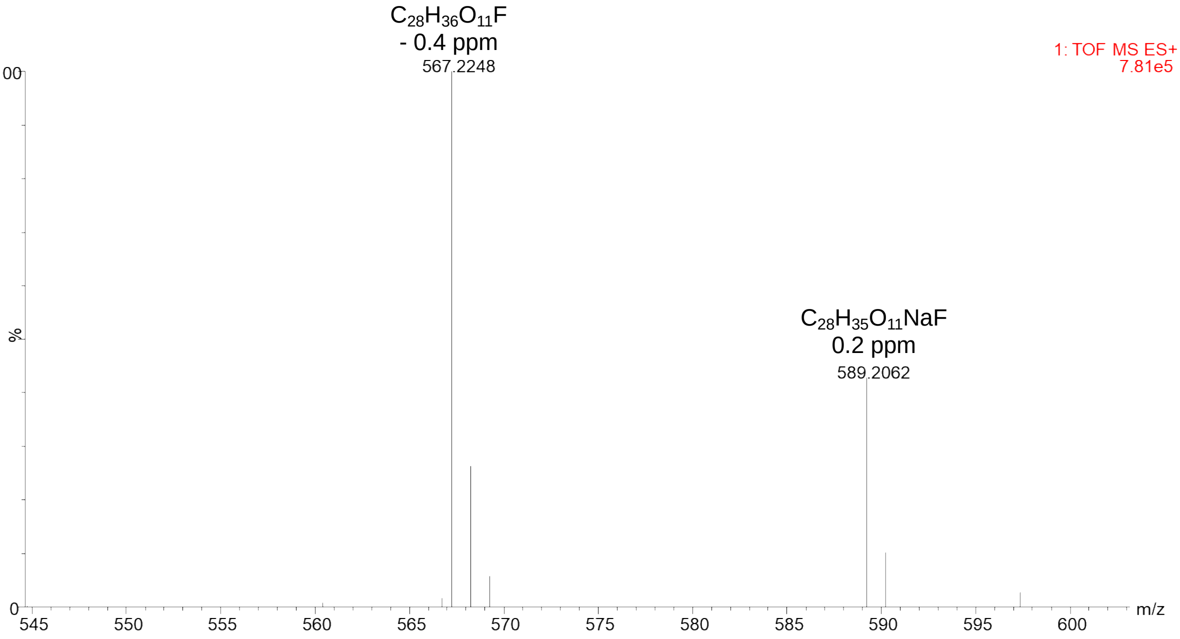

Compound ID: Dex_2

Compound name: Dexamethasone+Glucuronide

Chemical formula: $C_{28}H_{38}O_{11}F$ (+) / $C_{28}H_{36}O_{11}F$ (-)

m/z: 569.2395 (+) / 567.2213 (-)

Retention time: 8.66 min (+)

Confidence level: 3

Proposed chemical structure:

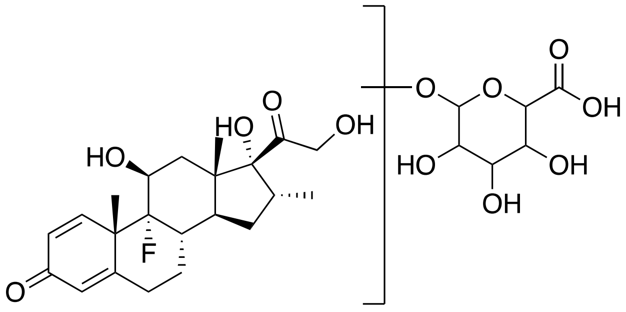

XIC:

Positive mode:

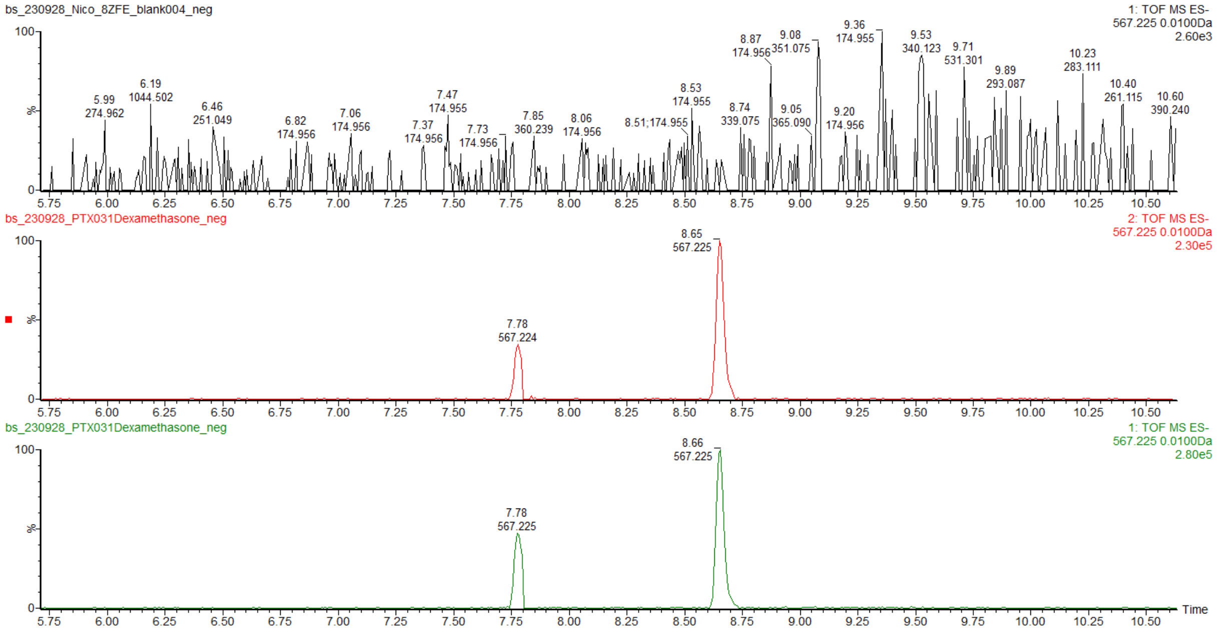

MS-spectrum:

Positive mode:

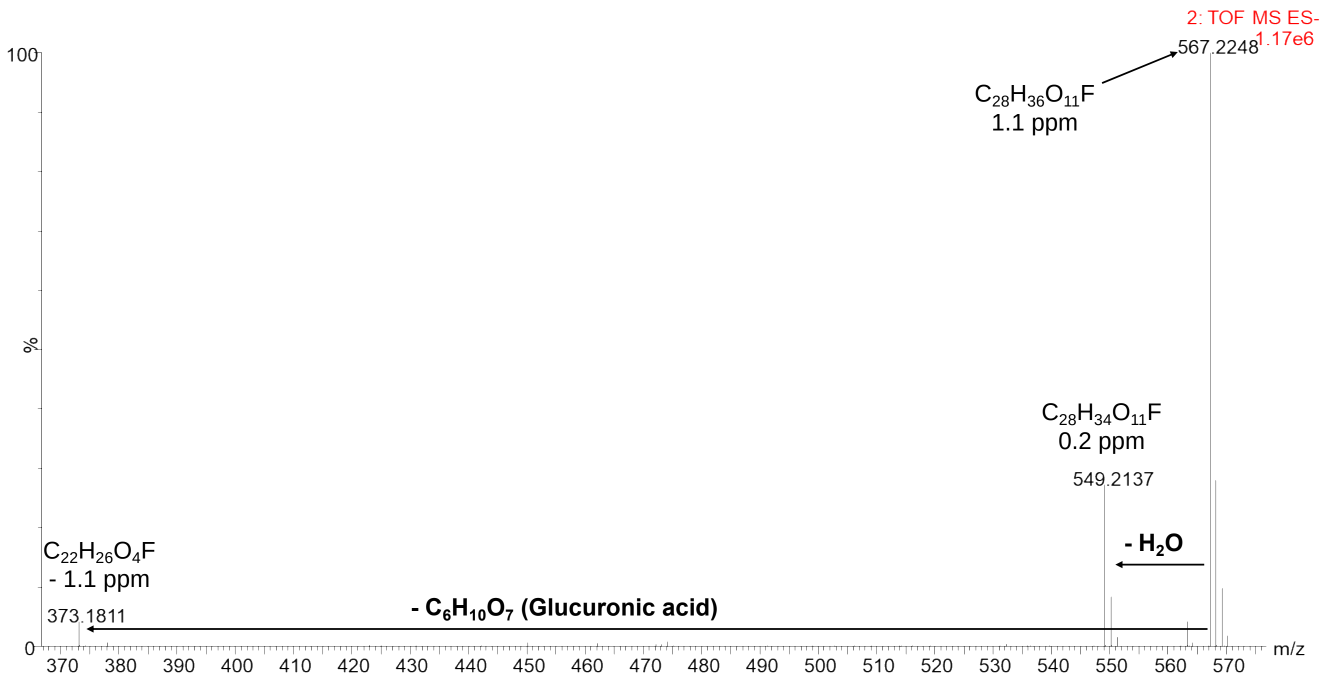

Compound ID: Dex_3

Compound name: Dexamethasone-2H

Chemical formula: C22H27O5NaF / C22H28O5F (+)

m/z: 413.19 / 391.1932 (+)

Retention time: 8.47 min (+)

Confidence level: 4

Proposed chemical structure:

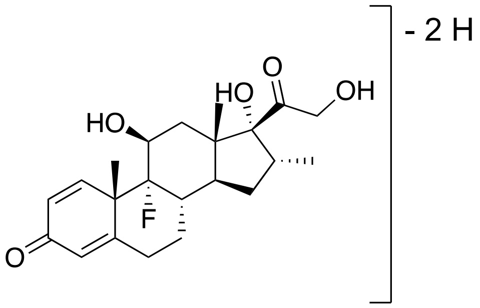

XIC:

Positive mode

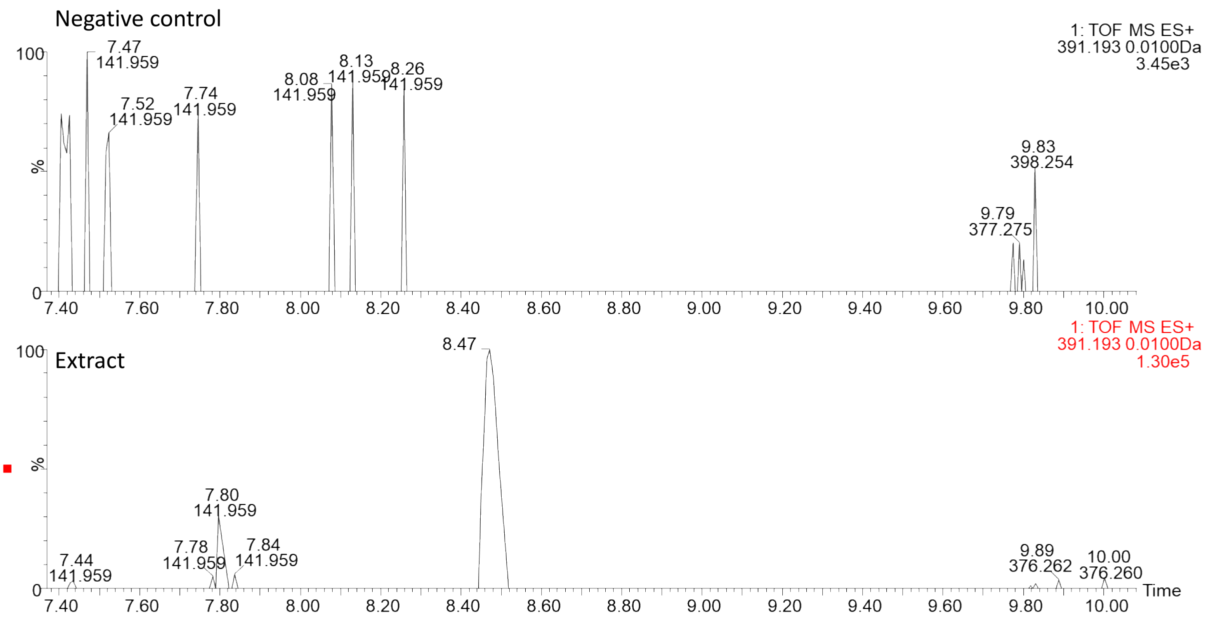

MS-spectrum:

Positive mode

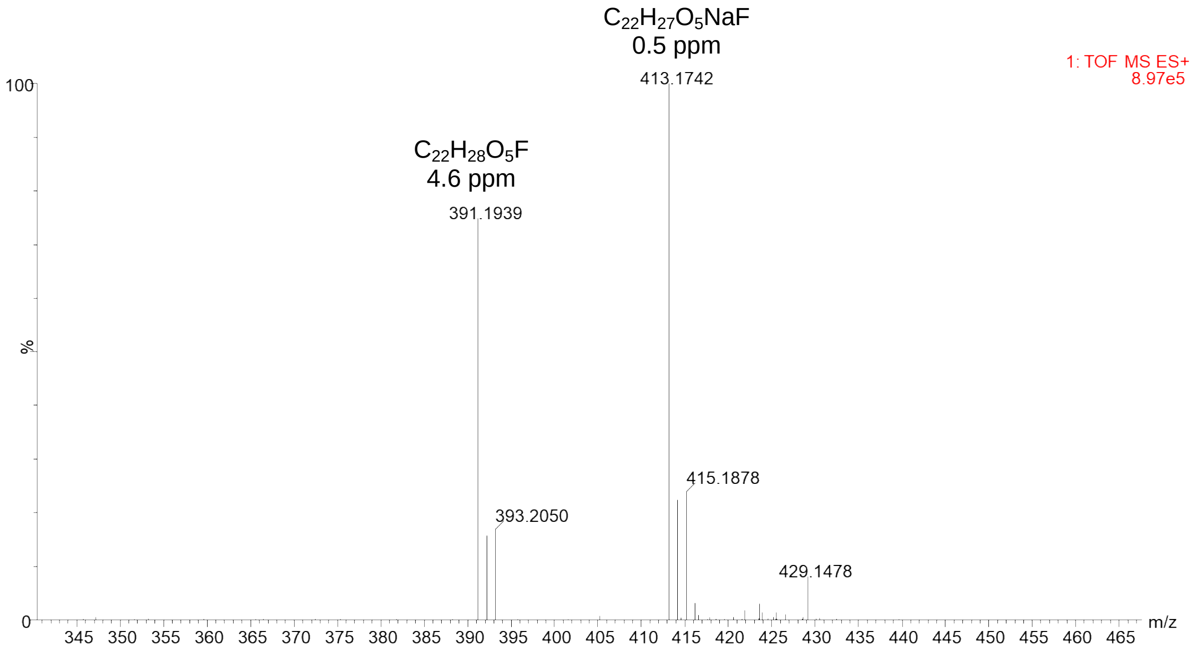

Compound ID: Dex_4

Compound name: Dexamethasone+SO3

Chemical formula: C22H28O8SF (-)

m/z: 471.149 (-)

Retention time: 7.53+8.52min (+)

Confidence level: 3

Proposed chemical structure:

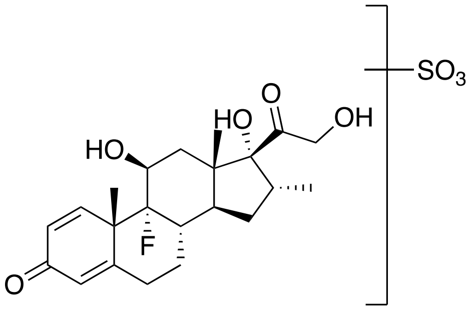

XIC:

Negative mode

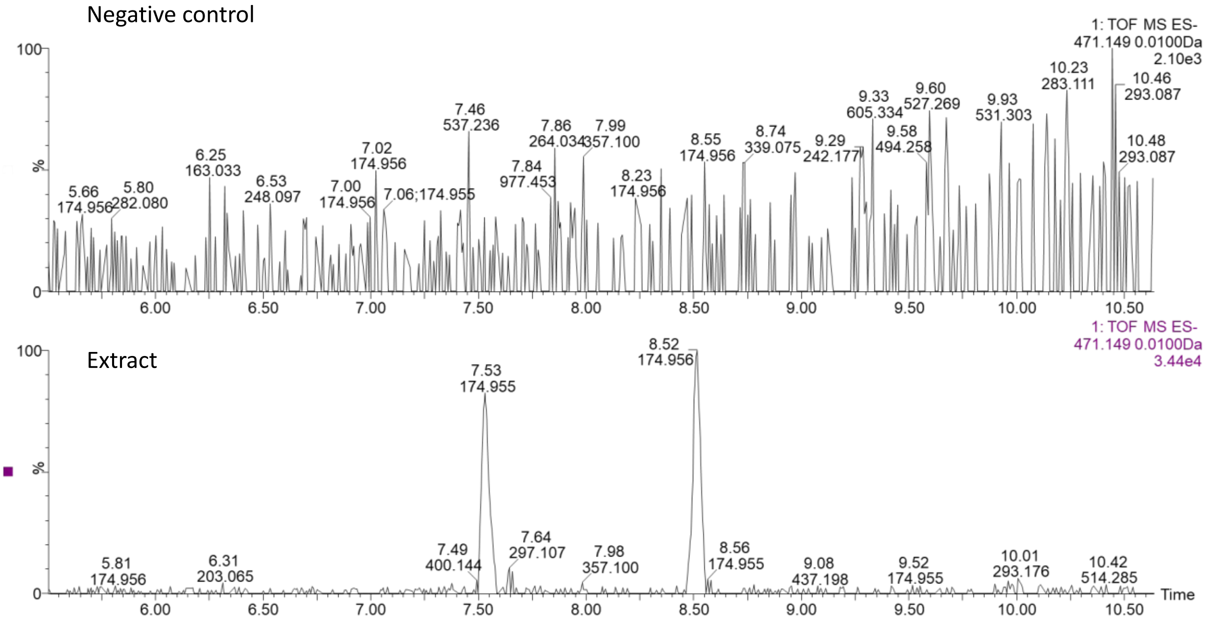

MS-spectrum:

Negative Mode

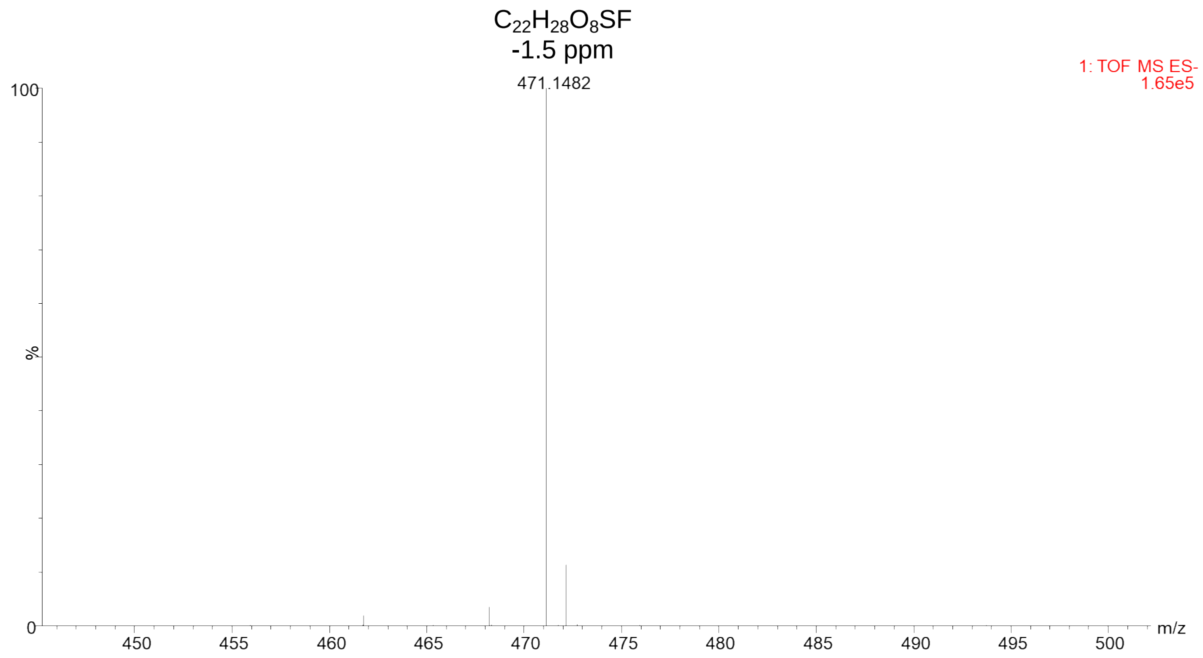

10.6 TPs of Niflumic acid

Compound ID: NA_0

Compound name: Niflumic acid (Parental compound)

Chemical formula: $C_{13}H_{10}N_2O_2F_3$ (+) / $C_{13}H_8N_2O_2F_3$ (-)

m/z: 283.0698 (+) / 281.055

Retention time: 10.48 min (+) / 10.49 min (-)

Confidence level: 3

Proposed chemical structure:

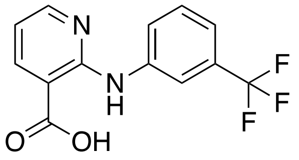

XIC:

Positive mode:

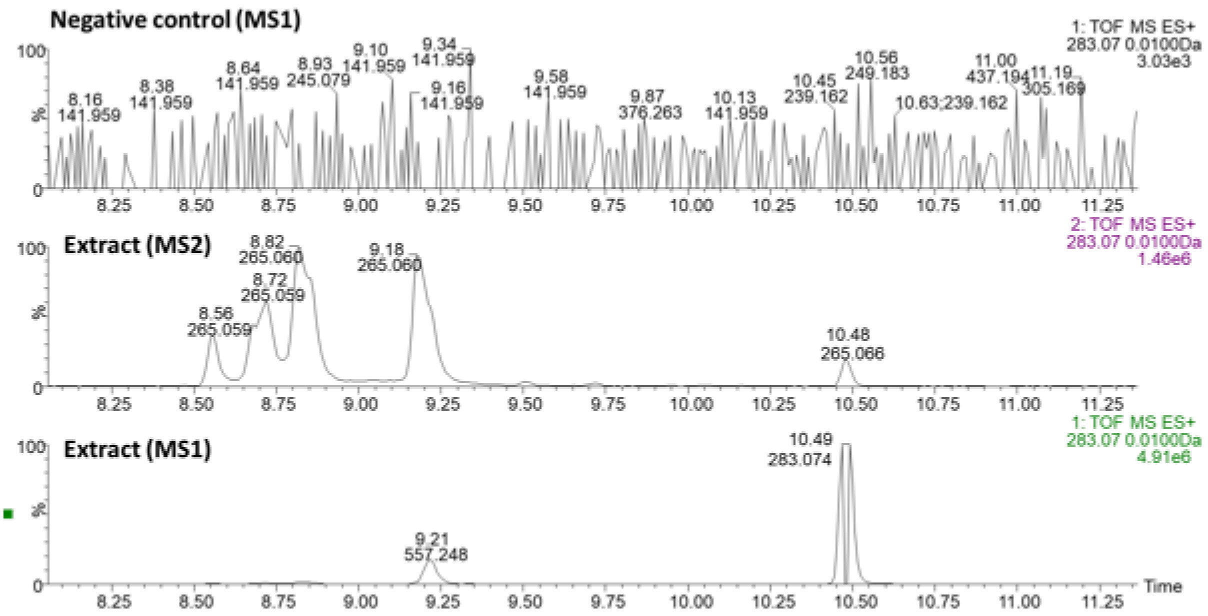

MS/MS spectrum:

Positive mode:

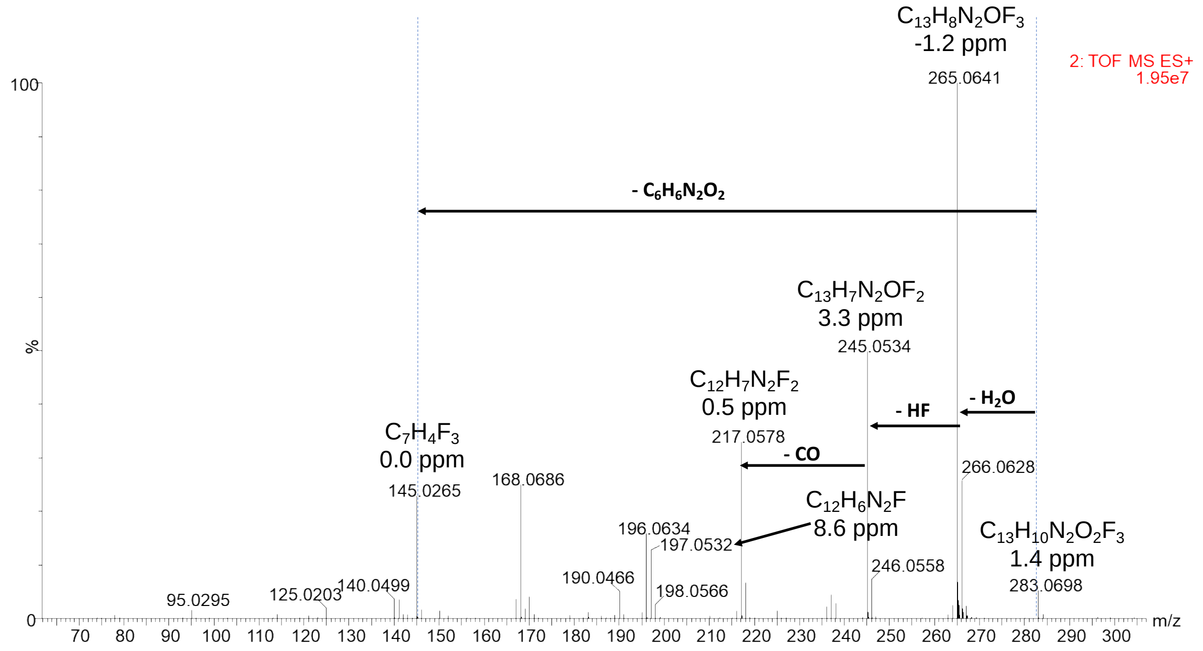

Compound ID: NA_1

Compound name: Niflumic acid + Glucuronide (isomers; N- or O-glucuronidation)

Chemical formula: $C_{19}H_{18}N_2O_8F_3$ (+) / $C_{19}H_{16}N_2O_8F_3$ (-)

m/z: 459.1015 (+) / 457.0855 (-)

Retention time: 5.87 **+** 8.55 + 8.68 +8.85 + 9.19 min

Confidence level: 3

Proposed chemical structure:

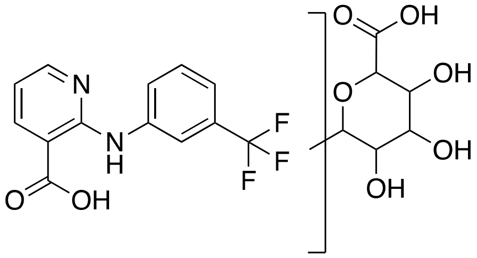

XIC:

Positive mode:

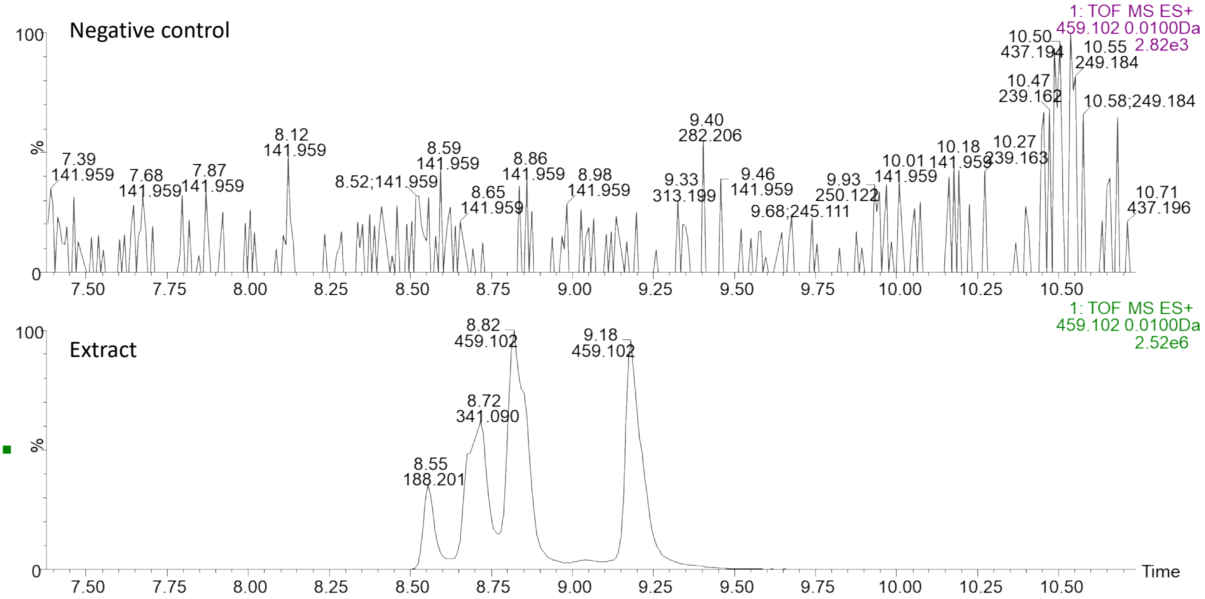

Negative mode:

MS/MS spectrum:

Positive mode:

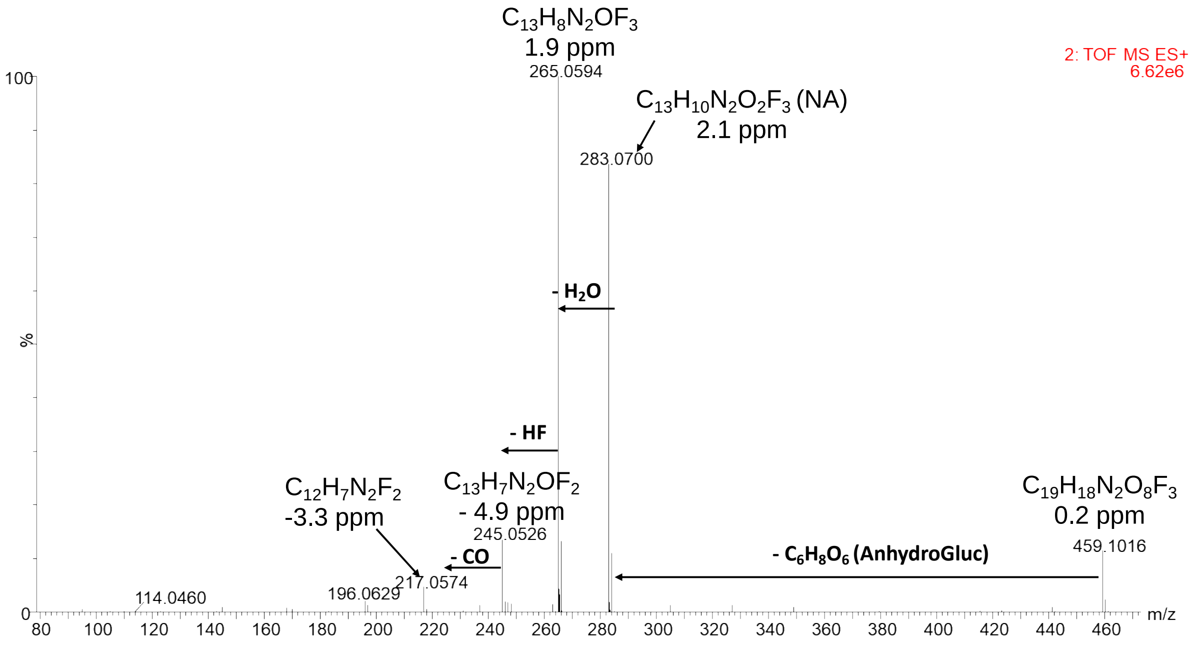

Compound ID: NA_2

Compound name: Niflumic acid+O+Glucuronide

Chemical formula: $C_{19}H_{18}N_2O_9F_3$ (+) / $C_{19}H_{16}N_2O_9F_3$ (-)

m/z: 475.0963 (+) / 473.09100 (-)

Retention time: 5.7 min (+) / 5.7 min

Confidence level: 3

Proposed chemical structure:

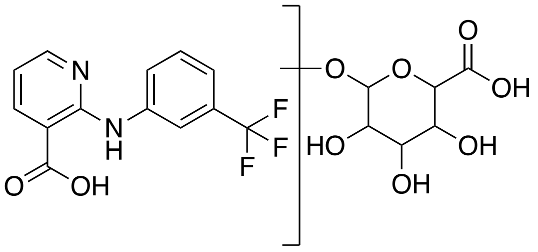

XIC:

Positive mode:

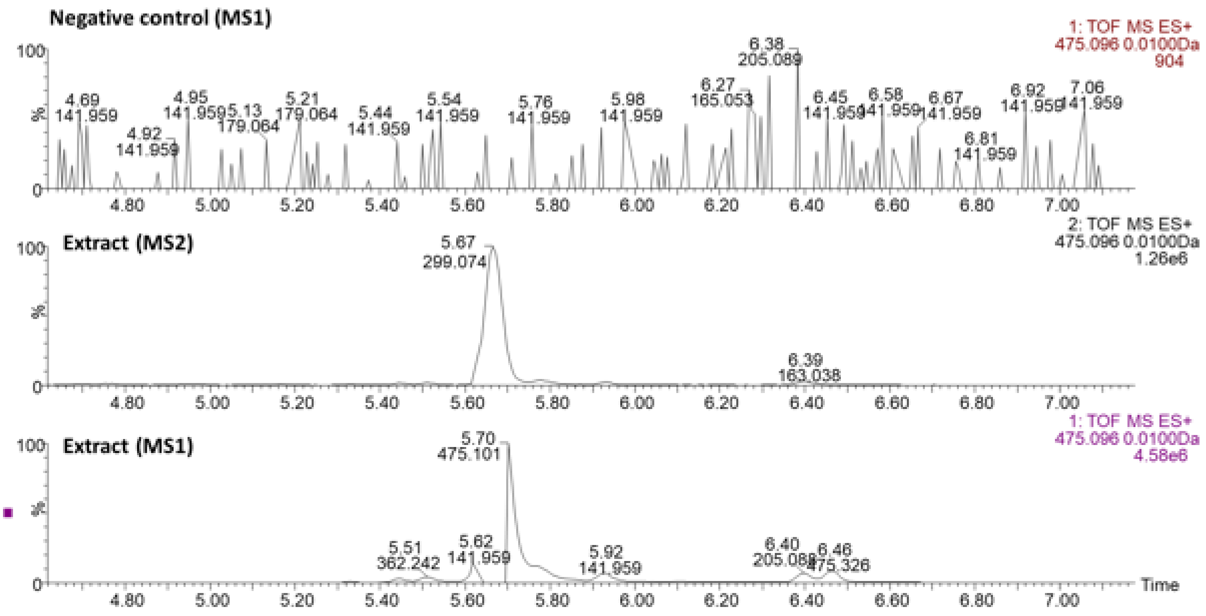

MS/MS spectrum:

Positive mode:

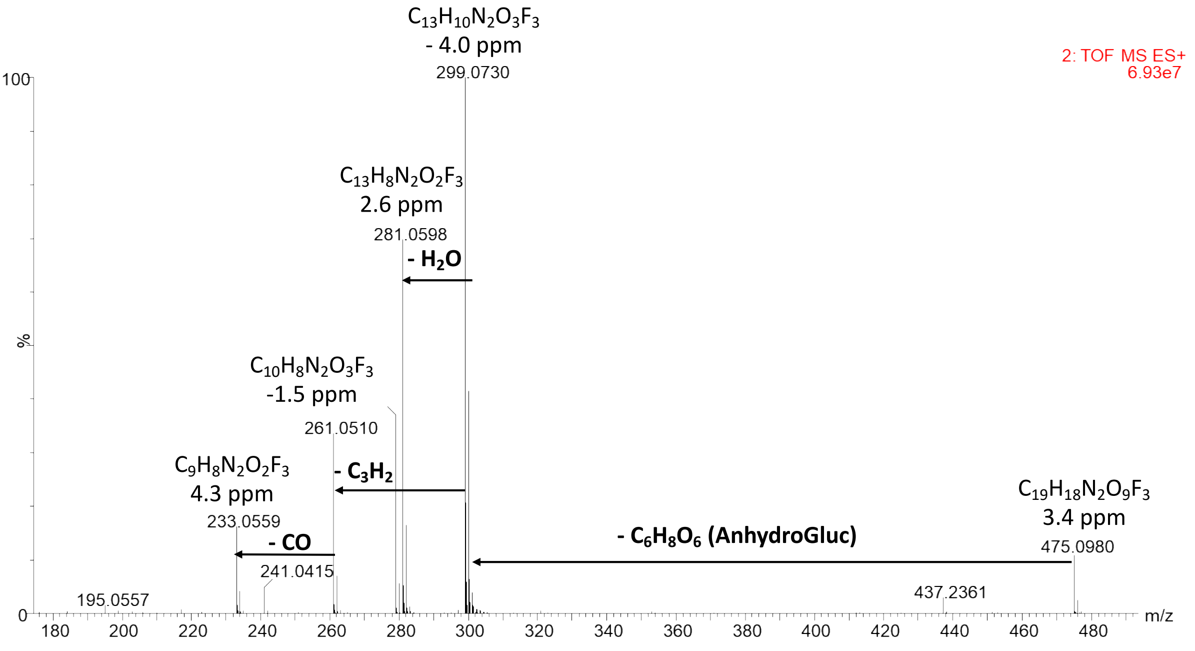

Compound ID: NA_3

Compound name: Niflumic acid+O

Chemical formula: $C_{13}H_{10}N_2O_3F_3$ (+) / $C_{13}H_8N_2O_3F_3$ (-)

m/z: 299.0753 (+) / 297.0493 (-)

Retention time: 6.83 min (+) / 6.83 min (-)

Confidence level: 3

Proposed chemical structure:

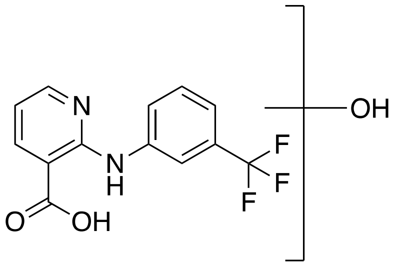

XIC:

Negative mode:

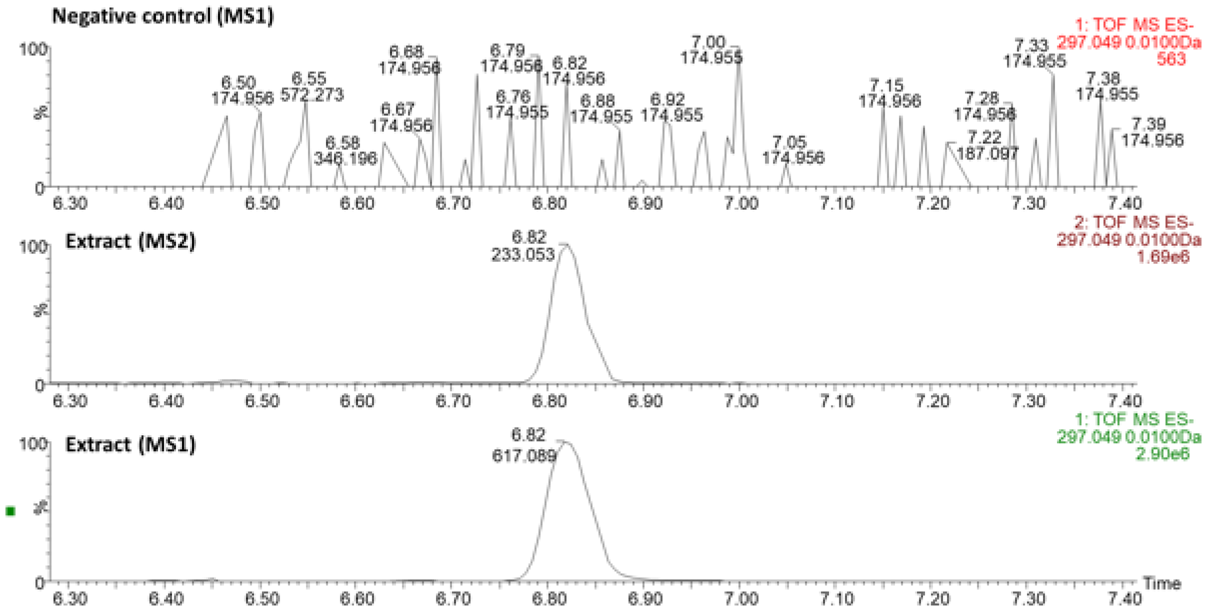

MS/MS spectrum:

Negative mode:

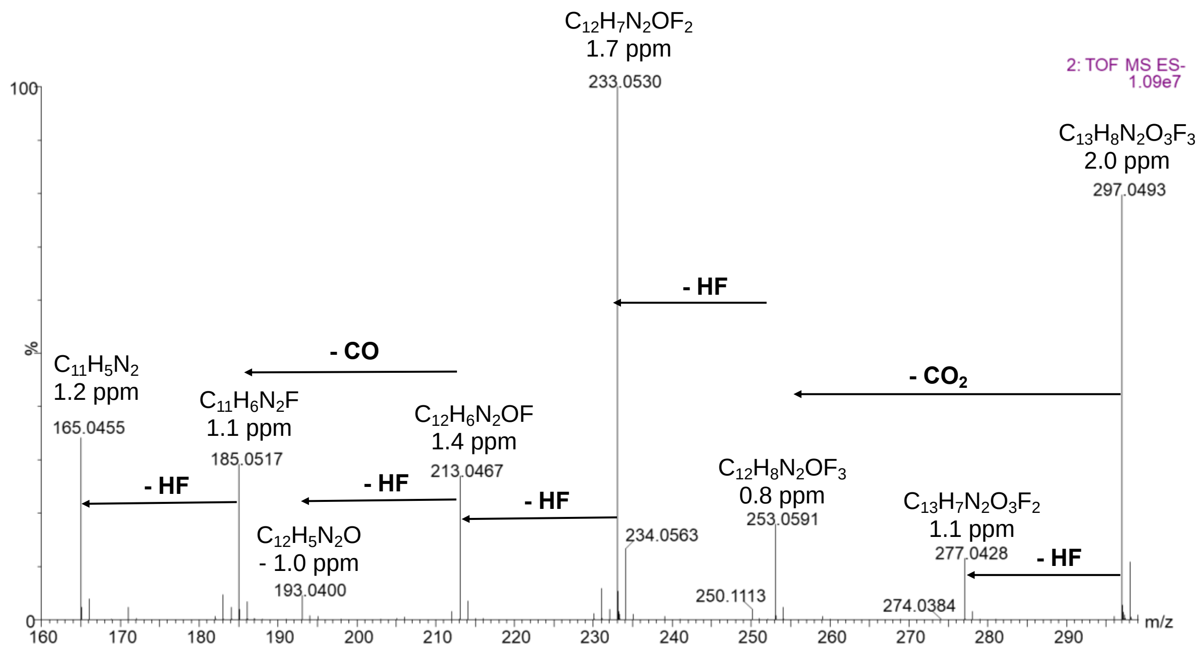

Compound ID: NA_4

Compound name: Niflumic acid+Gly

Chemical formula: $C_{15}H_{13}N_3O_3F_3$ (+)

m/z: 340.0907 (+)

Retention time: 9.65 min (+)

Confidence level: 3

Proposed chemical structure:

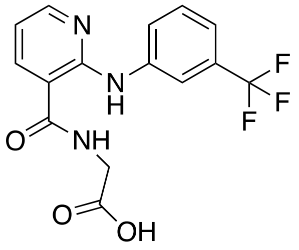

XIC:

Positive mode:

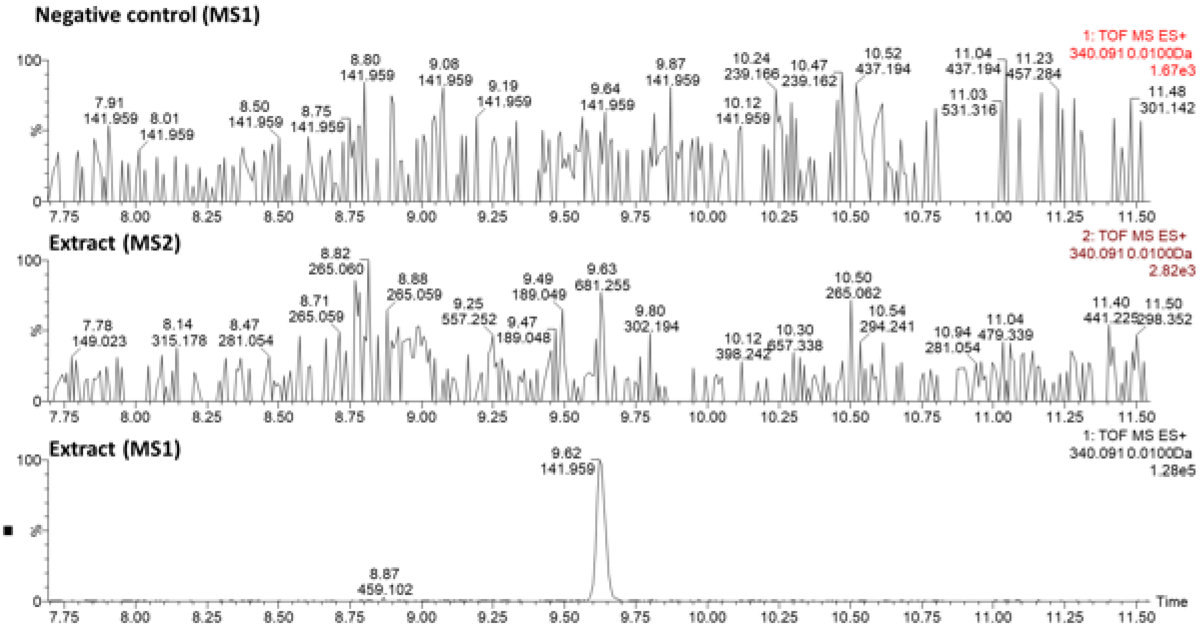

MS-spectrum:

Positive mode:

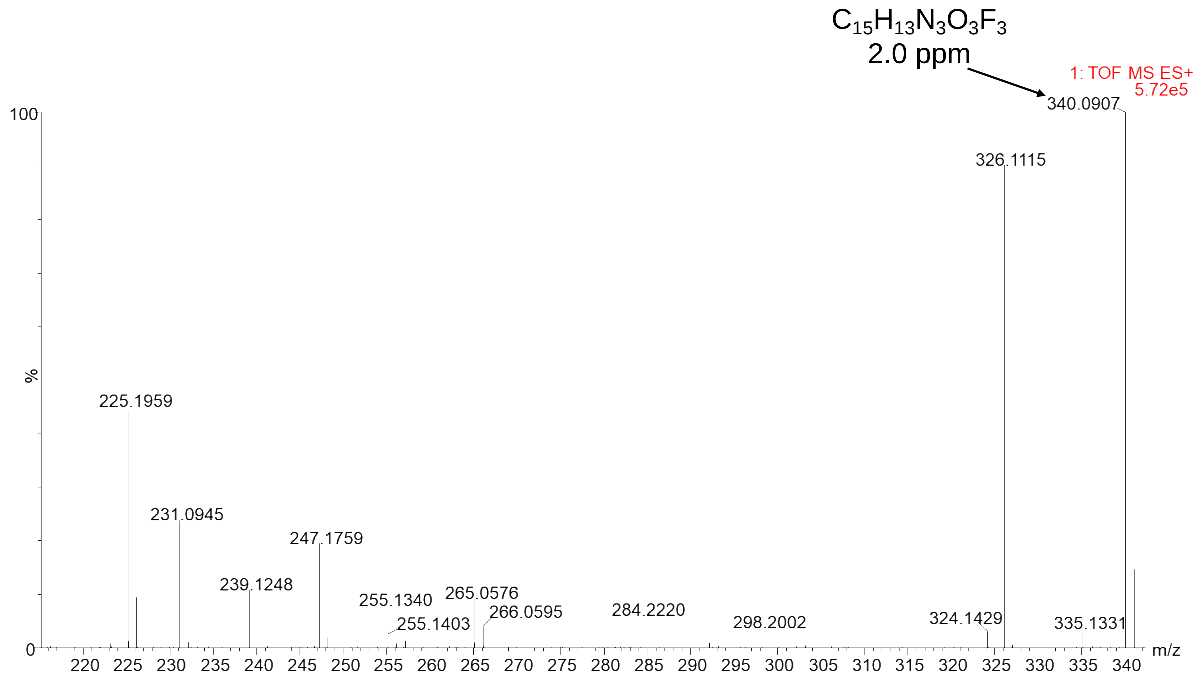

Compound ID: NA_5

Compound name: Niflumic acid+O+SO3

Chemical formula: $C_{13}H_{10}N_2O_6F_3S$ (+)

m/z: 379.0212 (+) / 377.0067 (-)

Retention time: 8.28 min

Confidence level: 3

Proposed chemical structure:

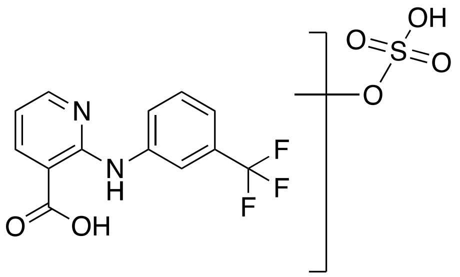

XIC:

Negative mode:

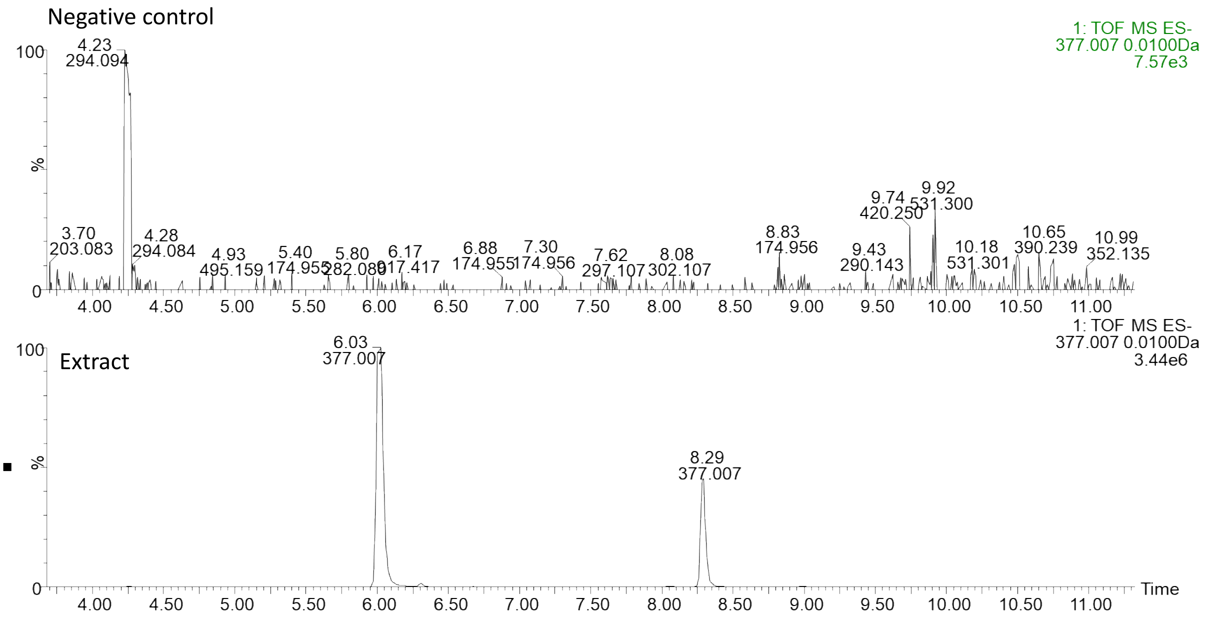

MS/MS spectrum:

Negative mode:

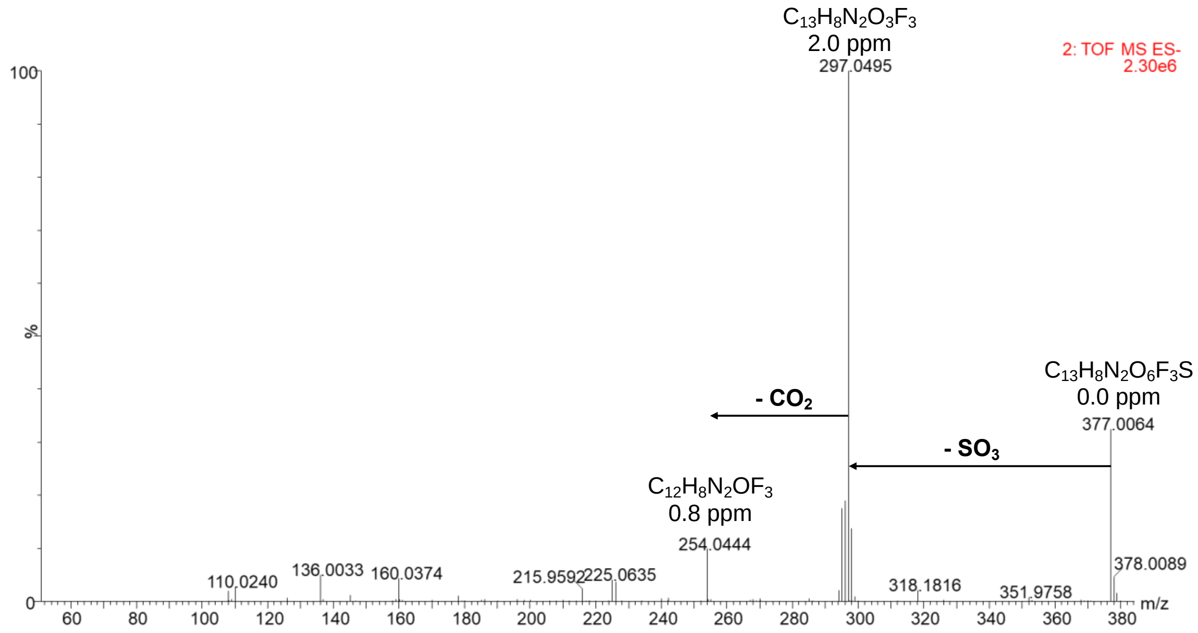

Compound ID: NA_6

Compound name: Niflumic acid + SO4

Chemical formula: $C_{13}H_8N_2O_6F_3S$ (-) ($C_{13}H_7N_2O_6F_3SNa$)

m/z: 377.0054 (-) (398.9880)

Retention time: 6.01 min (-)

Confidence level: 3

XIC:

Negative mode

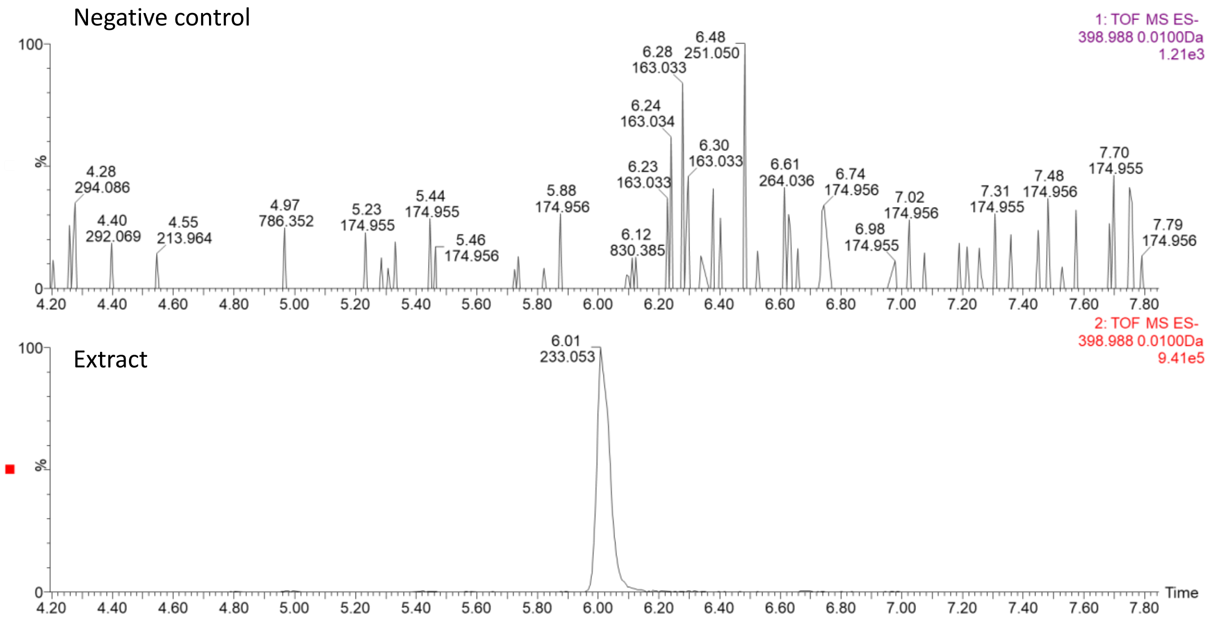

Negative mode:

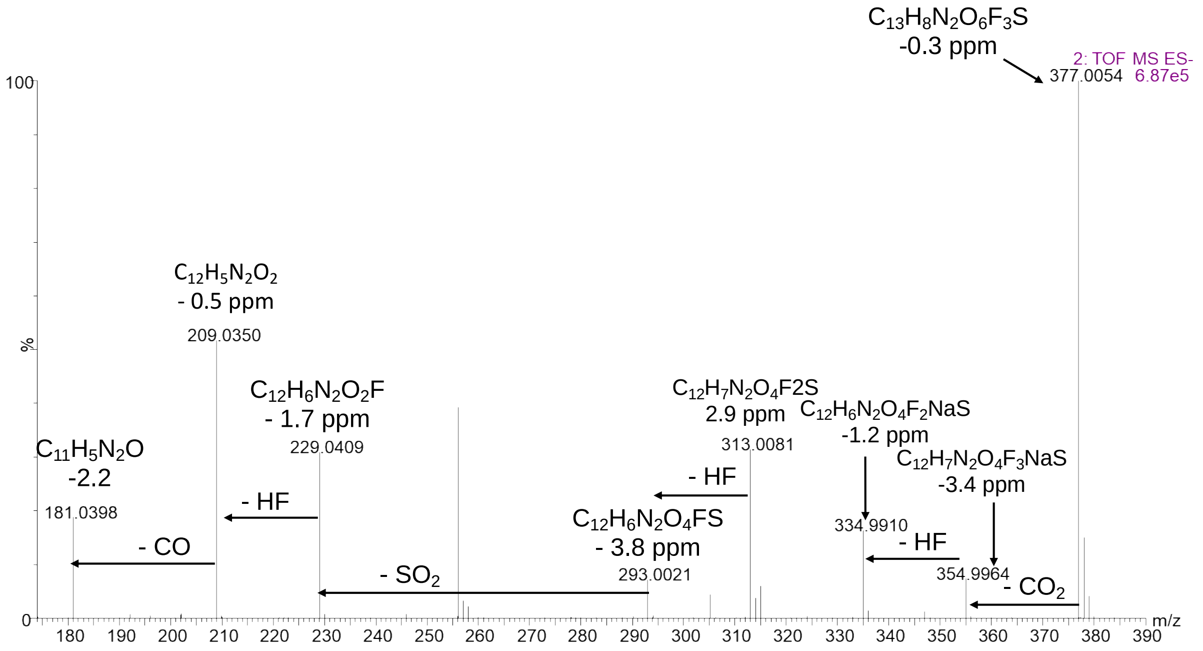

Compound ID: NA_7

Compound name: Niflumic acid + Gluc + 2H

Chemical formula: $C_{19}H_{18}N_2O_8F_3$

m/z: 459.1026 (-)

Retention time: 5.87 min (-)

Confidence level: 3

XIC:

Negative mode:

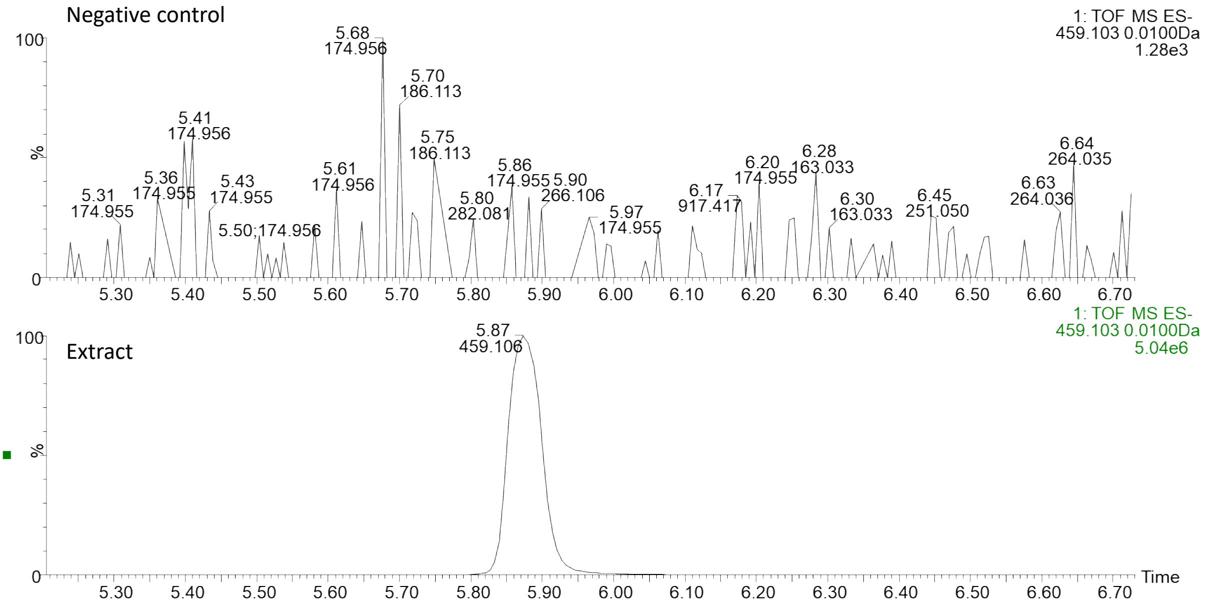

MS/MS spectrum:

Negative mode

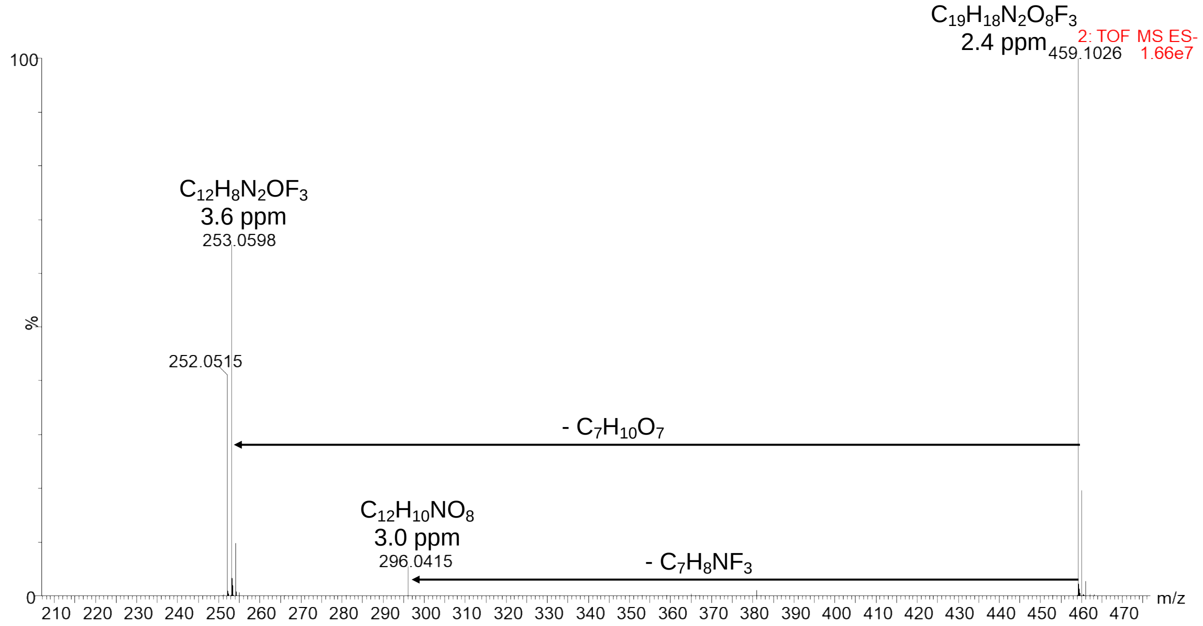

Compound ID: NA_8

Compound name: Niflumic acid + Me

Chemical formula: $C_{14}H_{12}N_2O_2F_3$ (+)

m/z: 297.0850

Retention time: 11.66 min (+)

Confidence level: 3

Proposed chemical structure:

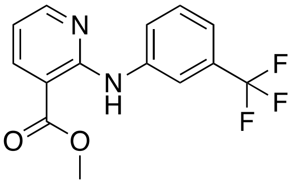

XIC:

Positive mode:

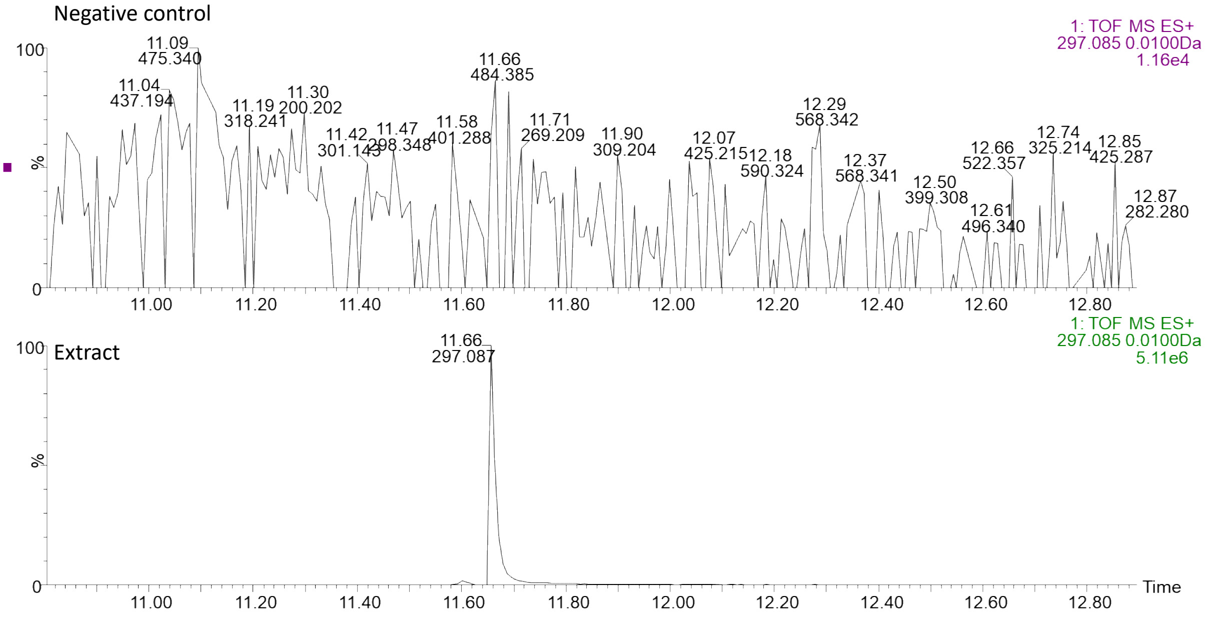

MS/MS spectrum:

Positive mode

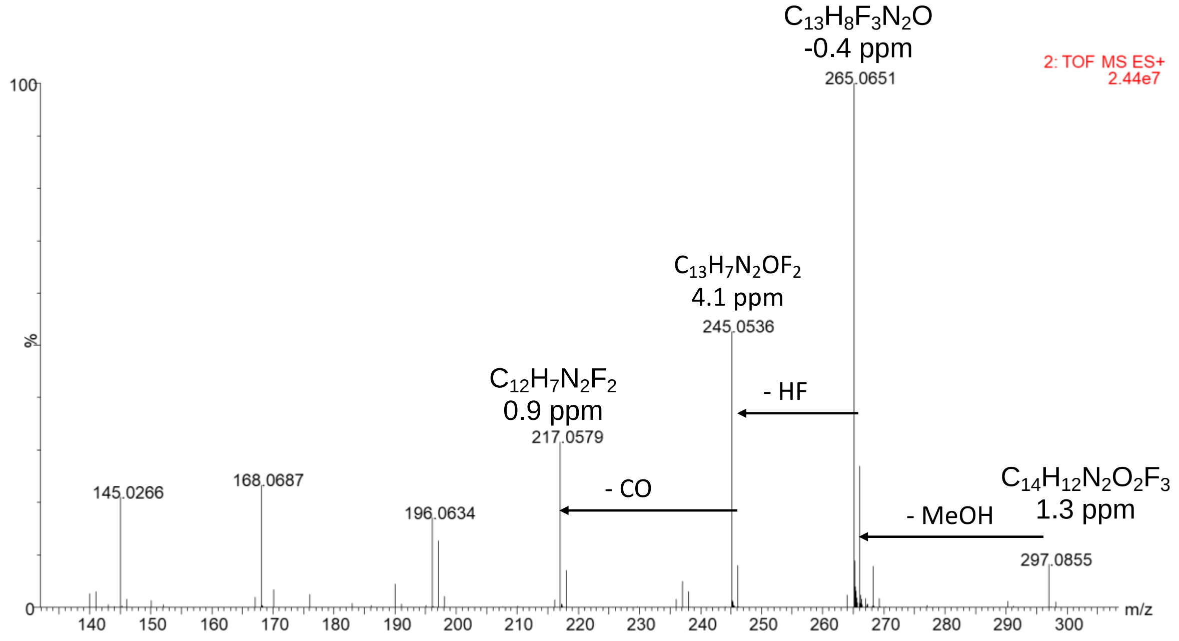

11. References

(1) Grasse, N.; Seiwert, B.; Massei, R.; Scholz, S.; Fu, Q.; Reemtsma, T. Uptake and Biotransformation of the Tire Rubber-Derived Contaminants 6-PPD and 6-PPD Quinone in the Zebrafish Embryo (*Danio Rerio*). *Environ. Sci. Technol.* **2023**, acs.est.3c02819. https://doi.org/10.1021/acs.est.3c02819.

(2) Kimmel, C. B.; Ballard, W. W.; Kimmel, S. R.; Ullmann, B.; Schilling, T. F. Stages of Embryonic Development of the Zebrafish. *Dev. Dyn.* **1995**, *203* (3), 253–310. https://doi.org/10.1002/aja.1002030302.

(3) Bittner, L.; Klüver, N.; Henneberger, L.; Mühlenbrink, M.; Zarfl, C.; Escher, B. I. Combined Ion-Trapping and Mass Balance Models To Describe the pH-Dependent Uptake and Toxicity of Acidic and Basic Pharmaceuticals in Zebrafish Embryos (*Danio Rerio*). *Environ. Sci. Technol.* **2019**, *53* (13), 7877–7886. https://doi.org/10.1021/acs.est.9b02563.

(4) Goss, K.-U.; Bittermann, K.; Henneberger, L.; Linden, L. Equilibrium Biopartitioning of Organic Anions – A Case Study for Humans and Fish. *Chemosphere* **2018**, *199*, 174–181. https://doi.org/10.1016/j.chemosphere.2018.02.026.

(5) Henneberger, L.; Goss, K.-U.; Endo, S. Equilibrium Sorption of Structurally Diverse Organic Ions to Bovine Serum Albumin. *Environ. Sci. Technol.* **2016**, *50* (10), 5119–5126. https://doi.org/10.1021/acs.est.5b06176.

(6) Halbach, K.; Ulrich, N.; Goss, K.-U.; Seiwert, B.; Wagner, S.; Scholz, S.; Luckenbach, T.; Bauer, C.; Schweiger, N.; Reemtsma, T. Yolk Sac of Zebrafish Embryos as Backpack for Chemicals? *Environ. Sci. Technol.* **2020**, *54* (16), 10159–10169. https://doi.org/10.1021/acs.est.0c02068.

(7) Huchthausen, J.; Braasch, J.; Escher, B. I.; König, M.; Henneberger, L. Effects of Chemicals in Reporter Gene Bioassays with Different Metabolic Activities Compared to Baseline Toxicity. *Chem. Res. Toxicol.* **2024**, *37* (5), 744–756. https://doi.org/10.1021/acs.chemrestox.4c00017.

(8) Klüver, N.; Vogs, C.; Altenburger, R.; Escher, B. I.; Scholz, S. Development of a General Baseline Toxicity QSAR Model for the Fish Embryo Acute Toxicity Test. *Chemosphere* **2016**, *164*, 164–173. https://doi.org/10.1016/j.chemosphere.2016.08.079.

(9) Verhaar, H. J. M.; Van Leeuwen, C. J.; Hermens, J. L. M. Classifying Environmental Pollutants. *Chemosphere* **1992**, *25* (4), 471–491. https://doi.org/10.1016/0045-6535(92)90280-5.

(10) Maeder, V.; Escher, B. I.; Scheringer, M.; Hungerbühler, K. Toxic Ratio as an Indicator of the Intrinsic Toxicity in the Assessment of Persistent, Bioaccumulative, and Toxic Chemicals. *Environ. Sci. Technol.* **2004**, *38* (13), 3659–3666. https://doi.org/10.1021/es0351591.

(11) Brox, S.; Ritter, A. P.; Küster, E.; Reemtsma, T. A Quantitative HPLC–MS/MS Method for Studying Internal Concentrations and Toxicokinetics of 34 Polar Analytes in Zebrafish (Danio Rerio) Embryos. *Anal Bioanal Chem* **2014**, *406* (20), 4831–4840. https://doi.org/10.1007/s00216-014-7929-y.

(12) Schirmer, K.; Tanneberger, K.; Kramer, N.; Volker, D.; Scholz, S.; Hafner, C.; Lee, L.; Bols, N.; Hermens, J. Developing a List of Reference Chemicals for Testing Alternatives to Whole Fish Toxicity Tests. *Aquatic Toxicology* **2008**, *90* (2), 128–137. https://doi.org/10.1016/j.aquatox.2008.08.005.

(13) Klüver, N.; Bittermann, K.; Escher, B. I. QSAR for Baseline Toxicity and Classification of Specific Modes of Action of Ionizable Organic Chemicals in the Zebrafish Embryo Toxicity Test. *Aquatic Toxicology* **2019**, *207*, 110–119. https://doi.org/10.1016/j.aquatox.2018.12.003.

(14) Lomba, L.; Ribate, M. P.; Zuriaga, E.; García, C. B.; Giner, B. Acute and Subacute Effects of Drugs in Embryos of Danio Rerio. QSAR Grouping and Modelling. *Ecotoxicology and Environmental Safety* **2019**, *172*, 232–239. https://doi.org/10.1016/j.ecoenv.2019.01.081.

(15) Yen, J.; Donerly, S.; Levin, E. D.; Linney, E. A. Differential Acetylcholinesterase Inhibition of Chlorpyrifos, Diazinon and Parathion in Larval Zebrafish. *Neurotoxicology and Teratology* **2011**, *33* (6), 735–741. https://doi.org/10.1016/j.ntt.2011.10.004.

(16) Andrade, T. S.; Henriques, J. F.; Almeida, A. R.; Machado, A. L.; Koba, O.; Giang, P. T.; Soares, A. M. V. M.; Domingues, I. Carbendazim Exposure Induces Developmental, Biochemical and Behavioural Disturbance in Zebrafish Embryos. *Aquatic Toxicology* **2016**, *170*, 390–399. https://doi.org/10.1016/j.aquatox.2015.11.017.

(17) Xia, L.; Zheng, L.; Zhou, J. L. Effects of Ibuprofen, Diclofenac and Paracetamol on Hatch and Motor Behavior in Developing Zebrafish (Danio Rerio). *Chemosphere* **2017**, *182*, 416–425. https://doi.org/10.1016/j.chemosphere.2017.05.054.

(18) Wang, X.; Li, X.; Wang, Y.; Qin, Y.; Yan, B.; Martyniuk, C. J. A Comprehensive Review of Strobilurin Fungicide Toxicity in Aquatic Species: Emphasis on Mode of Action from the Zebrafish Model. *Environmental Pollution* **2021**, *275*, 116671. https://doi.org/10.1016/j.envpol.2021.116671.

(19) Huchthausen, J.; Escher, B. I.; Grasse, N.; König, M.; Beil, S.; Henneberger, L. Reactivity of Acrylamides Causes Cytotoxicity and Activates Oxidative Stress Response. *Chem. Res. Toxicol.* **2023**, *36* (8), 1374–1385. https://doi.org/10.1021/acs.chemrestox.3c00115.
